# Techno-economic and environmental assessment of next-generation battery-powered farming robots

**DOI:** 10.1038/s41467-026-78281-7

**Published:** 2026-11-02

**Authors:** Simon Schlehuber, Moritz Gutsch, Luca Stegemann, Thomas Hendrickson, Stephan von Delft

**Affiliations:** 1 Institute of Business Chemistry, University of Münster, Münster, Germany; 2 Department of Civil and Environmental Engineering, University of California Davis, Davis, CA USA; 3 Adam Smith Business School, University of Glasgow, Glasgow, UK

**Keywords:** Environmental impact, Agriculture, Technology, Environmental economics

## Abstract

Decarbonizing agriculture without compromising productivity is a major challenge under rising input costs and labor scarcity. Autonomous battery-powered agricultural robots (ABARs) could help by reducing dependence on conventional machinery and enabling more precise application of inputs, but their system-level economic and environmental performance remains unclear. Here, we integrate total cost of ownership and life cycle assessment to evaluate a small ABAR for cradle-to-farm-gate crop production of strawberries, onions, lettuce, and peanuts under organic and non-organic management. Across modeled scenarios, benchmarked costs range from 76% below to 17% above literature-based reference ranges, while greenhouse-gas emissions fall within or below reported ranges. Variable inputs dominate both outcomes, contributing 72–90% of costs and 72–95% of global-warming impacts, with seeds, fertilizers, and irrigation water as main drivers. These results suggest that ABAR value depends less on the platform than on the ability of autonomous operation to reduce input use and labor demand.

## Abstract (web summary)

This study compares a small autonomous battery-powered farming robot with conventional farming and finds competitive costs and low greenhouse-gas emissions, driven mainly by reductions in labor and input use.

## Introduction

Agriculture faces growing pressure to increase output while reducing emissions and resource use. Food systems account for more than one-fifth of global greenhouse gas emissions^1^, while agricultural production is increasingly constrained by climate change, soil degradation, and rising and uncertain input costs, particularly in energy and fertilizer markets^2,3^. At the same time, demand for agricultural products is projected to rise by up to 50% until 2050^4^. Meeting this challenge is central to several United Nations Sustainable Development Goals (SDGs), including zero hunger (SDG 2), responsible consumption and production (SDG 12), and climate action (SDG 13)^5,6^, and is increasingly framed as part of broader efforts to keep food production within planetary and resource boundaries^7–10^.

Against this backdrop, digital and automated technologies are progressively being advanced as routes to more resource-efficient agriculture^11–13^. Autonomous agricultural robots, in particular, could improve safety, reduce labor requirements, and apply inputs more precisely than conventional machinery, with potential benefits for both costs and environmental impacts^13–16^. Rapid advances in battery-electric propulsion, charging concepts, and artificial intelligence (AI) have accelerated their technical development^17–19^. Yet, it remains unclear under which conditions these systems deliver meaningful economic and environmental gains. Their deployment depends not only on technical feasibility, but also on reliability, maintenance, charging logistics, infrastructure, regulation, safety, and operator capabilities^18,20^.

Among these emerging systems, autonomous battery-powered agricultural robots (ABARs) are especially relevant for small to medium-sized farms and for crops in which irrigation, fertilization, and weed control remain important drivers of costs and emissions. By combining compact electric platforms with sensors and automated control, ABARs may complement or replace conventional machinery in selected field operations while enabling more targeted application of water and agrochemicals. Like other clean technologies at an early stage of development, ABARs still face technical, operational, and regulatory challenges. Their potential to reduce both greenhouse gas emissions and production costs, therefore, needs to be assessed at the crop-system level.

Evidence to date remains fragmented. Existing studies have assessed specific robots, regions, or individual operations, or have focused on either environmental or economic performance in isolation^20–24^. As a result, ABARs remain insufficiently characterized at the crop-production-system level when environmental and economic outcomes are evaluated together within a single, consistent framework spanning multiple field operations.

**Fig. 1:**
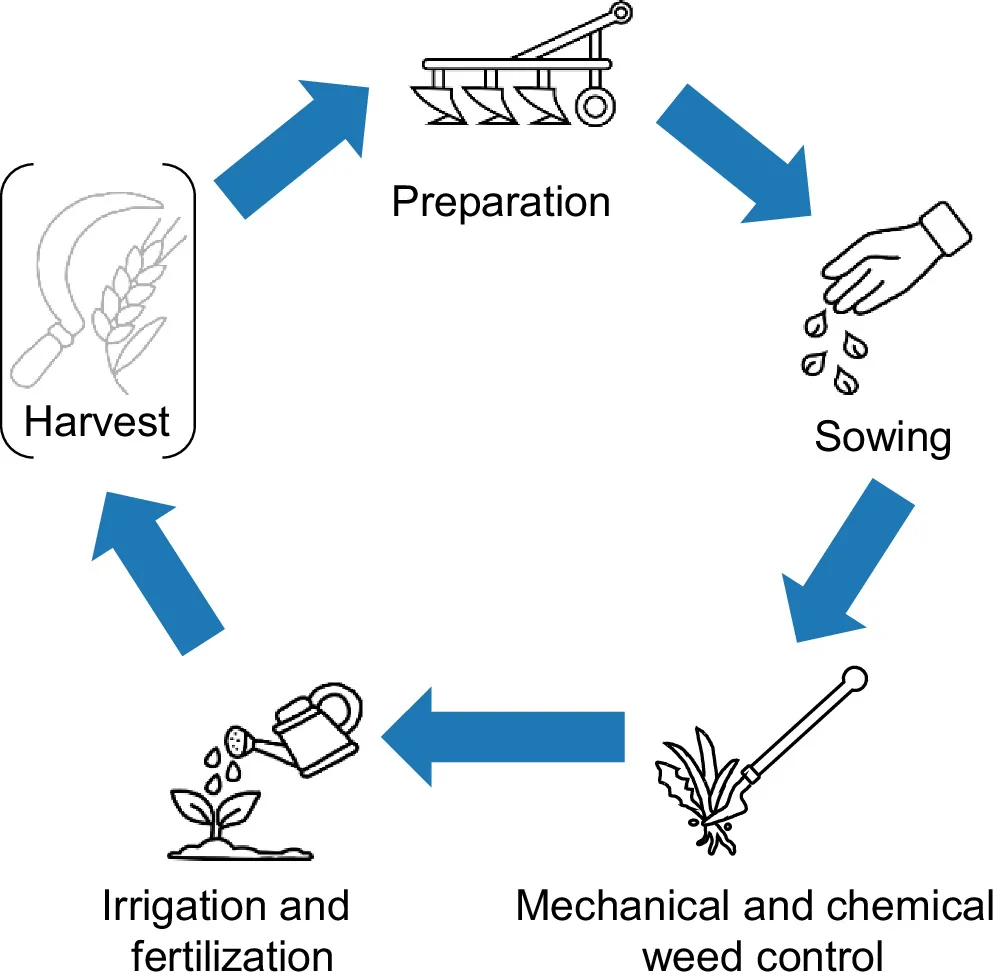
System boundary of the assessment. The system boundary includes the four tasks: preparation, sowing, mechanical and chemical weed control, and irrigation and fertilization. These are modeled for all crops considered in this study: strawberries, onions, lettuce, and peanuts. Harvesting lies outside the modeled platform scope because practices differ substantially between crops.

Here, we develop a parameterized ABAR model and integrate comprehensive life cycle assessment (LCA) and total cost of ownership (TCO) for cradle-to-farm-gate production of strawberries, onions, lettuce, and peanuts under organic and non-organic management. The model includes simplified tool and module proxies for preparation, sowing, mechanical and chemical weed control, irrigation, and fertilization, which together define the cradle-to-farm-gate system boundary summarized in Fig. 1. Harvesting is excluded from the ABAR platform model because harvesting technologies, labor requirements, and mechanization levels are strongly crop-specific and, for several of the crops considered, remain partly manual^25–28^. We quantify costs and greenhouse-gas emissions per kilogram of crop and benchmark against reported ranges for conventional systems. This framework allows us to identify where ABARs may already be competitive, where their performance remains constrained, and whether their benefits arise primarily from the robot platform itself or from reductions in variable inputs and labor demand.

## Results

### Model of the automated farming robot

**Fig. 2:**
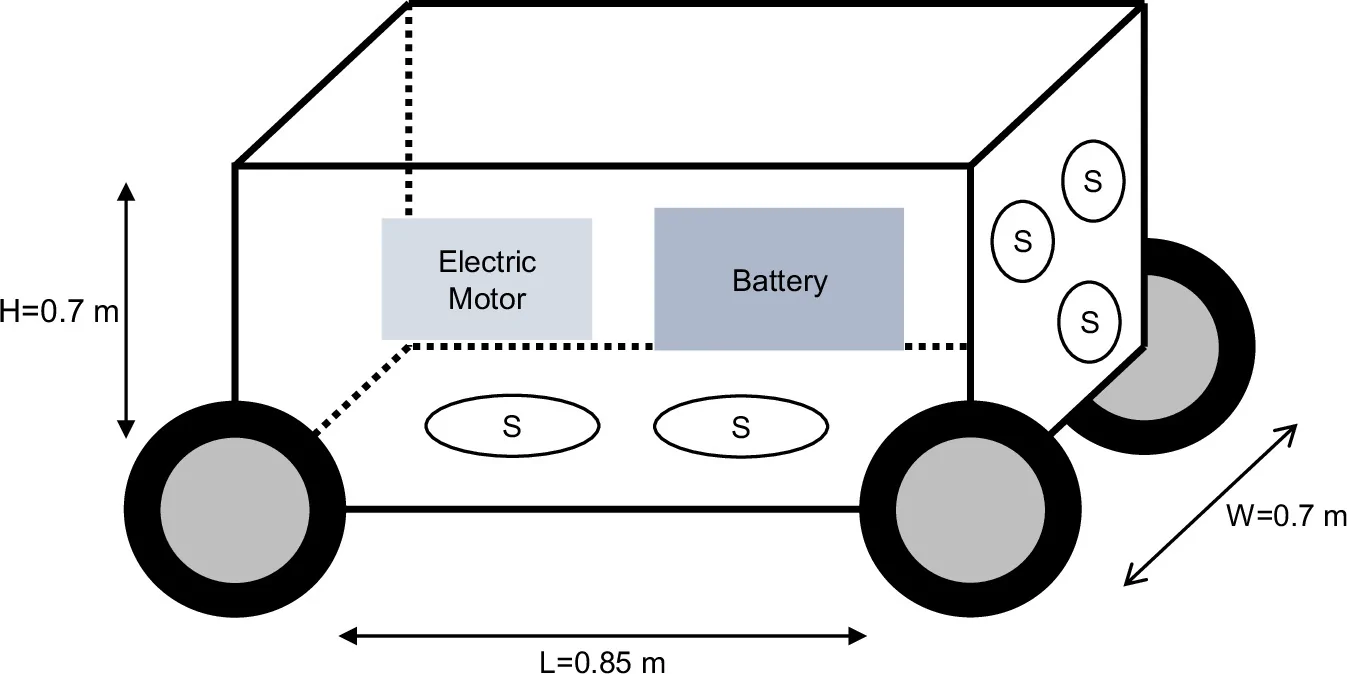
Schematic drawing of the modeled autonomous battery-powered farming robot (S = sensor, H = height, L = length, W = width). The robot is modeled to include a chassis, an electric motor, a battery, four wheels, and five different types of sensors (see Supplementary Table 1 for more details). It is 0.85 meters (m) long, 0.7 m wide, and 0.7 m high.

The ABAR model in this study reflects recent industrial autonomous farming robots^29,30^, but is intended as a simplified, parameterized representation rather than a one-to-one model of any specific product (Fig. 2). Its dimensions (0.85 meters (m) × 0.70 m × 0.70 m; Length × Width × Height) are based on an existing compact ABAR platform^29^ to anchor the geometry, while the included components, their sizing, and the operational parameterization were defined for this study based on the assumptions described in “Methods”. The robot runs on wheels and incorporates a battery-electric drivetrain sized for high-power operations such as field preparation. Onboard perception and positioning are provided by a camera, global positioning system (GPS), and lidar (light detection and ranging), with embedded computing, enabling autonomous control. The robot’s weight is 200 kilograms (kg), excluding task-specific tools and ballast. The model is structured so that alternative ABAR designs can be evaluated by substituting platform parameters such as working width, component masses, battery capacity, and task speeds.

To estimate task-level emissions and costs more accurately, the required tools are represented by simplified proxies. Where appropriate, these are scaled to the robot’s geometry and informed by existing technologies. They include a proxy for soil preparation, mountable seeding units, a cultivator for mechanical weed control, a boom sprayer for chemical weed control, and a trailer-mounted water bowser for irrigation. Fertilization is assumed to occur concurrently with irrigation and therefore does not require an additional tool. Further details on the robot, ballast, and proxy tools are provided in Supplementary Table 1. Unless stated otherwise, model inputs are compiled from literature and database sources and applied within the system of equations described in “Methods”. Other parameters reflect the modeling choices required for the scenarios, with all values documented in Supplementary Tables 15–21.

### Total cost of ownership analysis

The TCO framework quantifies the levelized cost per kilogram of crop (LCPK), enabling direct comparison across crops and production systems. LCPK comprises four components: (i) annualized capital expenditures (CAPEX), (ii) operational expenditures (OPEX), (iii) annualized end-of-life recycling costs, and (iv) a maintenance factor. CAPEX includes the battery, electric motor, wheels, sensors, chassis, steel ballast, and task-specific proxy tools of the ABAR. OPEX includes electricity, seeds, pesticides, water, fertilizer, and labor. Although the robot is modeled as autonomous during field operations, labor costs are not assumed to be zero because operation still requires setup, supervision, occasional intervention, and logistical support^24,31^. Recycling costs account for the disposal and processing of end-of-life materials from the ABAR, ballast, and proxy tools, including the battery, aluminum, steel, rubber, and linear low-density polyethylene. Maintenance is modeled as a fixed percentage of CAPEX.

To express all cost components on a common annual basis, CAPEX and recycling costs are annualized using a capital-recovery approach and the robot’s lifetime. These annualized costs, together with OPEX and maintenance, are then divided by the expected annual yield for each crop and production system (organic and non-organic), yielding the LCPK. This framework provides a transparent link between physical assets, operating inputs, and output. At the same time, the TCO analysis is limited to direct, modeled cost components and does not capture broader techno-economic dynamics such as organizational change on farms, learning effects and cost reductions over time, or risk and uncertainty beyond the stated assumptions. These factors may influence implementation and realized costs, but they fall outside the scope of the quantitative TCO model. Further methodological details on the system of equations and relevant parameters are provided in “Methods”. All results are generated by applying these equations to the compiled parameter set (see Supplementary Tables 15–21) rather than from on-farm measurements of a deployed robot.

### Life cycle assessment

Consistent with the TCO analysis, the LCA is expressed as a levelized impact per kilogram of crop. This impact is calculated by combining fixed burdens from producing the ABAR and task-specific proxy tools, variable burdens from operation and agricultural inputs, end-of-life burdens from recycling or disposal, and a maintenance factor. These fixed, variable, and end-of-life components follow the same general structure as the CAPEX, OPEX, and recycling terms used in the cost analysis, with the exception of labor. Labor is excluded from the LCA because it is not typically treated as a direct emissions-generating flow^32^. Fixed and end-of-life burdens are annualized using the impact recovery factor, whereas variable burdens arise with use. The resulting total annual impact is then divided by the expected annual yield for each crop and production system to obtain the levelized value. The analysis focuses on the 100-year global warming potential (GWP), as it is among the most widely discussed impact categories and highly relevant for policy^33–35^; results for additional impact categories are reported in Supplementary Tables 2–4. As in the TCO analysis, the LCA quantifies environmental impacts attributable to the defined life cycle flows within the system boundary but does not capture changes over time associated with learning, behavioral adaptation, or organizational change as deployment scales. Further details are provided in “Methods”.

### Results for the combined life cycle and cost assessment

To assess the applicability of the ABAR across different farming contexts, we construct eight scenarios covering four field crops—strawberries, onions, lettuce, and peanuts—each under organic and non-organic production systems. Together, these scenarios span contrasting agricultural practices, task frequencies, and per-hectare (ha) input requirements, providing a broad basis for comparison. Evaluating the ABAR in terms of both cost (TCO) and environmental impact (GWP) yields complementary metrics that are relevant to the SDGs, including SDG 2 (Zero Hunger) through potential cost reductions and SDG 13 (Climate Action) through the potential to lower emissions.

Across scenarios, variable input requirements and prices differ substantially by crop and production system. Quantities and prices for seed, pesticides, fertilizer, and irrigation water are drawn from publicly available sources. Each crop–system combination is modeled with its own input profile, while robot characteristics and operating assumptions are held constant across scenarios. Geographic representativeness is approximated by focusing on conditions common to the southern and western regions of Europe and the United States, while recognizing that local climate and management heterogeneity cannot be fully captured within a single model.

**Fig. 3:**
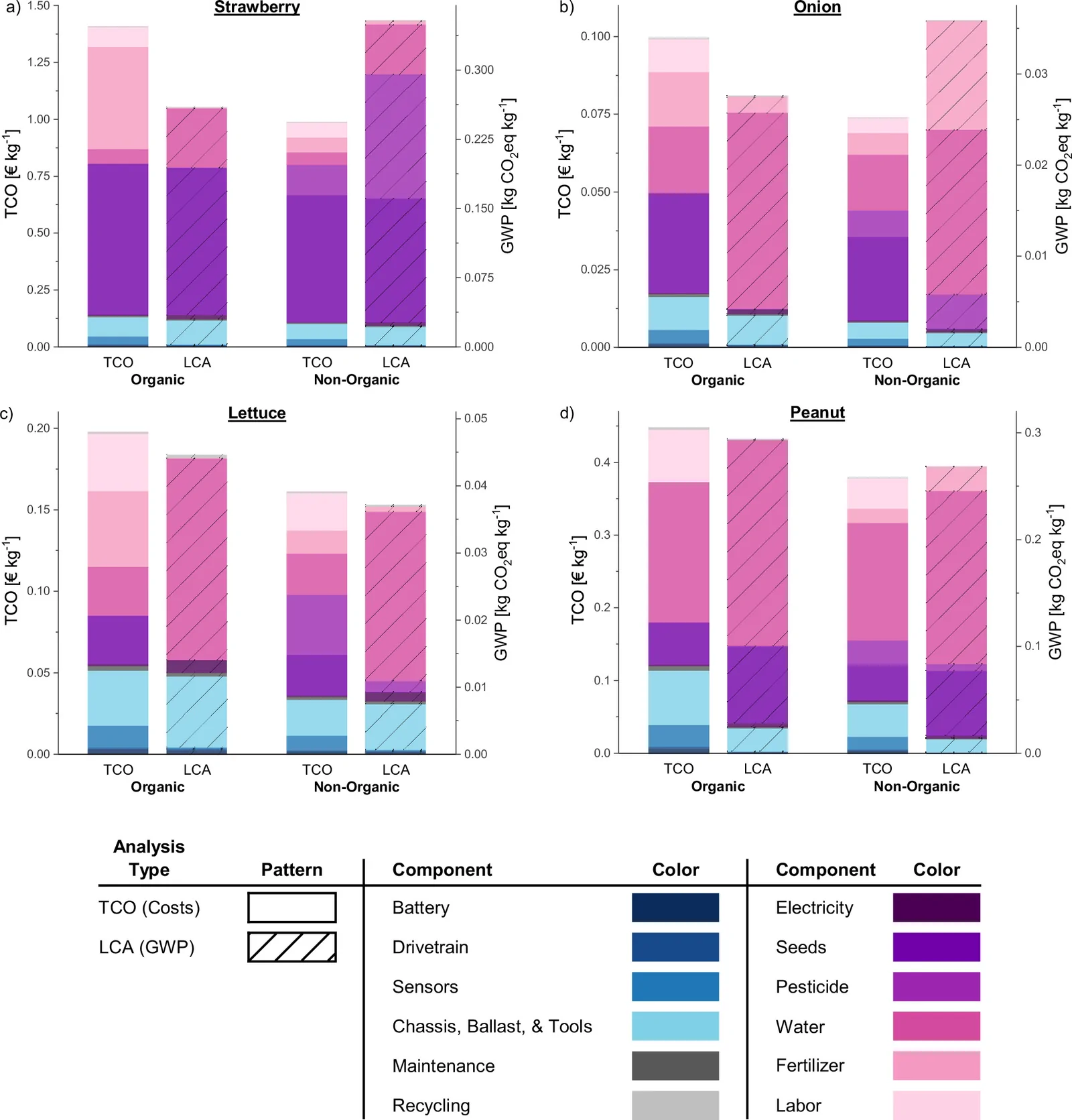
Results of the life cycle assessment (LCA) and total cost of ownership (TCO) analysis for each crop and production system using the modeled autonomous battery-powered agricultural robot. Modeled tasks include preparation, sowing, weed control, irrigation, and fertilization. Impacts and costs of fixed components are shown in blue tones, variable components in purple, and maintenance and recycling in gray. Panels show (**a**) strawberries, (**b)** onions, (**c)** lettuce, and (**d)** peanuts.

Key operating parameters include a daily operating time of 18 h, a task-specific working width ranging from 0.2 to 0.7 m, and the task-specific field speeds defined in “Methods”. Labor is treated separately in the TCO model through a supervision factor rather than being assumed to scale one-to-one with robot operating time. Background data, parameter values, and sources are provided in Supplementary Tables 15–21. The resulting TCO and GWP outcomes for all eight scenarios are summarized in Fig. 3.

From a TCO perspective, onions are the least expensive crop per kilogram in both systems (organic (O): 0.10 Euro per kilogram (€ kg^−1^), non-organic (NO): 0.07 € kg^−1^). Lettuce follows (O: 0.20 € kg^−1^, NO: 0.16 € kg^−1^), then peanuts (O: 0.45 € kg^−1^, NO: 0.38 € kg^−1^). By contrast, strawberries are the most expensive crop in both systems (O: 1.41 € kg^−1^, NO: 0.99 € kg^−1^). Overall, organic production is associated with higher TCO than non-organic production across all crops. This difference is driven primarily by lower yields per hectare and higher fertilizer costs, which more than offset the absence of pesticide use in the organic systems.

For life cycle impacts expressed as GWP, the pattern differs. Onions exhibit the lowest GWP in both systems (O: 0.028 kilograms carbon-dioxide equivalent (kg CO_2_ eq kg^−1^), NO: 0.036 kg CO_2_ eq kg^−1^), followed by lettuce (O: 0.045 kg CO_2_ eq kg^−1^, NO: 0.037 kg CO_2_ eq kg^−1^). In the organic system, strawberries show lower GWP than peanuts (0.260 vs. 0.294 kg CO_2_ eq kg^−1^), whereas in the non-organic system peanuts have lower GWP than strawberries (0.269 vs. 0.354 kg CO_2_ eq kg^–1^).

No uniform difference in GWP emerges between organic and non-organic systems across crops. Instead, the pattern is shaped by crop-specific input profiles and yields. In most scenarios, irrigation water is the dominant contributor to GWP because it is applied in much larger quantities than any other input. Although its impact per kilogram is low, its large volume makes it the largest contributor overall. Because organic systems have lower yields per hectare, this water-related burden is distributed across fewer kilograms of output, increasing GWP per kilogram. For lettuce and peanuts, these water-related impacts are not fully offset by the absence of pesticide use or by the use of less climate-intensive fertilizers in the organic system. Strawberries and onions deviate from this pattern because the impacts of other components also play a stronger role. For strawberries, the contribution of propagated planting material substantially raises overall GWP, thereby reducing the relative importance of irrigation water. In addition, non-organic strawberries are associated with intensive pesticide use, which contributes strongly to GWP, while the absence of pesticide impacts in the organic system lower GWP relative to the non-organic case. For onions, by contrast, the lower fertilizer-related impacts in the organic system are sufficient to outweigh the otherwise important contribution of irrigation water.

Finally, across all crops and production systems, the decomposition shows that variable components (purple bars in Fig. 3) dominate both TCO and GWP, accounting for 72–90% of TCO and 72–95% of GWP, respectively. For TCO, the largest contributions arise from water, seeds, fertilizers, and labor. For GWP, the main contributions are water, seeds, and fertilizers. By contrast, fixed components (blue bars in Fig. 3) are driven mainly by the chassis, the ballast, and the proxy tools of the ABAR, while maintenance and recycling contribute only marginally throughout.

### Sensitivity analysis of cost and life cycle assessment

**Fig. 4:**
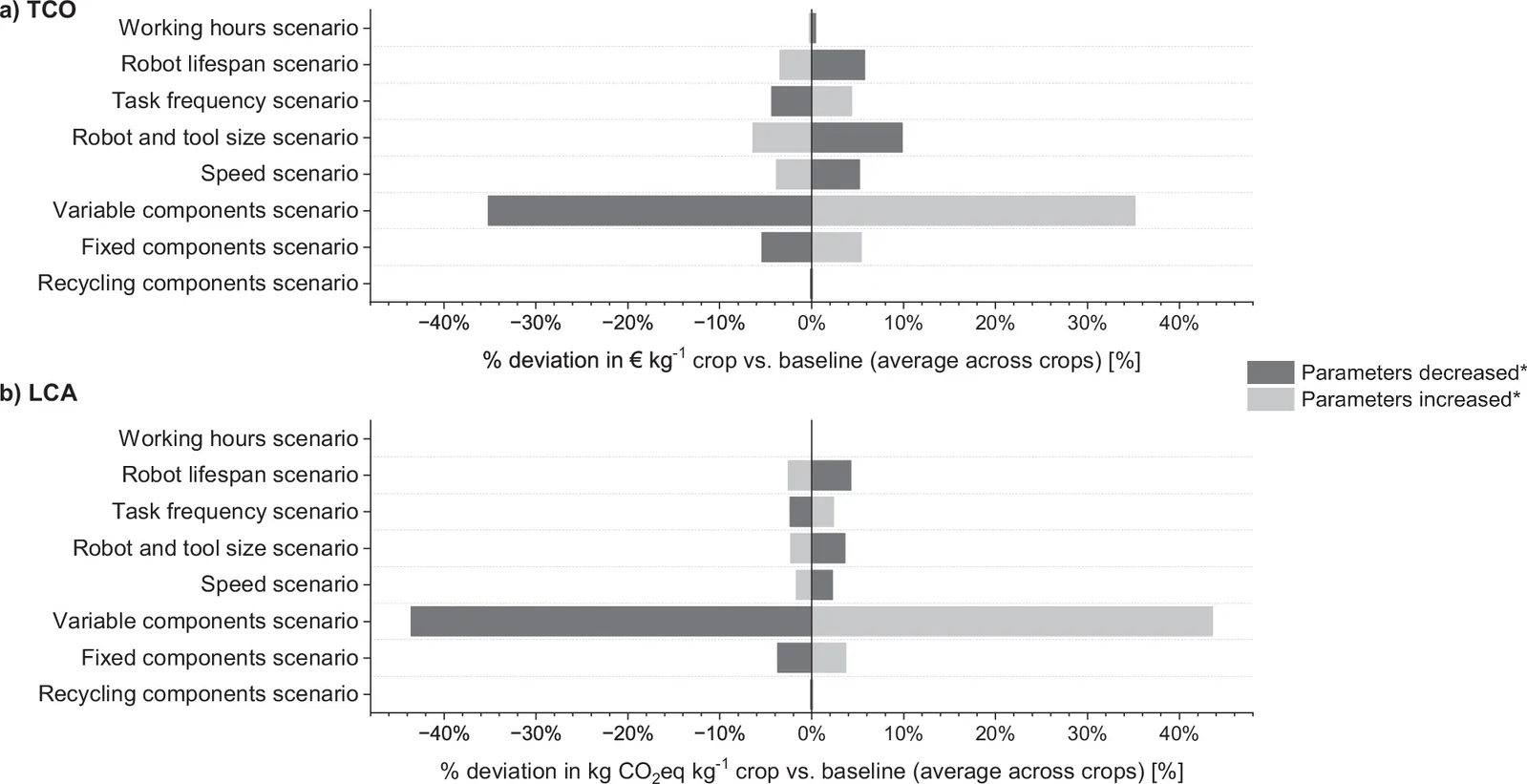
Sensitivity analysis results for total cost of ownership (TCO) and life cycle assessment (LCA). Each scenario represents a group of parameters varied simultaneously while all other parameters are held constant. Panel **a**) shows sensitivity analysis results for TCO while panel **b**) shows sensitivity analysis results for LCA. *Category-specific variation ranges were applied to the parameters to reflect differing engineering, operational, and agronomic plausibility across parameter groups (see Table 1).

To account for uncertainty in key parameters and to ensure transparency, a sensitivity analysis was performed across all crops, production systems, and both outcome metrics (TCO and GWP). Given the large number of parameters, conceptually related inputs were grouped and varied jointly. This yielded eight scenarios: working hours, robot lifespan, task frequency, robot size, speed, variable components, fixed components, and recycling components. For each scenario, and for both TCO and LCA, the relevant parameter groups (see Table 1 for an overview) were varied around their baseline values while all other parameters were held constant. The applied variation ranges differed by scenario, with narrower bounds for parameters more tightly constrained by engineering design or operating feasibility and broader bounds for variable input parameters. This produced absolute TCO and GWP values for each crop and scenario, which were then expressed as percentage deviations from the respective base case. These deviations were subsequently averaged across crops to obtain a single mean percentage deviation per scenario. The resulting aggregate sensitivities are shown in Fig. 4.

**Table 1:** Overview of the parameters varied simultaneously in each sensitivity analysis scenario for total cost of ownership (TCO) and life cycle assessment (LCA)

Name of scenario	Parameters varied	Variation compared to base value
Working hours scenario	**TCO & LCA**: *h*	±25%
Robot lifespan scenario	**TCO & LCA**: *lifespan*_Robot_	±25%
Task frequency scenario	**TCO & LCA:** *x*_Weed control, crop_, *x*_Watering, crop_	±25%
Robot and tool size scenario	**TCO & LCA:** *W*_Robot_, *L*_Robot_, *H*_Robot_, *W*_Tool_, *S*_Tool_	±15%
Speed scenario	**TCO & LCA:** *v*_Preparation,_ *v*_Sowing,_ *v*_Mechanical weed control,_ *v*_Chemical weed control,_ *v*_Watering_	±15%
Variable components scenario	**TCO:** *c*_Electricity, specific_, *c*_Seeds, specific, crop_, *c*_Pesticide, specific, crop_, *c*_Water, specific_, *c*_Fertilizer, specific, crop_ **LCA:** Total electricity consumption per year_crop_, *q*_Seeds, crop_, *m*_Pesticide, crop_, *m*_Water, crop_, *m*_Fertilizer (N, P2O5, K2O), crop_	±50%
Fixed components scenario	**TCO:** *c*_Battery, specific_, *c*_Electric motor, specific_, *c*_Wheels, specific_, *c*_Sensors,_ *c*_Aluminum, specific_, *c*_Steel, specific_, *c*_Tools_ **LCA:** capacity_Battery_, *m*_Electric motor_, *m*_Rubber_, *m*_Aluminum_, *m*_Steel_, *m*_LLDPE_	±30%
Recycling components scenario	**TCO:** *c*_Battery recycling, specific_, *c*_Wheel recycling, specific_, *c*_Aluminum recycling, specific_, *c*_Steel recycling, specific,_ *c*_LLDPE Recyling, specific_ **LCA:** *m*_Battery, end-of-life_, *m*_Aluminum, end-of-life_, *m*_Steel, end-of-life_, *m*_Rubber, end-of-life_, *m*_LLDPE, end-of-life_	±30%

Category-specific variation ranges were applied to reflect differing engineering, operational, and agronomic plausibility across parameter groups. See “Methods” for a description of all parameters.

Because the applied variation ranges differ across scenarios, the resulting deviations reflect both model responsiveness and the size of the variation range. Under this setup, the variable components scenario, varied by ±50%, produces the largest deviations from the baseline for both TCO ( ± 35%) and GWP ( ± 44%). This is consistent with the dominant contribution of variable inputs to both outcomes. It further indicates that changes in the prices, quantities, or application rates of seeds, fertilizers, pesticides, water, and electricity strongly affect overall TCO and GWP. The robot and tool size scenario, despite being varied by only ±15%, also shows a clear influence (TCO: +10%/−6%; GWP: +4%/−2%), underlining the importance of the platform’s physical configuration. The speed scenario, likewise varied by ±15%, produces noticeable deviations (TCO: +5%/−4%; GWP: +2%/−2%), while the robot lifespan scenario, varied by ±25%, also affects both outcomes (TCO: +6%/−3%; GWP: +4%/−3%). Task frequency, varied by ±25%, and fixed components, varied by ±30%, lead to more moderate deviations, whereas changes in working hours ( ± 25%) and recycling components ( ± 30%) have only a minor effect on the results. Overall, TCO and GWP respond in broadly similar directions across scenarios, indicating that the main economic drivers are also the main drivers of environmental impacts.

### Benchmarking farming robot-based production against conventional farming

Benchmarking the ABAR against conventional crop production is necessary to assess whether the modeled system offers practically relevant advantages in terms of costs and environmental impacts. The following analysis, therefore, compares ABAR-based production with literature-based reference ranges for conventional systems using published data for both TCO and LCA. Because most conventional cradle-to-farm-gate estimates include harvesting, and because harvesting can represent a substantial share of total production costs and emissions, harvesting is explicitly considered in this benchmarking step to enable a consistent comparison.

**Fig. 5:**
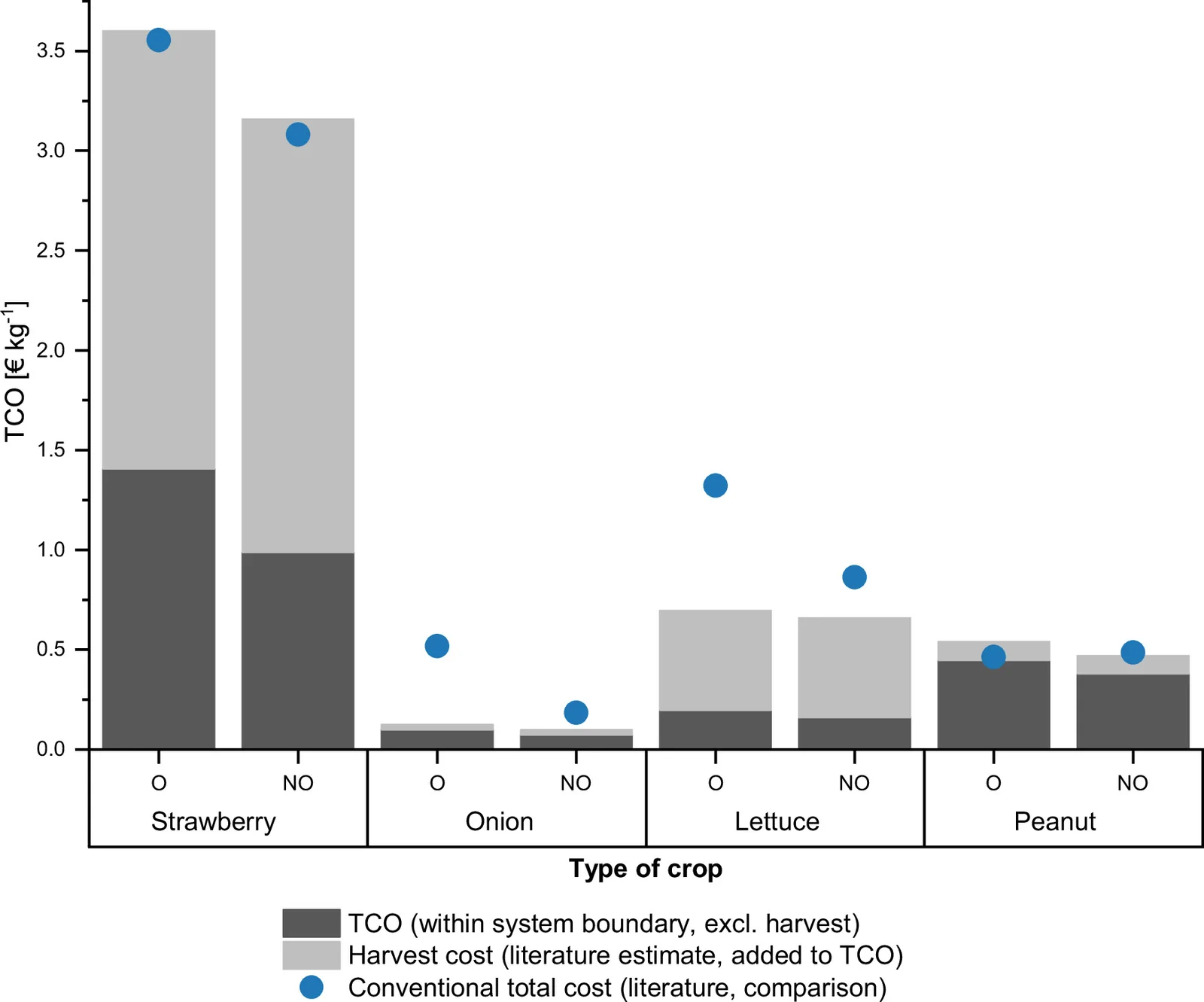
Total cost of ownership (TCO) per kg by crop for the farming-robot system versus conventional production. Stacked bars show TCO within the system boundary (preparation, sowing, weed control, irrigation, fertilizer) with a literature-based harvest cost added to yield the final value. Dots indicate literature values for conventional total cost per kg crop used for comparison. O organic, NO non-organic.

The comparison first focuses on TCO. Because harvesting is excluded from the ABAR platform model, it is assumed to be carried out conventionally for each crop. Figure 5 therefore reports the ABAR TCO per kilogram without harvesting (dark gray bars) together with crop-specific harvesting costs per kilogram from previous studies (light gray bars)^36–39^. The sum of these two components represents the full cradle-to-farm-gate cost of ABAR-based production and is compared with reported TCO values for conventional crop production (blue markers)^36–43^. For organic onions and lettuce, the underlying reference costs were adjusted for inflation because no recent data were available.

Figure 5 shows that the cost competitiveness of the ABAR relative to conventional production depends on the crop once harvesting is included. For onions and lettuce, the ABAR-based system remains below the conventional reference in both production systems, with the clearest differences for organic onions (ABAR: 0.13 € kg^−1^, conventional farming: 0.52 € kg^−1^) and organic lettuce (ABAR: 0.70 € kg^−1^, conventional farming: 1.32 € kg^−1^). For peanuts, the pattern is mixed: the ABAR-based system is above the conventional reference in the organic case (0.54 vs. 0.46 € kg^−1^) but slightly below it in the non-organic case (0.47 vs. 0.49 € kg^−1^). Strawberries remain slightly more expensive under the ABAR-based system in both production systems (O: 3.60 vs. 3.55 € kg^−1^; NO: 3.16 vs. 3.08 € kg^-1^).

These comparisons should be interpreted with caution, as both the modeled ABAR TCO and the literature-based reference values depend on assumptions and parameter choices that are subject to uncertainty. The sensitivity analysis addresses this uncertainty only in part for the ABAR model. In addition, cultivation practices, input prices, and yields also differ across regions, and the model would need to be adapted to local conditions to support robust farm-level cost estimates. Even so, the results suggest that ABAR-based systems may offer competitive, and in some crop systems lower production costs, which could support efforts to improve food affordability and food security.

From a life cycle perspective, comparisons with published values are more difficult than for costs because reported GWP estimates span a wide range and often do not distinguish between organic and non-organic production. Moreover, most conventional cradle-to-farm-gate LCAs include harvesting in the system boundary but rarely report harvesting burdens as a separate contribution.

Table 2 therefore reports the ABAR GWP per kilogram of crop for the modeled field operations and complements these values with a literature-based harvesting sensitivity. Specifically, an upper-bound sensitivity case is included in which harvesting is assumed to account for 30% of total cradle-to-farm-gate GWP^44^, enabling a more consistent comparison with conventional cradle-to-farm-gate ranges.

**Table 2:** Comparison of the autonomous battery-powered agricultural robot’s (ABAR’s) global warming potential (GWP) with conventional reference ranges

Type of crop	ABAR GWP (excl. harvesting) [kg CO_2_ eq kg^–1^ crop]	ABAR GWP (estimated total; assuming harvesting = 30% of total cradle-to-farm-gate GWP)^44^ [kg CO_2_ eq kg^−1^ crop]	Conventional GWP (cradle-to-farm-gate range) [kg CO_2_ eq kg^−1^ crop]
Strawberry	Organic: 0.260 Non-Organic: 0.354	Organic: 0.371 Non-Organic: 0.505	0.11 to 5.48^44^
Onion	Organic: 0.028 Non-Organic: 0.036	Organic: 0.039 Non-Organic: 0.051	0.14 to 0.324^69,71,72^
Lettuce	Organic: 0.045 Non-Organic: 0.037	Organic: 0.064 Non-Organic: 0.053	0.08 to 0.58^69,73–75^
Peanut	Organic: 0.294 Non-Organic: 0.269	Organic: 0.420 Non-Organic: 0.384	0.311 to 0.547^69,76^

Due to limited data for organic crops, the reference ranges do not differentiate between organic and non-organic production. ABAR values are reported for the modeled field operations (excluding harvesting) and as estimated total values assuming that harvesting contributes 30% of cradle-to-farm-gate GWP^44^.

Across all four crops, the ABAR results fall within the lower part of the reported conventional cradle-to-farm-gate ranges or, in several cases, substantially below them. For onions and lettuce, both organic and non-organic ABAR values are well below the published ranges, while the strawberry and peanut values lie in the lower segment of the corresponding range. This pattern remains largely unchanged when the ABAR values are converted to estimated cradle-to-farm-gate totals under the assumption that harvesting contributes 30% of total GWP. Part of the remaining gap relative to the reference ranges may reflect uncertainties and simplifications in the model and input data, as discussed above. It may also be influenced by the omission of soil- and fertilizer-related fugitive emissions, such as non-energy emissions from managed soils, which were excluded from the inventory because robust quantification at field scale would require detailed, site-specific soil, climate, and management data that are not available for this analysis, and because these emissions remain methodologically heterogeneous and highly uncertain in agricultural LCA^45,46^. At the same time, the pattern is consistent with structural differences between the systems, as the ABAR is much smaller than typical conventional machinery and relies on battery-electric propulsion rather than predominantly fossil-fuel-powered equipment^47^. Taken together, the results suggest that ABAR-based systems may reduce cradle-to-farm-gate GWP relative to conventional crop production, even under a harvesting-emissions sensitivity, and thereby support progress towards climate-related sustainability targets.

### Maximum coverable area and annual crop yield

The ABAR’s maximum annual coverable area is a key determinant of the robot’s lifetime, the quantity of variable inputs, and the resulting annual crop yield. Because task frequency and timing differ across crops and production systems, the coverable area varies accordingly.

**Fig. 6:**
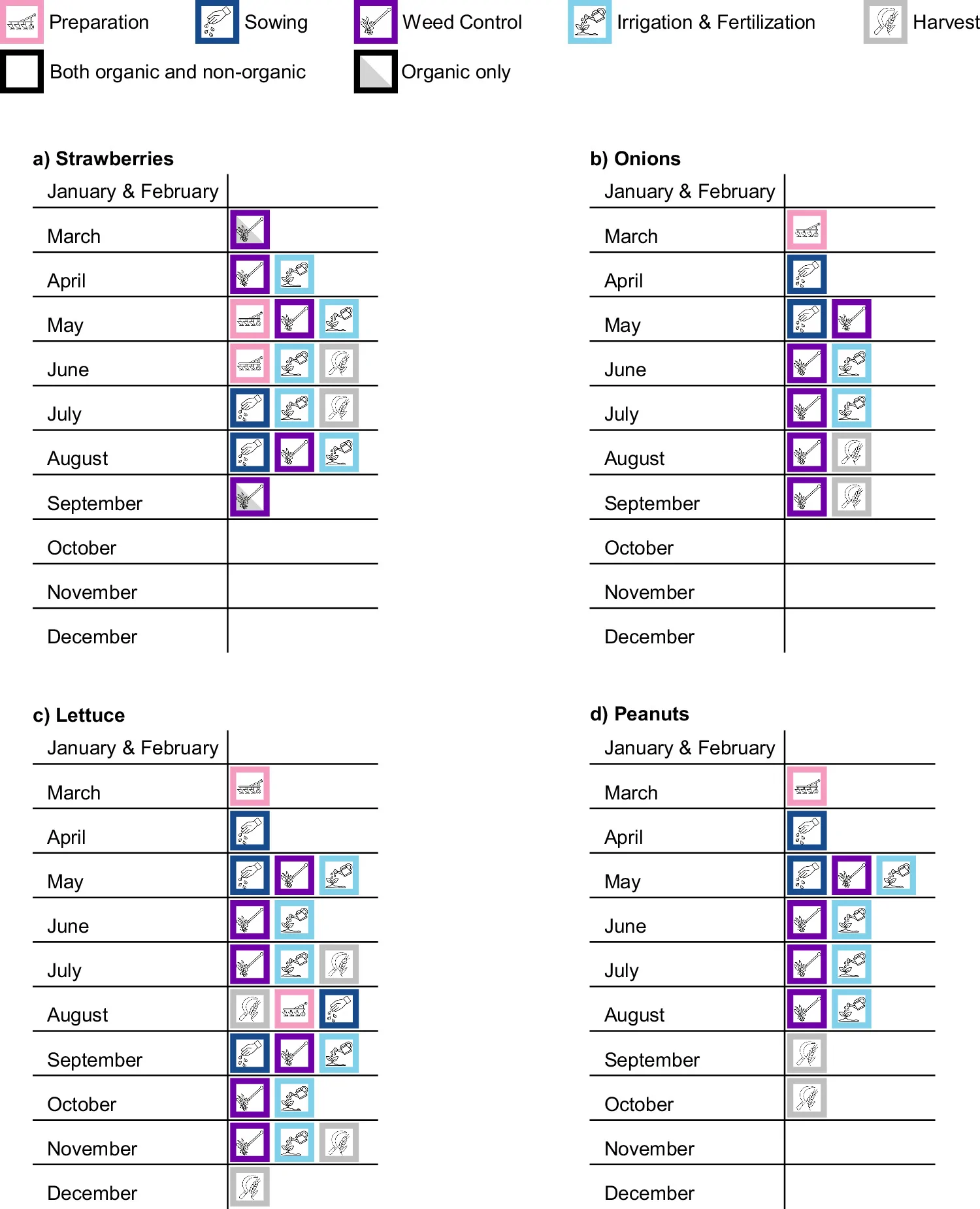
Crop calendars for all modelled crops. This includes (**a**) strawberries, (**b)** onions, (**c)** lettuce and (**d)** peanuts. The calendars indicate which tasks are carried out in each month. Harvesting is shown only because it reduces the number of days available for other tasks.

**Fig. 7:**
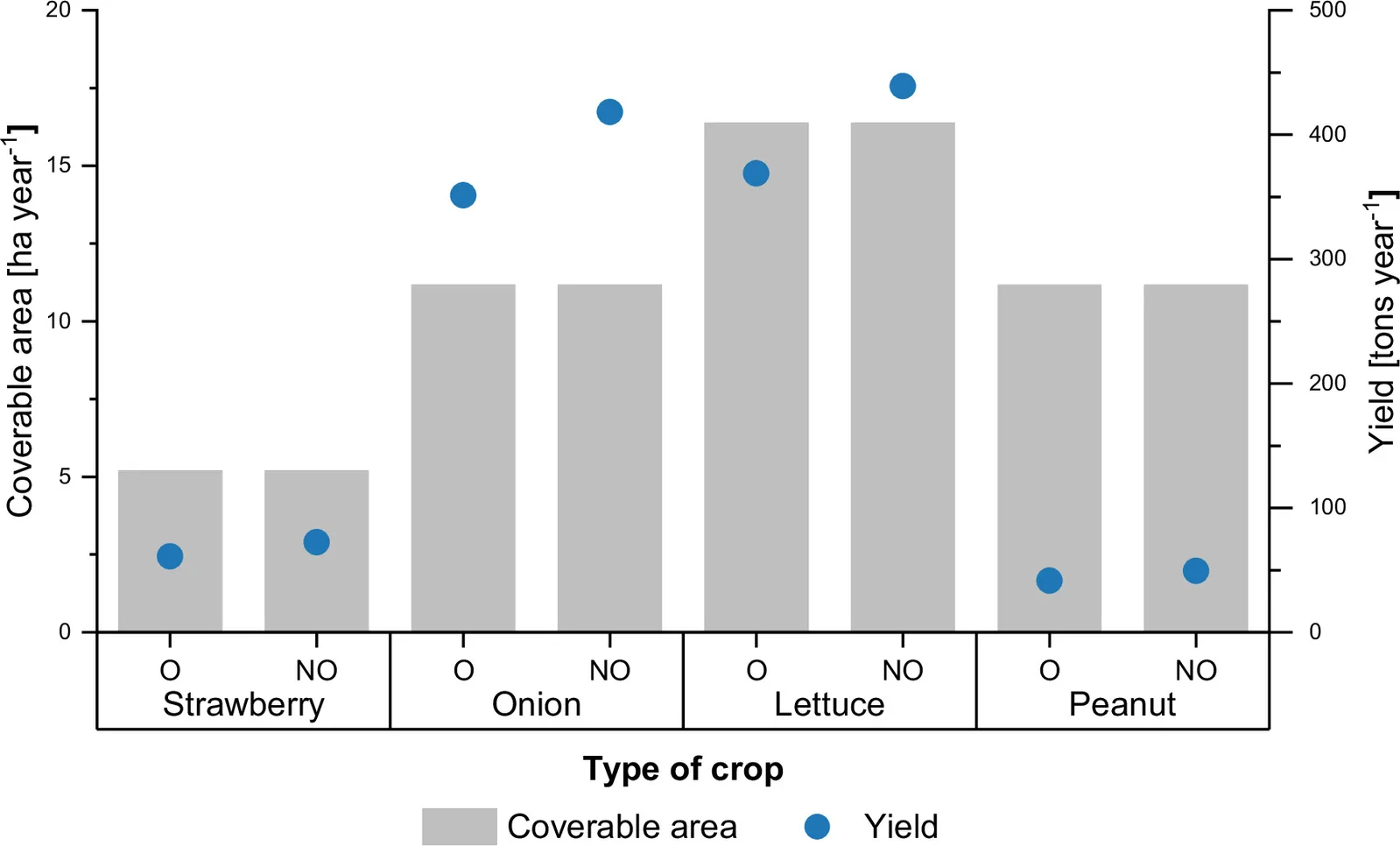
Overview of the robot’s coverable area and crop yield per year. Gray bars represent the maximum coverable area per year while blue dots show the overall yield per crop type and production system (O: Organic, NO: Non-organic).

To capture these differences, crop calendars are used to represent average growing cycles (Fig. 6). For each crop and month, the calendar specifies the task performed. The corresponding monthly task frequencies for organic and non-organic systems are provided in Supplementary Tables 5–13. Combining these calendars with the robot’s working width, the daily operating time, and task-specific speeds yields a crop-specific maximum annual coverable area, reported in hectares in Fig. 7. The corresponding equation is provided in “Methods”.

The resulting coverable areas differ across crops. Strawberries exhibit the lowest annual coverable area, whereas lettuce achieves the highest. These differences arise primarily from the timing and combination of the most time-intensive tasks within the crop calendar. For strawberries, the bottleneck month combines the two most time-consuming operations, soil preparation and irrigation, which reduces the area the ABAR can service. For onions and peanuts, by contrast, the minimum coverable area is determined by the month in which soil preparation alone is the limiting operation, resulting in identical maximum annual coverable areas for both crops. No divergence is observed between organic and non-organic systems because the maximum annual coverable area is determined by the time-limiting operations of soil preparation or irrigation, which occur with the same annual frequency for both systems. Lettuce reaches the highest coverable area because, under the assumed crop calendar, its two growing cycles per year still allow a larger total annual service area than the other modeled crops.

Figure 7 also reports the expected annual yield by crop and production system in tons per year. These values are derived from the maximum coverable area and average yields per hectare for each crop (see Supplementary Table 14)^48,49^. Non-organic lettuce has the highest expected yield, reflecting both a comparatively large coverable area and a high per-hectare yield. Peanuts, by contrast, combine a smaller coverable area with a lower per-hectare yield, which limits the annual output. To reflect expected yield differences between production systems, organic per-hectare yields are assumed to be 16% lower than non-organic yields^50^.

## Discussion

This study combined TCO and LCA to evaluate an ABAR across four crops and two production systems from cradle-to-farm-gate. Under the modeled conditions, ABAR-based production can be cost competitive compared to conventional crop production ranges reported in prior literature while also reducing greenhouse-gas emissions per kilogram of crop, particularly where precision management lowers demand for variable inputs. By spanning strawberries, onions, lettuce, and peanuts in both organic and non-organic systems, the analysis shows that ABAR viability depends strongly on crop-specific task profiles and input requirements and may therefore extend beyond a single niche application within the crop-system contexts considered here. At the same time, this broad crop selection increases the generality of the analysis but reduces the realism and direct comparability of individual scenarios, as each crop requires distinct agronomic and operational assumptions. While these findings are specific to the modeled task profiles, yields, and input intensities rather than crop-universal estimates, transferability is expected to be strongest for systems with similar operational characteristics. This applies to systems where repeated weed control and input management strongly influence costs and emissions. Highly mechanized commodity field-crop systems are not assessed in this single-ABAR analysis. Furthermore, the quantitative results reflect a single compact ABAR parameterization and the resulting maximum annual coverable area; applying the framework to other robot sizes or settings would require re-parameterization, including working width, operating speed, and task calendars.

A central contribution of this study is the joint assessment of economic and environmental performance for a robot that performs multiple tasks over the crop cycle, rather than focusing on individual operations or on cost or environmental outcomes in isolation. Preparation, sowing, weed control, irrigation, and fertilization are represented within a single platform and evaluated consistently across scenarios. This makes it possible to identify where ABAR deployment appears most promising and how task profiles, input requirements, and yields shape trade-offs between TCO and GWP. This scenario-based perspective complements earlier work on agricultural robotics and precision technologies by linking autonomous operation directly to cradle-to-farm-gate performance.

The decomposition into fixed, variable, maintenance, and recycling components, together with the sensitivity analysis, shows that the results are driven more strongly by variable inputs than by the technical parameters of the robotic platform itself. This suggests that, in the modeled scenarios, the main benefits arise less from the platform alone than from more precise management, the potential to reduce labor inputs, and the associated reductions in seed, fertilizer, pesticide, and water use. At the same time, part of the benefit also reflects the replacement of diesel-powered machinery with an electrified platform. Still, crops with very high input requirements remain both costly and emission-intensive even under the modeled ABAR configuration. This pattern further indicates that not all crops are equally suitable for ABAR-based systems and that careful crop and system selection will be important in practice.

Within the set considered here, onions and lettuce emerge as particularly promising applications, especially under organic production where manual weed control is labor-intensive and expensive. These crops combine relatively simple growing cycles with opportunities to reduce weeding and input application, which aligns with the strengths of a compact autonomous platform. At the same time, the limited annual coverable area of a single ABAR suggests that the most immediate opportunities may lie with smaller farms or cooperatives in which a robot is shared. Such applications could support more localized production while helping to alleviate labor shortages in manual field work.

Several limitations should be considered when interpreting these results. The analysis relies on average yield and input data for selected regions and therefore does not capture the full diversity of soils, climates, management practices, and price structures in global agriculture. The robot model is simplified, excludes harvesting processes from the system boundary, and does not fully represent operational aspects such as downtime, failures, or charging logistics in detail. Likewise, liquid-application tasks such as irrigation and chemical weed control are represented through simplified storage and application proxies, and the model does not resolve external supply infrastructure or refill logistics in detail. Consistent with this level of abstraction, traction-dependent operations are represented through draft-force and rolling-resistance calculations, but slip behavior and field stability are not empirically validated. These effects depend on wheel-soil interaction, dynamic load transfer, and field conditions, which fall outside the scope of the present modeling framework. In addition, adoption-related factors, such as access to capital, infrastructure, and farmer acceptance, and broader dynamics such as organizational change, learning effects, and risk are not considered. A further limitation is that the analysis excludes costs associated with the external AI and software infrastructure needed to operate the robot, such as cloud computing, data management, connectivity, and software licensing. These costs were omitted because they are highly deployment-specific and difficult to parameterize consistently across scenarios. While the modeled results suggest that variable crop-production inputs are likely to remain an important cost driver, the potential influence of external AI and software costs cannot be fully assessed without direct modeling and warrants further investigation. The quantitative results should therefore be viewed as an exploration of plausible system-level outcomes rather than as forecasts of actual farm-level performance. This caution is consistent with recent empirical and review-based evidence showing that the realized economic and environmental benefits of precision agriculture are heterogeneous and strongly context dependent, and that practical uptake of field robots remains shaped by factors such as investment cost, reliability, and farm-specific fit^51–54^. Another limitation concerns the comparisons with conventional systems, for which this study benchmarked against literature-based reference ranges rather than direct predictions for specific farming contexts. Future research is, therefore, encouraged to benchmark against data from robots that generate precise, localized predictions in specific farming contexts.

Furthermore, this assessment could be extended through region-specific case studies, explicit modeling of harvesting operations, and integration with field data on input use, reliability, and operator behavior. Future models of small ABARs may also choose to exclude soil preparation from the system boundary, as heavy soil-engaging operations can strongly constrain annual throughput in compact single-ABAR configurations. This step was nevertheless deliberately retained in the present study so that the associated limitation would remain visible. Overall, the findings indicate that ABAR-based production systems may be able to reduce production costs and cradle-to-farm-gate emissions relative to conventional farming practices across the crop-system contexts modeled in this study, particularly where autonomous operation is paired with lower variable-input demand and favorable task profiles.

## Methods

We apply an integrated TCO and LCA framework to model an autonomous battery-powered agricultural robot performing preparation, sowing, mechanical and chemical weed control, irrigation, and fertilization. The analysis adopts a cradle-to-farm-gate system boundary and excludes harvesting (see Fig. 1), which falls outside the scope of the modeled platform. A schematic overview of the model architecture is provided in Fig. 2. Parameter values are listed in Supplementary Tables 15–21.

### Coverable area per year

To support the TCO and LCA calculations, the ABAR’s maximum annual coverable area for each crop, *A*_Crop_ is first determined. Because crop calendars differ in both task frequency and timing, the coverable area is crop-specific. Using daily operating hours *h*, days per month *d*, task frequency per month and crop *x*_s, crop_ for each task s (preparation, sowing, mechanical or chemical weed control, irrigation, and fertilization), tool width *W*_Tool_, and task-specific working speed *v*_s_, *A*_Crop_ can be calculated (see Eq. (1)). Here, *h* already accounts for non-working time (e.g., charging). If harvesting occurs in a given month, *d* is reduced by 7. Because *A*_Crop_ is calculated on a month-by-month basis, the maximum annual coverable area is constrained by the month with the smallest coverable area across the crop calendar.

1 $${A}_{{{{\rm{Crop}}}}}=\frac{h\cdot d\cdot 3600}{{\sum }_{{{{\rm{s}}}}=1}^{{{{\rm{n}}}}}\frac{{x}_{{{{\rm{s}}}},{{{\rm{crop}}}}}}{{W}_{{{{\rm{Tool}}}}}\cdot {v}_{{{{\rm{s}}}}}}}$$

The monthly crop calendar derived from the literature is summarized in Fig. 6. Additional information regarding task frequencies *x*_s, crop_ and the derivation of Eq. (1) is provided in Supplementary Note 1; the crop calendar is provided in Supplementary Tables 5–13.

### Total cost of ownership analysis

The TCO analysis applies a high-level process-based cost model, consistent with prior work^55–57^. In this approach, costs are built from technical inputs, decomposing a complex and underexplored system into smaller cost elements. This makes it possible to draw on established technical data where real world data are limited and improves transparency by identifying the parameters that most strongly influence total costs^58^.

The total costs of the ABAR are levelized to cost per kg of crop to align the functional unit with that used in the LCA. As shown in Eq. (2), the levelized cost per kilogram of crop comprises annualized capital expenditures, crop-specific operational expenditures, and annualized end-of-life recycling costs. Maintenance is modeled as a fixed percentage of CAPEX, following prior studies^55,56^. The sum of these components is divided by the expected annual yield of each crop (Eq. (3)), which is based on average annual yields in the EU and US^48,49^ (see Supplementary Table 14 for further details).

2 $${{{\rm{LCPK}}}}_{{{\rm{Crop}}}}=\frac{{{{{\rm{CAPEX}}}}}_{{{{\rm{Annualized}}}},{{{\rm{crop}}}}}\,(1+{{{\rm{Maintenance}}}})+{{{\rm{OPEX}}}}_{{{\rm{Crop}}}}+{{{\rm{Recycling}}}}_{{{{\rm{Annualized}}}},{{{\rm{crop}}}}}}{{{{{\rm{kg}}}}\; {{{\rm{per}}}}\; {{{\rm{year}}}}}_{{{{\rm{Crop}}}}}}$$

3 $${{{\rm{kg}}}}\; {{{\rm{per}}}}\; {{{\rm{year}}}}_{{{\rm{Crop}}}}={{{\rm{average}}}}\; {{{\rm{yield}}}}\; {{{\rm{per}}}}\; {{{\rm{hectare}}}}_{{{\rm{Crop}}}}\cdot {A}_{{{\rm{Crop}}}}$$

This study includes the battery, drivetrain (electric motor and wheels), sensors, chassis, ballast, and tools as CAPEX components. Annualized CAPEX (see Eq. (4)) is therefore calculated as the sum of component costs multiplied by the capital recovery factor (crf., Eq. (5)).

4 $${{{\rm{CAPEX}}}}_{{{\rm{Annualized}}}},_{{{\rm{crop}}}}={{{\rm{crf}}}}_{{{{\rm{Robot}}}},{{{\rm{crop}}}}} \cdot ({c}_{{{\rm{Battery}}}}+{c}_{{{\rm{Drivetrain}}}}+{c}_{{{\rm{Sensors}}}}+{c}_{{{\rm{Chassis}}}},_{{{{\rm{ballast}}}},{{{\rm{and}}}}\;{{{\rm{tools}}}}})$$

5 $${{{{\rm{crf}}}}}_{{{{\rm{Robot}}}},{{{\rm{crop}}}}}=\frac{i{(1+i)}^{{{{{\rm{Lifespan}}}}}_{{{{\rm{Robot}}}},{{{\rm{crop}}}}}}}{{(1+i)}^{{{{{\rm{Lifespan}}}}}_{{{{\rm{Robot}}}},{{{\rm{crop}}}}}}-1}$$

Following prior literature^22,24^, the lifespan of the robot is set to 5,000 h. Using *A*_Crop_ and rearranging Eq. (1), the annual working time of the robot *t*_Total,crop_ is obtained (Eq. (6)), from which the lifetime in years for each crop, Lifespan_Robot, crop_, follows. The interest rate *i* is set to 0.05, following prior studies^55,56^.

6 $${t}_{{{{\rm{Total}}}},{{{\rm{crop}}}}}={\sum }_{{{{\rm{s}}}}=1}^{n}\frac{{A}_{{{{\rm{Crop}}}}}\cdot {x}_{{{{\rm{s}}}},{{{\rm{crop}}}}}}{{W}_{{{{\rm{Tool}}}}}\cdot {v}_{{{{\rm{s}}}}}}$$

The costs for the CAPEX components *c*_c_ are determined by utilizing the following generic relation (Eq. (7)):

7 $${c}_{{{{\rm{c}}}}}={q}_{{{{\rm{c}}}}}\cdot {p}_{{{{\rm{c}}}}}$$

where *q*_c_ is the required quantity (capacity, mass, count, or rated power) and *p*_c_ the specific unit cost. The specific equations for each component can be found in Supplementary Note 2.

OPEX comprises the crop-specific costs of electricity, seeds, pesticides, water, fertilizers and labor (Eq. (8)).

8 $${{{{\rm{OPEX}}}}}_{{{{\rm{Crop}}}}}={c}_{{{{\rm{Electricity}}}},{{{\rm{crop}}}}}+{{c}_{{{{\rm{Seeds}}}},{{{\rm{crop}}}}}+c}_{{{{\rm{Pesticide}}}},{{{\rm{crop}}}}}+{c}_{{{{\rm{Water}}}},{{{\rm{crop}}}}}+{c}_{{{{\rm{Fertilizer}}}},{{{\rm{crop}}}}}+{c}_{{{{\rm{Labor}}}},{{{\rm{crop}}}}}$$

Costs for seeds, pesticides, and fertilizers (Eqs. (9)-(11)) are calculated from the crop’s annual coverable area *A*_Crop_ and crop-specific unit costs per area. Reflecting the precision-application capability of autonomous robots, pesticide and fertilizer use is assumed to be reduced by 50% relative to conventional practice, consistent with results reported in the literature^13,59^. Irrigation water costs (Eq. (12)) are computed in a similar way, with the additional parameter *V*_Water, crop_/ha representing the amount of water needed per hectare. As for fertilizer and pesticide use, water use is assumed to be 50% lower than under conventional practice, placing this assumption within the range of savings reported in the literature^60,61^.

9 $${c}_{{{{\rm{Seeds}}}},{{{\rm{crop}}}}}={A}_{{{{\rm{Crop}}}}}{\cdot c}_{{{{\rm{Seeds}}}},{{{\rm{specific}}}}}$$

10 $${c}_{{{{\rm{Pesticide}}}},{{{\rm{crop}}}}}={0.5\cdot A}_{{{{\rm{Crop}}}}}{\cdot c}_{{{{\rm{Pesticide}}}},{{{\rm{specific}}}}}$$

11 $${c}_{{{{\rm{Fertilizer}}}},{{{\rm{crop}}}}}={0.5\cdot A}_{{{{\rm{Crop}}}}}{\cdot c}_{{{{\rm{Fertilizer}}}},{{{\rm{specific}}}}}$$

12 $${c}_{{{{\rm{Water}}}},{{{\rm{crop}}}}}=0.5\cdot {A}_{{{{\rm{Crop}}}}}\cdot \frac{{V}_{{{{\rm{Water}}}},{{{\rm{crop}}}}}}{{{{\rm{ha}}}}}\cdot {c}_{{{{\rm{Water}}}},{{{\rm{specific}}}}}$$

Labor costs (Eq. (13)) are calculated from the robot’s crop-specific annual working time *t*_Total, crop_, the hourly labor cost *c*_Labor, specific_, and a supervision factor. Although the ABAR is modeled as autonomous during field operations, labor is not assumed to be zero because operation still requires setup, supervision, occasional intervention, and logistical support^24,31^. In the base case, the supervision factor is set to 15% of crop-specific annual robot working time^62^.

13 $${c}_{{{{\rm{Labor}}}}}={{t}_{{{{\rm{Total}}}},{{{\rm{crop}}}}}\cdot c}_{{{{\rm{Labor}}}},{{{\rm{specific}}}}}\cdot {{{\rm{supervision\; factor}}}}$$

Electricity cost estimation is based on the required tractive effort, consisting of draft force *F*_Draft_ and rolling resistance *F*_Rolling resistance_. Draft force for task s is given by Eq. (14):

14 $${F}_{{{{\rm{Draft}}}},{{{\rm{s}}}}}={S}_{{{{\rm{Tool}}}}}\cdot {D}_{{{{\rm{s}}}}}\cdot \left(k+d\cdot {v}_{{{{\rm{s}}}}}+e\cdot {{v}_{{{{\rm{s}}}}}}^{2}\right)$$

where *D*_s_ is working depth, *S*_Tool_ is the tool size parameter as defined in the American Society of Agricultural and Biological Engineers (ASAE) formulation and is expressed either as tool width (*W*_Tool_) or as number of rows depending on the tool category, *v*_s_ is working speed, and *k*, *d*, *e* are tool-specific coefficients from the Goryachkin equation^63^ parameterized using the ASAE D497.7 standard^64^.

Rolling resistance is computed by Eq. (15):

15 $${F}_{{{{\rm{Rolling}}}}\,{r}{{{\rm{esistance}}}},{{{\rm{s}}}}}={m}_{{{{\rm{s}}}}}\cdot g\cdot r$$

where *m*_Robot and tools_,_s_ is the mass of the robot plus the task-specific tool, *g* is gravitational acceleration, and *r* is the rolling resistance coefficient.

Power demand is obtained by multiplying tractive forces by working speed (Eq. (16)).

16 $${{{{\rm{Power}}}}}_{{{{\rm{Electric}}}}\; {{{\rm{Motor}}}},{{{\rm{s}}}}}=({F}_{{{{\rm{Draft}}}},s}+{F}_{{{{\rm{Rolling}}}}{{{\rm{resistance}}}},s})\cdot {v}_{{{{\rm{s}}}}}$$

Electricity consumption per hectare and task (Eq. (18)) follows by dividing the required power by the processed area rate $\dot{A}$ (Eq. (17))—the product of tool width *W*_Tool_ and speed—and by the overall drivetrain efficiency ${\prod }_{{{{\rm{c}}}}=1}^{{{{\rm{n}}}}}{\eta }_{{{{\rm{c}}}}}$(battery, electric motor):

17 $$\dot{A}\,=\frac{36}{100}\,{W}_{{{{\rm{Tool}}}}}\cdot {v}_{{{{\rm{s}}}}}$$

18 $${{{{\rm{Electricity}}}}\; {{{\rm{consumption}}}}\; {{{\rm{per}}}}\; {{{\rm{hectare}}}}}_{{{{\rm{s}}}}}=\,\frac{({F}_{{{{\rm{Draft}}}},{{{\rm{s}}}}}+{F}_{{{{\rm{Rolling}}}}\; {{{\rm{resistance}}}},{{{\rm{s}}}}})\cdot {v}_{{{{\rm{s}}}}}}{\dot{A}}\cdot \frac{1}{{\prod }_{{{{\rm{c}}}}=1}^{{{{\rm{n}}}}}{\eta }_{{{{\rm{c}}}}}}\, \\=\,\frac{({S}_{{{{\rm{Tool}}}}}\cdot {D}_{{{{\rm{s}}}}}\cdot \left(k+d \cdot {v}_{{{{\rm{s}}}}}+e\cdot {{v}_{{{{\rm{s}}}}}}^{2}\right)+{m}_{{{{\rm{s}}}}}\cdot g \cdot r)\cdot {v}_{{{{\rm{s}}}}}}{\dot{A}}\cdot \frac{1}{{\prod }_{{{{\rm{c}}}}=1}^{{{{\rm{n}}}}}{\eta }_{{{{\rm{c}}}}}}$$

Multiplying electricity consumption per hectare and task by the total worked hectares per year and task (Eq. (19)) yields the total electricity consumption per year and task (Eq. (20)). Summing across all tasks then gives the crop’s total annual electricity consumption (Eq. (21)).

19 $${{{{\rm{hectares}}}}\; {{{\rm{per}}}}\; {{{\rm{year}}}}}_{{{{\rm{s}}}},{{{\rm{crop}}}}}={A}_{{{{\rm{Crop}}}}}\cdot {\sum }_{{{{\rm{month}}}}={{{\rm{January}}}}}^{{{{\rm{December}}}}}{x}_{{{{\rm{s}}}},{{{\rm{month}}}}}$$

20 $${{{{\rm{Electricity}}}}\; {{{\rm{consumption}}}}\; {{{\rm{per}}}}\; {{{\rm{year}}}}}_{{{{\rm{s}}}},{{{\rm{crop}}}}}={{{{\rm{Electricity}}}}\; {{{\rm{consumption}}}}\; {{{\rm{per}}}}\; {{{\rm{hectare}}}}}_{{{{\rm{s}}}}}\\ {\cdot {{{\rm{hectares}}}}\; {{{\rm{per}}}}\; {{{\rm{year}}}}}_{{{{\rm{s}}}},{{{\rm{crop}}}}}$$

21 $${{{{\rm{Total}}}}\; {{{\rm{electricity}}}}\; {{{\rm{consumption}}}}\; {{{\rm{per}}}}\; {{{\rm{year}}}}}_{{{{\rm{Crop}}}}}\,=\,{\sum }_{{{{\rm{s}}}}=1}^{{{{\rm{n}}}}}{{{{\rm{Electricity}}}}\; {{{\rm{consumption}}}}\; {{{\rm{per}}}}\; {{{\rm{year}}}}}_{{{{\rm{s}}}},{{{\rm{crop}}}}}\\ ({{{\rm{with}}}}\,{{{\rm{s}}}}={{{\rm{preparation}}}},{{{\rm{sowing}}}},{{{\rm{weed\; control}}}},{{{\rm{watering}}}},{{{\rm{or}}}}\; {{{\rm{irrigation}}}})$$

Multiplying this value by the specific unit electricity price then yields the annual electricity cost (Eq. (22)).

22 $${c}_{{{\rm{Electricity}}}},{{{\rm{crop}}}}={{{\rm{Total}}}}\; {{{\rm{electricity}}}}\; {{{\rm{consumption}}}}\; {{{\rm{per}}}}\; {{{\rm{year}}}}_{{{\rm{Crop}}}}\cdot {c}_{{{\rm{Electricity}}}},{{{\rm{specific}}}}$$

Annualized recycling cost (Eq. (23)), the final cost component of Eq. (2), is calculated by multiplying the quantity of each end-of-life material *q*_*c*, recycling_ (battery, steel, aluminum, rubber, and linear low-density polyethylene (LLDPE)) by its unit recycling cost *c*_c, recycling_, summing across materials, and dividing by the crop-specific robot lifetime.

23 $${{{\rm{Recycling}}}}_{{{\rm{Annualized}}}},_{{{\rm{crop}}}}=\frac{1}{{{\rm{Lifespan}}}}_{{{{\rm{Robot}}}},{{{\rm{crop}}}}},\cdot {\sum }_{{{{\rm{c}}}}=1}^{{{\rm{n}}}}{q}_{{{{\rm{c}}}},_{{{\rm{recycling}}}}} \cdot {c}_{{{{\rm{c}}}},_{{{\rm{recycling}}}}}$$

### Life cycle assessment

The LCA conducted in this study follows the standardized International Organization for Standardization (ISO) 14040/14044 framework^65,66^ comprising four elements: (i) goal and scope definition, (ii) life cycle inventory (LCI), (iii) life cycle impact assessment (LCIA), and (iv) interpretation. The following paragraphs address these steps in turn. As noted above, LCA equations are aligned to those used in the TCO analysis.

The goal of this LCA is to quantify the environmental impacts of an ABAR across manufacturing, operation, and end-of-life, and to attribute those impacts to crop-specific scenarios. The assessment applies a cradle-to-farm-gate system boundary, with harvesting excluded, and follows an attributional cut-off approach that tracks direct flows of materials, energy, and emissions over the ABAR’s lifetime^67,68^. The functional unit is 1 kg of crop produced.

Project data within the cradle-to-farm-gate boundary, covering raw materials, energy, and waste flows for the ABAR and variable inputs (e.g., fertilizers, seed), are compiled in the LCI. These data were obtained from academic literature and ecoinvent 3.11 via ecoQuery^68,69^. Component-specific LCIs are provided in Supplementary Tables 22–35.

Using the Environmental Footprint (EF) 3.1 LCIA method^70^, the LCI is translated into 16 impact categories. This study focuses on global warming potential 100 because of its relevance^33–35^. GWP is determined by aggregating greenhouse-gas emissions (e.g., from CO₂, methane, or nitrous oxide) into CO₂-equivalents over a 100-year horizon. Complete results for all impact categories are reported in Supplementary Tables 3 and 4. Although the following equations are only shown for GWP, they were applied analogously to all 16 impact categories.

GWP per kilogram of crop (Eq. (24)) is obtained by summing fixed GWP, variable GWP, and recycling GWP, again including a fixed maintenance factor, and dividing the total by the crop’s expected annual yield.

24 $${{{\rm{GWP}}}}\; {{{\rm{per}}}}\; {{{\rm{kg}}}}\; {{{\rm{crop}}}}=\frac{{{{{\rm{GWP}}}}}_{{{{\rm{Fixed}}}},{{{\rm{crop}}}}}\left(1+{{{\rm{Maintenance}}}}\right)+{{{{\rm{GWP}}}}}_{{{{\rm{Variable}}}},{{{\rm{crop}}}}}+{{{{\rm{GWP}}}}}_{{{{\rm{Recycling}}}},{{{\rm{crop}}}}}\,}{{{{{\rm{kg}}}}\; {{{\rm{per}}}}\; {{{\rm{year}}}}}_{{{{\rm{Crop}}}}}}$$

Fixed GWP (Eq. (25)) is determined by multiplying the sum of the fixed component impacts by the impact recovery factor (Eq. (26)), defined as the reciprocal of the robot’s lifetime.

25 $${{{\rm{GWP}}}}_{{{{\rm{Fixed}}}},{{{\rm{crop}}}}}={{{\rm{irf}}}}_{{{{\rm{Robot}}}},{{{\rm{crop}}}}} \cdot ({{{\rm{GWP}}}}_{{{\rm{Battery}}}}+{{{\rm{GWP}}}}_{{{\rm{Drivetrain}}}} \\+{{{\rm{GWP}}}}_{{{\rm{Sensors}}}}+{{{\rm{GWP}}}}_{{{{\rm{Chassis}}}},{{{\rm{ballast}}}}\;{{{\rm{and}}}}\;{{{\rm{tools}}}}})$$

26 $${{{{\rm{impact}}}}\; {{{\rm{recovery}}}}\; {{{\rm{factor}}}}\left({{{\rm{irf}}}}\right)}_{{{{\rm{Robot}}}},{{{\rm{crop}}}}}=\frac{1}{{{{{\rm{Lifespan}}}}}_{{{{\rm{Robot}}}},{{{\rm{crop}}}}}}$$

The individual fixed component impacts GWP_c_ are calculated with a generic relation (Eq. (27)):

27 $${{{{\rm{GWP}}}}}_{{{{\rm{c}}}}}={\sum }_{{{{\rm{k}}}}=1}^{{{{{\rm{n}}}}}_{{{{\rm{c}}}}}}{q}_{{{{\rm{c}}}},{{{\rm{k}}}}}\cdot {{{{\rm{unit}}}}\_{{{\rm{GWP}}}}}_{{{{\rm{c}}}},{{{\rm{k}}}}}$$

where *q*_c,k_ is the required quantity for sub-item k of component c (capacity or mass) and unit_GWP_c_ is the unit impact factor from the EF 3.1 LCIA using ecoinvent 3.11 data. Unit factors are included for batteries, electric motors, rubber (wheels), steel and steel metalworking, sensors, aluminum and aluminum metalworking, LLDPE, and blow molding for LLDPE. Component-specific equations are provided in Supplementary Note 2.

Variable GWP (Eq. (28)) is the sum of impacts from electricity, seed, pesticides, water, and fertilizers.

28 $${{{{\rm{GWP}}}}}_{{{{\rm{Variable}}}},{{{\rm{crop}}}}}\,={{{{\rm{GWP}}}}}_{{{{\rm{Electricity}}}},{{{\rm{crop}}}}}+{{{{\rm{GWP}}}}}_{{{{\rm{Seeds}}}},{{{\rm{crop}}}}} \\+{{{{\rm{GWP}}}}}_{{{{\rm{Pesticides}}}},{{{\rm{crop}}}}}+{{{{\rm{GWP}}}}}_{{{{\rm{Water}}}},{{{\rm{crop}}}}}+{{{{\rm{GWP}}}}}_{{{{\rm{Fertilizer}}}},{{{\rm{crop}}}}}$$

GWP_Electricity, crop_ (Eq. (29)) is the product of total yearly electricity consumption and unit impact of electricity.

29 $${{{{\rm{GWP}}}}}_{{{{\rm{Electricity}}}},{{{\rm{crop}}}}}\,={{{{\rm{Total}}}}\; {{{\rm{electricity}}}}\; {{{\rm{consumption}}}}\; {{{\rm{per}}}}\; {{{\rm{year}}}}}_{{{{\rm{Crop}}}}}\cdot {{{{\rm{unit}}}}\_{{{\rm{GWP}}}}}_{{{{\rm{Electricity}}}}}$$

GWP_Seeds, crop_ (Eq. (30)) is determined by multiplying the crop-specific quantity of seeds per hectare *q*_Seeds, crop_ (mass or amount) by *A*_Crop_ and the unit impact of crop-specific seeds.

30 $${{{{\rm{GWP}}}}}_{{{{\rm{Seeds}}}}}\,={q}_{{{{\rm{Seeds}}}},{{{\rm{crop}}}}}\cdot {{A}_{{{{\rm{Crop}}}}}\cdot {{{\rm{unit}}}}\_{{{\rm{GWP}}}}}_{{{{\rm{Seeds}}}},{{{\rm{crop}}}}}$$

GWP_Pesticides, crop_ (Eq. (31)) is calculated as the product of the crop-specific pesticide mass *m*_Pesticide, crop_, *A*_Crop_, and the unit impact of pesticides, multiplied by 0.5 to reflect the assumed reduction from ABAR precision application.

31 $${{{{\rm{GWP}}}}}_{{{{\rm{Pesticide}}}},{{{\rm{crop}}}}}\,={0.5\cdot m}_{{{{\rm{Pesticide}}}},{{{\rm{crop}}}}}\cdot {{A}_{{{{\rm{Crop}}}}}\cdot {{{\rm{unit}}}}\_{{{\rm{GWP}}}}}_{{{{\rm{Pesticide}}}}}$$

GWP_Water, crop_ (Eq. (32)) is the product of crop-specific water mass *m*_Water, crop_, *A*_Crop_, and the unit impact of water, multiplied by 0.5 to reflect the ABAR’s reduced water use.

32 $${{{{\rm{GWP}}}}}_{{{{\rm{Water}}}},{{{\rm{crop}}}}}\,=0.5\cdot {m}_{{{{\rm{Water}}}},{{{\rm{crop}}}}}\cdot {A}_{{{{\rm{Crop}}}}}\cdot {{{{\rm{unit}}}}\_{{{\rm{GWP}}}}}_{{{{\rm{Water}}}}}$$

GWP_Fertilizer, crop_ (Eq. (33)) is calculated as 0.5 ∙ *A*_Crop_ times the sum of nutrient-specific impacts, i.e., the per-hectare application masses of N, P_2_O_5_, and K_2_O for the crop multiplied by their respective unit impact factors. The factor 0.5 reflects the assumed reduction in fertilizer use enabled by ABAR precision application.

33 $${{{{\rm{GWP}}}}}_{{{{\rm{Fertilizer}}}},{{{\rm{crop}}}}}\,=0.5 \cdot {A}_{{{{\rm{Crop}}}}}\cdot ({m}_{{{{\rm{Fertilizer}}}},{{{\rm{N}}}},{{{\rm{crop}}}}}\,{\cdot \,{{{\rm{unit}}}}\_{{{\rm{GWP}}}}}_{{{{\rm{Fertilizer}}}}\,{{{\rm{N}}}}} \\+{m}_{{{{\rm{Fertilizer}}}},{{{{\rm{P}}}}}_{2}{{{{\rm{O}}}}}_{5},{{{\rm{crop}}}}}\,{\cdot \,{{{\rm{unit}}}}\_{{{\rm{GWP}}}}}_{{{{\rm{Fertilizer}}}}\,{{{{\rm{P}}}}}_{2}{{{{\rm{O}}}}}_{5}} \\+{m}_{{{{\rm{Fertilizer}}}},{{{{\rm{K}}}}}_{2}{{{\rm{O}}}},{{{\rm{crop}}}}}\,{\cdot \,{{{\rm{unit}}}}\_{{{\rm{GWP}}}}}_{{{{\rm{Fertilizer}}}}\,{{{{\rm{K}}}}}_{2}{{{\rm{O}}}}})$$

Finally, GWP_Recycling, crop_ (Eq. (34)) is obtained by multiplying irf_Robot, crop_ by the sum over end-of-life materials of mass *m*_c, end-of-life_ (battery, steel, aluminum, rubber, LLDPE) times the unit impact.

34 $${{GWP}}_{{{{\rm{Recycling}}}},{{{\rm{crop}}}}}={{irf}}_{{{{\rm{Robot}}}},{{{\rm{crop}}}}}\cdot {\sum }_{c=1}^{n}{m}_{c,{{{\rm{end}}}}-{{{\rm{of}}}}-{{{\rm{life}}}}}\cdot {{unit}}\_{{GWP}}_{c,{{{\rm{recycling}}}}}$$

### Sensitivity analysis

Sensitivity analyses were conducted across all crops for both TCO and LCA to address uncertainty associated with the large number of input parameters. To keep the analysis tractable, conceptually related parameters were grouped into scenarios and varied simultaneously rather than individually. For each scenario, the absolute deviation from the base case was first calculated for each crop and then converted into a percentage deviation. These percentage deviations were subsequently averaged across all crops to obtain the scenario-level average percentage deviation from the base case. Table 1 summarizes the parameter groupings and variation ranges applied in each scenario.

## Supplementary information

Supplementary Information

Transparent Peer Review file

## Source data

Source Data

## Data Availability

The data generated in this study are provided in the Supplementary Information and Source Data file. The LCI data generated in this study are provided in the Supplementary Information. The ecoinvent data used in this study are available under restricted access due to licensing requirements; access can be obtained with a valid ecoinvent license. The data used for the cost analysis in this study are provided in the Supplementary Information. The Source Data underlying the bar charts generated in this study are provided in the Source Data file. Source data are provided with this paper.
