# Supplementary material for: Techno-economic and environmental assessment of next-generation battery-powered farming robots
Source: Nat Commun. 2026 Nov 2;17:11336. doi: 10.1038/s41467-026-78281-7 (PMC13728115; doi:10.1038/s41467-026-78281-7)
Supplement: Supplementary file 1 [file 41467_2026_78281_MOESM1_ESM.pdf]

Supplementary Information

Techno-economic and environmental assessment of next-generation battery-powered farming robots

Simon Schlehuber[1,*], Moritz Gutsch[1], Luca Stegemann[1], Thomas Hendrickson[2], Stephan von Delft[1,3]

[1] Institute of Business Chemistry, University of Münster, Leonardo-Campus 1, 48149 Münster, Germany

[2]University of California, Davis, Civil & Environmental Engineering, One Shields Avenue, Davis, CA95616, United States

[3]University of Glasgow, Adam Smith Business School, Glasgow G11 6EY, United Kingdom

*Corresponding author. E-mail address: simon.schlehuber@uni-muenster.de

Contents

Supplementary Note 1 3
Supplementary Note 2 7
Supplementary Table 1 9
Supplementary Table 2 10
Supplementary Table 3 11
Supplementary Table 4 12
Supplementary Table 5 13
Supplementary Table 6 13
Supplementary Table 7 14
Supplementary Table 8 14
Supplementary Table 9 15
Supplementary Table 10 15
Supplementary Table 11 16
Supplementary Table 12 16
Supplementary Table 13 17
Supplementary Table 14 17
Supplementary Table 15 18
Supplementary Table 16 19
Supplementary Table 17 19
Supplementary Table 18 19
Supplementary Table 19 20
Supplementary Table 20 20
Supplementary Table 21 21
Supplementary Table 22 21
Supplementary Table 23 21
Supplementary Table 24 22
Supplementary Table 25 22
Supplementary Table 26 22
Supplementary Table 27 23
Supplementary Table 28 23
Supplementary Table 29 23
Supplementary Table 30 23
Supplementary Table 31 23
Supplementary Table 32 24
Supplementary Table 33 24
Supplementary Table 34 24
Supplementary Table 35 25
Supplementary References 26

Supplementary Note 1

To derive the general form of Eq. (1) in the main manuscript, which is used to determine the addressable area A_{Crop}, the following parameters are required: the tool width W_{Tool}, the robot speed v_s for each specific task s, the monthly frequency of task s given by x_s, the working hours per day h, and the working days per month d. If harvesting occurs in a given month, d is reduced by 7 working days. Using these parameters, derivations for months with one, two, and three concurrent tasks can be carried out, which together lead to the general form of the equation.

Derivation #1: Calculation of A_{Crop} for **1** action only

1) Time required for exactly one task s per month: t_1 (supplementary equation (1))

$$t_1 = \frac{A_{\text{Crop}} \cdot x_1}{W_{\text{Tool}} \cdot v_1} \tag{1}$$

2) Total time required for all tasks (in this case 1) in an area A_{Crop} per month: t_{Total} (supplementary equation (2))

$$t_{\text{total}} = t_1 = \frac{A_{\text{Crop}} \cdot x_1}{W_{\text{Tool}} \cdot v_1} \tag{2}$$

3) Total available working time per month: $t_{\text{available}}$ (supplementary equation (3))

$$t_{\text{available}} = h \cdot d = t_{\text{total}} \tag{3}$$

4) Equate t_{total} and $t_{\text{available}}$ using the term from supplementary equation (2) because they are equal (supplementary equation (4)):

$$\frac{A_{\text{Crop}} \cdot x_1}{W_{\text{Tool}} \cdot v_1} = t_{\text{available}} \tag{4}$$

5) Solve supplementary equation (4) for Area A_{Crop} (supplementary equation (5)):

$$A_{\text{Crop}} = \frac{t_{\text{available}}}{\frac{x_1}{W_{\text{Tool}} \cdot v_1}} <=> A_{\text{Crop}} = \frac{h \cdot d}{\frac{x_1}{W_{\text{Tool}} \cdot v_1}} \tag{5}$$

Derivation #2: Calculation of A_{Crop} for **2** actions

1) Time required for two tasks *s* per month: t_1 and t_2 (supplementary equation (6) and supplementary equation (7))

$$t_1 = \frac{A_{\text{Crop}} \cdot x_1}{W_{\text{Tool}} \cdot v_1} \tag{6}$$

$$t_2 = \frac{A_{\text{Crop}} \cdot x_2}{W_{\text{Tool}} \cdot v_2} \tag{7}$$

2) Total time required for all tasks (in this case 2) in an area A_{Crop} per month: t_{Total} (supplementary equation (8))

$$t_{\text{total}} = t_1 + t_2 = \frac{A_{\text{Crop}} \cdot x_1}{W_{\text{Tool}} \cdot v_1} + \frac{A_{\text{Crop}} \cdot x_2}{W_{\text{Tool}} \cdot v_2} \tag{8}$$

3) Total available working time per month: $t_{available}$ (supplementary equation (9))

$$t_{\text{available}} = h \cdot d = t_{\text{total}} \tag{9}$$

4) Equate t_{total} and $t_{available}$ using the term from supplementary equation (9) because they are equal (supplementary equation (10)):

$$\frac{A_{\text{Crop}} \cdot x_1}{W_{\text{Tool}} \cdot v_1} + \frac{A_{\text{Crop}} \cdot x_2}{W_{\text{Tool}} \cdot v_2} = t_{\text{available}} \tag{10}$$

5) Solve supplementary equation (10) for Area A_{Crop} (supplementary equation (11)):

$$A_{\text{Crop}} \left(\frac{x_1}{W_{\text{Tool}} \cdot v_1} + \frac{x_2}{W_{\text{Tool}} \cdot v_2} \right) = t_{\text{available}} <=> A_{\text{Crop}} = \frac{h \cdot d}{\frac{x_1}{W_{\text{Tool}} \cdot v_1} + \frac{x_2}{W_{\text{Tool}} \cdot v_2}} \tag{11}$$

Derivation #3: Calculation of Area A_{Crop} for **3** actions

1) Time required for three tasks *s* per month: t_1, t_2 and t_3 (supplementary equation (12), supplementary equation (13), and supplementary equation (14))

$$t_1 = \frac{A_{\text{Crop}} \cdot x_1}{W_{\text{Tool}} \cdot v_1} \tag{12}$$

$$t_2 = \frac{A_{\text{Crop}} \cdot x_2}{W_{\text{Tool}} \cdot v_2} \tag{13}$$

$$t_3 = \frac{A_{\text{Crop}} \cdot x_3}{W_{\text{Tool}} \cdot v_3} \tag{14}$$

2) Total time required for all tasks (in this case 3) in an area A_{Crop} per month: t_{Total} (supplementary equation (15))

$$t_{\text{total}} = t_1 + t_2 + t_3 = \frac{A_{\text{Crop}} \cdot x_1}{W_{\text{Tool}} \cdot v_1} + \frac{A_{\text{Crop}} \cdot x_2}{W_{\text{Tool}} \cdot v_2} + \frac{A_{\text{Crop}} \cdot x_3}{W_{\text{Tool}} \cdot v_3} \tag{15}$$

3) Total available working time per month: $t_{\text{available}}$ (supplementary equation (16))

$$t_{\text{available}} = h \cdot d = t_{\text{total}} \tag{16}$$

4) Equate t_{total} and $t_{\text{available}}$ using the term from supplementary equation (16) because they are equal (supplementary equation (17)):

$$\frac{A_{\text{Crop}} \cdot x_1}{W_{\text{Tool}} \cdot v_1} + \frac{A_{\text{Crop}} \cdot x_2}{W_{\text{Tool}} \cdot v_2} + \frac{A_{\text{Crop}} \cdot x_3}{W_{\text{Tool}} \cdot v_3} = t_{\text{available}} \tag{17}$$

5) Solve supplementary equation (17) for Area A_{Crop} (supplementary equation (18)):

$$A_{\text{Crop}} \left(\frac{x_1}{W_{\text{Tool}} \cdot v_1} + \frac{x_2}{W_{\text{Tool}} \cdot v_2} + \frac{x_3}{W_{\text{Tool}} \cdot v_3} \right) = t_{\text{available}} <=> A_{\text{Crop}} = \frac{h \cdot d}{\frac{x_1}{W_{\text{Tool}} \cdot v_1} + \frac{x_2}{W_{\text{Tool}} \cdot v_2} + \frac{x_3}{W_{\text{Tool}} \cdot v_3}} \tag{18}$$

6) If *v* is given in meters per second (m s^{-1}), $h \cdot d$ must be multiplied by a conversion factor of 3600 seconds per hour (s h^{-1}) to ensure that the final unit is m^2 (supplementary equation (19)).

$$A_{\mathrm{Crop}} = \frac{h \cdot d \cdot 3600}{\frac{x_1}{W_{\mathrm{Tool}} \cdot v_1} + \frac{x_2}{W_{\mathrm{Tool}} \cdot v_2} + \frac{x_3}{W_{\mathrm{Tool}} \cdot v_3}} \quad (19)$$

Summarizing supplementary equation (5), supplementary equation (11), and supplementary equation (18) yields general Eq. (1) in the main manuscript.

$$A_{\mathrm{Crop}} = \frac{h \cdot d \cdot 3600}{\sum_{\mathrm{s}=1}^{\mathrm{n}} \frac{x_{\mathrm{s}}}{W_{\mathrm{Tool}} \cdot v_{\mathrm{s}}}} \quad (\mathrm{Eq.1})$$

Supplementary Note 2

This item presents the specific total cost of ownership (TCO) and life cyle assessment (LCA) equations for fixed components, which are only given in general form in the main manuscript (Eqs. 7 and 27).

The cost of the battery c_{Battery} is calculated by multiplying the modelled battery capacity by the specific battery cost per kilowatt-hour (kWh) $c_{\text{Battery, specific}}$ (supplementary equation (20)):

$$c_{\text{Battery}} = capacity_{\text{Battery}} \cdot c_{\text{Battery, specific}} \tag{20}$$

To obtain the cost of the electric motor $c_{\text{Electric motor}}$, the required power is multiplied by the specific electric motor cost $c_{\text{Electric motor, specific}}$ (supplementary equation (21)):

$$c_{Electric\ Motor} = \text{Power}_{\text{Electric motor,s}} \cdot c_{\text{Electric motor,specific}} \tag{21}$$

Adding this result to the cost of the wheels c_{Wheels} returns the total drivetrain costs $c_{\text{Drivetrain}}$ (supplementary equation (22)):

$$c_{\text{Drivetrain}} = c_{\text{Electric motor}} + c_{\text{Wheels}} \tag{22}$$

The total cost for sensors c_{Sensors} is computed by summing the individual costs for all sensors $c_{\text{Sensor, specific}}$ (supplementary equation (23)):

$$c_{\text{Sensors}} = \sum_{\text{c}=1}^{\text{n}} c_{\text{Sensor c,specific}} \tag{23}$$

Adding the cost of the chassis c_{Chassis} (supplementary equation (24)) and the cost of the ballast c_{Ballast} (supplementary equation (25)**Fehler! Verweisquelle konnte nicht gefunden werden.**) to the cost of all tools c_{Tools} (supplementary equation (26)**Fehler! Verweisquelle konnte nicht gefunden werden.**) yields the cost for chassis, ballast (steel only), and tools (supplementary equation (27)**Fehler! Verweisquelle konnte nicht gefunden werden.**):

$$c_{\text{Chassis}} = m_{\text{Aluminum,chassis}} \cdot c_{\text{Aluminum,specific}} + m_{\text{Steel,chassis}} \cdot c_{\text{Steel,specific}} \tag{24}$$

$$c_{\text{Ballast}} = m_{\text{Steel,Ballast}} \cdot c_{\text{Steel,specific}} \tag{25}$$

$$c_{\text{Tools}} = \sum_{\text{c}=1}^{\text{n}} c_{\text{Tool c,specific}} \tag{26}$$

$$c_{\text{Chassis,ballast and tools}} = c_{\text{Chassis}} + c_{\text{Ballast}} + c_{\text{Tools}} \tag{27}$$

Here, *m* stands for the mass and *c* for the (specific) costs of the component.

To calculate the global warming potential 100 (GWP) of the battery, its capacity is multiplied by its unit impact unit_GWP (supplementary equation (28)):

$$\text{GWP}_{\text{Battery}} = \text{capacity}_{\text{Battery}} \cdot \text{unit\_GWP}_{\text{Battery}} \tag{28}$$

The GWP of the electric motor is determined by multiplying its mass by its unit impact (supplementary equation (29)):

$$\mathrm{GWP}_{\mathrm{Electric\ motor}} = m_{\mathrm{Electric\ motor}} \cdot \mathrm{unit\_GWP}_{\mathrm{Electric\ Motor}} \tag{29}$$

To compute the GWP of the wheels, which are assumed to be composed of steel and rubber, the mass of each component is multiplied by its unit impact. For steel, a metal working impact is included (supplementary equation (30)):

$$\mathrm{GWP}_{\mathrm{Wheels}} = m_{\mathrm{Rubber,wheels}} \cdot \mathrm{unit\_GWP}_{\mathrm{Rubber}} + m_{\mathrm{Steel,wheels}} \cdot (\mathrm{unit\_GWP}_{\mathrm{Steel}} + \mathrm{unit\_GWP}_{\mathrm{Steel\ metal\ working}}) \tag{30}$$

Summing the GWP of the electric motor and the wheels yields the GWP of the drivetrain (supplementary equation (31)):

$$\mathrm{GWP}_{\mathrm{Drivetrain}} = \mathrm{GWP}_{\mathrm{Electric\ motor}} + \mathrm{GWP}_{\mathrm{Wheels}} \tag{31}$$

By multiplying the unit impact of sensors by their mass, the GWP of sensors is obtained (supplementary equation (32)):

$$\mathrm{GWP}_{\mathrm{Sensors}} = m_{\mathrm{Sensors}} \cdot \mathrm{unit\_GWP}_{\mathrm{Sensors}} \tag{32}$$

Because the chassis, ballast, and tools are modelled as consisting only of aluminum, steel, rubber, and linear low-density polyethylene (LLDPE), the GWP of chassis, ballast, and tools is computed by summing the individual GWP contributions of each material. As before, this is obtained by multiplying the corresponding mass by its unit impact. A material working impact is included for aluminum, steel, and LLDPE (supplementary equation (33)):

$$\begin{aligned}\mathrm{GWP}_{\mathrm{Chassis\ and\ Tools}} &= m_{\mathrm{Aluminum,chassis\ and\ tools}} \cdot (\mathrm{unit\_GWP}_{\mathrm{Aluminum}} + \mathrm{unit\_GWP}_{\mathrm{Aluminum\ metal\ working}}) + m_{\mathrm{Steel,chassis,ballast,and\ tools}} \\ &\cdot (\mathrm{unit\_GWP}_{\mathrm{Steel}} + \mathrm{unit\_GWP}_{\mathrm{Steel\ metal\ working}}) + m_{\mathrm{Rubber,chassis,and\ tools}} \\ &\cdot \mathrm{unit\_GWP}_{\mathrm{Rubber}} + m_{\mathrm{LLDPE,chassis,and\ tools}} \cdot (\mathrm{unit\_GWP}_{\mathrm{LLDPE}} + \mathrm{unit\_GWP}_{\mathrm{Blow\ moulding}})\end{aligned} \tag{33}$$

Supplementary Table 1: Overview of the modelled proxy components, ballast, and tools, including data sources. All listed components and tools serve as simplified proxies for the modeled robot configuration and, where appropriate, were scaled down accordingly.

Component/Tool for robot task	Specific name and source
Battery	Lithium Nickel-Manganese-Cobalt-Oxide (Li-NMC 811) battery, 600 $Wh \cdot L^{-1}$ (watt-hours per liter), 250 $Wh \cdot kg^{-1}$ (watt-hours per kilogram)[1]
Electric motor	1 kilowatt (kW) brushless direct current (DC) motor[2]
Wheels	13x5.00-6 Lug tires[3]
Sensors	i) RTK-1010 High-Performance Dual-Band global navigation satellite system (GNSS) RTK Module[4]<br>ii) SICK two-dimensional light detection and ranging (2D-LiDAR)-Sensor[5]<br>iii) Zed 2i red-green-blue-depth (RGB-D) camera[6]<br>iv) MTI-7 GNSS/INS (inertial navigation system) inertial measurement unit[7]<br>v) Microcontroller/Central Processing Unit (CPU)[8]
Preparation	Scaled-down disk harrow[9] and steel ballast
Sowing	Bar mounted grain seeder[10] and steel ballast
Mechanical weed control	C-tine cultivator[11]
Chemical weed control	Tank and mounted boom sprayer[12]
Irrigation and fertilization	Trailer-mounted water bowser[13]

Supplementary Table 2: Overview of the 16 impact categories in the Environmental Footprint 3.1 life cycle impact assessment (LCIA) method. For all 16 impact categories, both indicator and unit are provided.

Impact category	Indicator	Unit
acidification	accumulated exceedance (AE)	mol H+ eq. (1 mole Hydrogen-ion equivalent)
climate change	global warming potential 100 (GWP)	kg CO_2 (carbon dioxide) eq.
ecotoxicity: freshwater	comparative toxic unit for ecosystems (CTUe)	CTUe
energy resources: non-renewable	abiotic depletion potential (ADP): fossil fuels	MJ (mega-joule), net calorific value
eutrophication: freshwater	fraction of nutrients reaching freshwater end compartment (P)	kg P (Phosporous) eq.
eutrophication: marine	fraction of nutrients reaching marine end compartment (N)	kg N (Nitrogen) eq.
eutrophication: terrestrial	fraction of nutrients reaching marine end compartment (N)	mol N eq.
human toxicity: carcinogenic	comparative toxic unit for human (CTUh)	CTUh
human toxicity: non-carcinogenic	comparative toxic unit for human (CTUh)	CTUh
ionising radiation: human health	human exposure efficiency relative to U235 (uranium-235)	kBq (kilo-Becquerel) U235 eq.
land use	soil quality index	dimensionless
material resources: metals/minerals	abiotic depletion potential (ADP): elements (ultimate reserves)	kg Sb (Antimony) eq.
ozone depletion	ozone depletion potential (ODP)	kg Chlorofluorocarbon-11 (CFC-11) eq.
particulate matter formation	impact on human health	disease incidence
photochemical oxidant formation: human health	tropospheric ozone concentration increase	kg Non-Methane Volatile Organic Compounds Equivalent (NMVOC) eq.
water use	user deprivation potential (deprivation-weighted water consumption)	m^3 world eq. deprived

Supplementary Table 3: Life cycle assessment (LCA) results for organic crops per kilogram of crop. Results are given for strawberries, onions, lettuce, and peanuts across all 16 impact categories.

Impact category [unit]	Organic strawberry	Organic onion	Organic lettuce	Organic peanut
Acidification [mol H+ eq. per kg]	0.000600617	0.000148129	0.000233611	0.002953793
Climate change [kg CO2eq. per kg]	0.259977458	0.027602875	0.044669017	0.294287084
Ecotoxicity: freshwater [CTUe per kg]	0.846812106	0.100382228	0.182679158	1.225568198
Energy resources: non-renewable [MJ per kg]	3.525513266	0.461993277	0.724332095	4.303560035
Eutrophication: freshwater [kg P eq. per kg]	6.74308E-05	1.68232E-05	2.8116E-05	0.000184766
Eutrophication: marine [kg N eq. per kg]	0.000125591	2.92444E-05	4.61954E-05	0.001540553
Eutrophication: terrestrial [mol N eq. per kg]	0.001217062	0.000290169	0.000437808	0.009886097
Human toxicity: carcinogenic [CTUh per kg]	1.11819E-10	3.0167E-11	4.84781E-11	2.8414E-10
Human toxicity: non-carcinogenic [CTUh per kg]	4.31803E-09	1.28936E-09	1.92392E-09	1.5359E-08
Ionizing radiation: human health [kBq U235 eq. per kg]	0.028307496	0.008476011	0.012593509	0.075356767
Land use [per kg]	6.399257848	0.122726234	0.180857656	18.17445901
Material resources: metals/minerals [kg Sb eq. per kg]	1.07936E-06	2.0539E-07	4.23964E-07	2.04769E-06
Ozone depletion [kg CFC-11 eq. per kg]	1.56498E-09	3.81925E-10	6.06903E-10	5.85405E-09
Particulate matter formation [disease incidence per kg]	7.474E-09	1.78671E-09	2.84303E-09	2.60465E-08
Photochemical oxidant formation: human health [kg NMVOC eq. per kg]	0.000396713	9.29029E-05	0.000143697	0.001058404
Water use [m^3 world eq. deprived per kg]	0.050503935	0.012812858	0.021115019	0.130659173

Supplementary Table 4: LCA results for non-organic crops, expressed per kilogram of crop. Results are given for strawberries, onions, lettuce, and peanuts across all 16 impact categories.

Impact category [unit]	Non-organic strawberry	Non-organic onion	Non-organic lettuce	Non-organic peanut
acidification [mol H+ eq. per kg]	0.001930606	0.00024355	0.00020593	0.00268912
climate change [kg CO2eq. per kg]	0.353773034	0.0358292	0.037228	0.26881213
ecotoxicity: freshwater [CTUe per kg]	7.624755053	0.50626393	0.23709627	1.88875491
energy resources: non-renewable [MJ per kg]	5.006254117	0.58963483	0.61096159	3.96056288
eutrophication: freshwater [kg P eq. per kg]	0.000125124	1.7615E-05	2.3186E-05	0.00015978
eutrophication: marine [kg N eq. per kg]	0.000379654	4.0878E-05	4.0128E-05	0.00132336
eutrophication: terrestrial [mol N eq. per kg]	0.002697663	0.00045505	0.00037395	0.00870863
human toxicity: carcinogenic [CTUh per kg]	3.58682E-10	3.4168E-11	4.1953E-11	2.5119E-10
human toxicity: non-carcinogenic [CTUh per kg]	5.17373E-09	1.2254E-09	1.5978E-09	1.3115E-08
ionizing radiation: human health [kBq U235 eq. per kg]	0.032374563	0.00797336	0.0104893	0.06455345
land use [per kg]	5.806358377	0.17695337	0.1506074	15.4218271
material resources: metals/minerals [kg Sb eq. per kg]	2.63063E-06	3.4607E-07	3.4101E-07	1.9747E-06
ozone depletion [kg CFC-11 eq. per kg]	2.12958E-07	6.5099E-09	3.1455E-09	1.5672E-08
particulate matter formation [disease incidence per kg]	1.7964E-08	2.3082E-09	2.3836E-09	2.3452E-08
photochemical oxidant formation: human health [kg NMVOC eq. per kg]	0.000937868	0.00012033	0.00012152	0.00097171
water use [m^3 world eq. deprived per kg]	0.139162296	0.02374234	0.01848365	0.13036867

Supplementary Table 5: Yearly task frequencies for all crops and corresponding sources used to validate the assumed frequencies. Because strawberries are perennial, preparation and sowing are assumed to occur only once every three years. Strawberries are assumed to be irrigated once per day between April and August, onions once per week between June and July, lettuce 2.5 times per week over six months (sufficient for two growing cycles), and peanuts once every two weeks between May and August. The harvest frequency per year represents the number of times the available working days are reduced by 7.

Crop	Organic (O) / Non-Organic (NO)	Preparation	Sowing	Weed Control	Irrigation	Harvesting
**Strawberry[14,15]**	O	0.33	0.33	5	153	2
	NO	0.33	0.33	3	153	2
**Onion[16–18]**	O	1	1	5	8.71	2
	NO	1	1	2	8.71	2
**Lettuce[19,20]**	O	2	2	6	65.36	4
	NO	2	2	2	65.36	4
**Peanut[21,22]**	O	1	1	3	8.79	2
	NO	1	1	2	8.79	2

Supplementary Table 6: Monthly task frequencies for organic strawberries[14,15]. Because strawberries are perennial, preparation and sowing are assumed to occur once every three years, split across two monthly segments. In months with harvest, the number of available working days is reduced by 7. Decimal values indicate tasks averaged over the corresponding time frame.

	Preparation	Sowing	Weed Control	Irrigation	Harvesting
**January**	0	0	0	0	0
**February**	0	0	0	0	0
**March**	0	0	1	0	0
**April**	0	0	1	30	0
**May**	0.167	0	1	31	0
**June**	0.167	0	0	30	1
**July**	0	0.167	0	31	1
**August**	0	0.167	1	31	0
**September**	0	0	1	0	0
**October**	0	0	0	0	0
**November**	0	0	0	0	0
**December**	0	0	0	0	0

Supplementary Table 7: Monthly task frequencies for non-organic strawberries[14,15]. Because strawberries are perennial, preparation and sowing are assumed to occur once every three years, split across two monthly segments. Chemical weed control is shown in italics. In months with harvest, the number of available working days is reduced by 7. Decimal values indicate tasks averaged over the corresponding time frame.

	Preparation	Sowing	Weed Control	Irrigation	Harvesting
**January**	0	0	0	0	0
**February**	0	0	0	0	0
**March**	0	0	0	0	0
**April**	0	0	*1*	30	0
**May**	0.167	0	1	31	0
**June**	0.167	0	0	30	1
**July**	0	0.167	0	31	1
**August**	0	0.167	1	31	0
**September**	0	0	0	0	0
**October**	0	0	0	0	0
**November**	0	0	0	0	0
**December**	0	0	0	0	0

Supplementary Table 8: Monthly task frequencies for organic onions[16–18]. In months with harvest, the number of available working days is reduced by 7. Decimal values indicate tasks averaged over the corresponding time frame.

	Preparation	Sowing	Weed Control	Irrigation	Harvesting
**January**	0	0	0	0	0
**February**	0	0	0	0	0
**March**	1	0	0	0	0
**April**	0	0.5	0	0	0
**May**	0	0.5	1	0	0
**June**	0	0	1	4.29	0
**July**	0	0	1	4.43	0
**August**	0	0	1	0	1
**September**	0	0	1	0	1
**October**	0	0	0	0	0
**November**	0	0	0	0	0
**December**	0	0	0	0	0

Supplementary Table 9: Monthly task frequencies for non-organic onions[16–18]. Chemical weed control is shown in italics. In months with harvest, the number of available working days is reduced by 7. Decimal values indicate tasks averaged over the corresponding time frame.

	Preparation	Sowing	Weed Control	Irrigation	Harvesting
**January**	0	0	0	0	0
**February**	0	0	0	0	0
**March**	1	0	0	0	0
**April**	0	0.5	0	0	0
**May**	0	0.5	*0.4*	0	0
**June**	0	0	*0.4*	4.29	0
**July**	0	0	*0.4*	4.43	0
**August**	0	0	*0.4*	0	1
**September**	0	0	*0.4*	0	1
**October**	0	0	0	0	0
**November**	0	0	0	0	0
**December**	0	0	0	0	0

Supplementary Table 10: Monthly task frequencies for organic lettuce[19,20]. In months with harvest, the number of available working days is reduced by 7. This study assumes two lettuce growing cycles per year. Decimal values indicate tasks averaged over the corresponding time frame.

	Preparation	Sowing	Weed Control	Irrigation	Harvesting
**January**	0	0	0	0	0
**February**	0	0	0	0	0
**March**	1	0	0	0	0
**April**	0	0.5	0	0	0
**May**	0	0.5	1	11.07	0
**June**	0	0	1	10.7	0
**July**	0	0	1	11.07	1
**August**	1	0.5	0	0	1
**September**	0	0.5	1	10.71	0
**October**	0	0	1	11.07	0
**November**	0	0	1	10.71	1
**December**	0	0	0	0	1

Supplementary Table 11: Monthly task frequencies for non-organic lettuce[19,20]. Chemical weed control is shown in italics. In months with harvest, the number of available working days is reduced by 7. This study assumes two lettuce growing cycles per year. Decimal values indicate tasks averaged over the corresponding time frame.

	Preparation	Sowing	Weed Control	Irrigation	Harvesting
**January**	0	0	0	0	0
**February**	0	0	0	0	0
**March**	1	0	0	0	0
**April**	0	0.5	0	0	0
**May**	0	0.5	*0.33*	11.07	0
**June**	0	0	*0.33*	10.71	0
**July**	0	0	*0.33*	11.07	1
**August**	1	0.5	0	0	1
**September**	0	0.5	*0.33*	10.7	0
**October**	0	0	*0.33*	11.07	0
**November**	0	0	*0.33*	10.71	1
**December**	0	0	0	0	1

Supplementary Table 12: Monthly task frequencies for organic peanuts[21,22]. In months with harvest, the number of available working days is reduced by 7. Decimal values indicate tasks averaged over the corresponding time frame.

	Preparation	Sowing	Weed Control	Irrigation	Harvesting
**January**	0	0	0	0	0
**February**	0	0	0	0	0
**March**	1	0	0	0	0
**April**	0	0.5	0	0	0
**May**	0	0.5	0.75	2.2	0
**June**	0	0	0.75	2.14	0
**July**	0	0	0.75	2.21	0
**August**	0	0	0.75	2.21	0
**September**	0	0	0	0	1
**October**	0	0	0	0	1
**November**	0	0	0	0	0
**December**	0	0	0	0	0

Supplementary Table 13: Monthly task frequencies for non-organic peanuts[21,22]. Chemical weed control is shown in italics. In months with harvest, the number of available working days is reduced by 7. Decimal values indicate tasks averaged over the corresponding time frame.

	Preparation	Sowing	Weed Control	Irrigation	Harvesting
**January**	0	0	0	0	0
**February**	0	0	0	0	0
**March**	1	0	0	0	0
**April**	0	0.5	0	0	0
**May**	0	0.5	*0.5*	2.21	0
**June**	0	0	*0.5*	2.14	0
**July**	0	0	*0.5*	2.21	0
**August**	0	0	*0.5*	2.21	0
**September**	0	0	0	0	1
**October**	0	0	0	0	1
**November**	0	0	0	0	0
**December**	0	0	0	0	0

Supplementary Table 14: Average crop yield per hectare and year based on EU[23] and US[24] data. Organic crop yields are reduced by 16% to account for expected yield losses in this production system[25].

Type of crop	Average yield per hectare (ha), including a 16% yield reduction factor for organic crops [kg ha^{-1}]
Organic Strawberry	11732
Non-Organic Strawberry	13966
Organic Onion	31477
Non-Organic Onion	37473
Organic Lettuce	22528
Non-Organic Lettuce	26819
Organic Peanut	3704
Non-Organic Peanut	4410

Supplementary Table 15: Constant parameters across crops. Parameters without sources are taken directly from the model. *For all tools, costs were estimated from proxy equipment. Where the modeled tool size differed from the proxy, the proxy cost was scaled linearly by tool width. In all cases, costs were then rounded up, and an additional €500 (Euro) surcharge was added to remain conservative and account for uncertainty.

**Parameter [unit]**	w_{Robot} [m]	g [m s^{-2}][26]	r [no unit][27]	$\eta_{Electric\ Motor}$ [no unit][28]
**Value**	0.7	9.81	0.015	0.9
**Parameter [unit]**	$\eta_{Battery}$ [no unit][29]	$c_{Electricity,\ specific}$ [€ kWh^{-1}][30]	$c_{Water,\ specific}$ [€·m^{-3}][31–34]	capacity$_{Battery}$ [kWh]
**Value**	0.95	0.29	0.3	10
**Parameter [unit]**	$c_{Battery,\ specific}$ [€ kWh^{-1}][35–37]	$c_{Electric\ motor,\ specific}$ [€ kW][2,38,39]	c_{Wheels} [€ wheel^{-1}][3,40]	$c_{Sensor\ 1,\ specific}$ [€][4]
**Value**	100	200	40	200
**Parameter [unit]**	$c_{Sensor\ 2,\ specific}$ [€][5,41]	$c_{Sensor\ 3,\ specific}$ [€][6]	$c_{Sensor\ 4,\ specific}$ [€][7,42]	$c_{Sensor\ 5,\ specific}$ [€][8]
**Value**	1750	400	600	1200
**Parameter [unit]**	$m_{Aluminum,\ Chassis}$ [kg]	$c_{Aluminum,\ specific}$ [€ kg^{-1}][43]	$m_{Steel,\ Chassis}$ [kg]	$c_{Steel,\ specific}$ [€ kg^{-1}][44]
**Value**	38.24	2.24	71.22	3.075
**Parameter [unit]**	$c_{Tool\ 1,\ preparation,\ specific}$* [€][9]	$c_{Tool\ 2,\ sowing,\ specific}$* [€][10]	$c_{Tool\ 3,\ mechanical\ weed\ control,\ specific}$* [€][11]	$c_{Tool\ 4,\ chemical\ weed\ control,\ specific}$* [€][12]
**Value**	700	2100	1000	2250
**Parameter [unit]**	$c_{Tool\ 5,\ irrigation\ and\ fertilization,\ specific}$* [€][13]	$c_{Battery\ recycling,\ specific}$ [€ kWh^{-1}][45]	$c_{Aluminum\ recycling,\ specific}$ [€ kg^{-1}][46]	$c_{Steel\ recycling,\ specific}$ [€ kg^{-1}][47]
**Value**	3775	8	1.175	0.486
**Parameter [unit]**	$c_{Rubber\ wheel\ recycling,\ specific}$ [€ wheel^{-1}][48]	$c_{LLDPE\ recycling,\ specific}$ [€ kg^{-1}][49]	$m_{End\text{-}of\text{-}life\ battery}$ [kg]	$m_{End\text{-}of\text{-}life\ aluminum}$ [kg]
**Value**	3	0	40	56.24
**Parameter [unit]**	$m_{End\text{-}of\text{-}life\ steel}$ [kg]	$m_{End\text{-}of\text{-}life\ rubber}$ [kg]	$m_{End\text{-}of\text{-}life\ LLDPE}$ [kg]	$\#_{End\text{-}of\text{-}life\ wheels}$
**Value**	632.22	20	40	8
**Parameter [unit]**	interest rate i[45,50]	maintenance[45,50]	$m_{Electric\ motor}$ [kg][2,39]	$m_{Rubber,\ wheels}$ [kg][51]
**Value**	0.05	0.05	3	10
**Parameter [unit]**	$m_{Steel,\ wheels}$ [kg][51]	$m_{Sensors}$ [kg][4–8]	$m_{Rubber,\ chassis\ and\ tools}$ [kg]	$m_{Aluminum,\ chassis\ and\ tools}$ [kg]
**Value**	4	1.95	10	53.77
**Parameter [unit]**	$m_{Steel,\ chassis\ and\ tools}$ [kg]	m_{LLDPE} [kg]	$m_{Robot,\ no\ tools}$ [kg]	$c_{Labor,\ specific}$ [€/h][52]
**Value**	565.75	40	200	15
**Parameter [unit]**	supervision factor[53]	$m_{Ballast,\ steel\ only}$ [kg]	lifespan$_{Robot}$ [h]	Daily operating hours h [h]
**Value**	0.15	60	5000	18

Supplementary Table 16: Tool-specific parameters for Eq. (14) of the main manuscript. Values were converted to metric units from the American Society of Agricultural and Biological Engineers (ASAE) D497.7 standard[54], assuming medium soil conditions.

Parameter [unit]	k [N m^{-2}] (Newton per square meter)	d [Ns m^{-3}]	e [Ns^2 m^{-4}]
Preparation	27192	6019.2	0
Sowing	50000	0	0
Mechanical weed control	11900	2142	0
Chemical weed control, irrigation, fertilization	0	0	0

Supplementary Table 17: Task-specific parameters. Where appropriate, tool proxies were scaled down in size to match the robot's geometry.

Parameter [unit]	D_s [m]	v_s [m s^{-1}]	$m_{Tools,s}$ [kg]	$m_{Robot,s}$ [kg]
Preparation	0.15[55,56]	0.2777	25[57]	285
Sowing	0.01[54]	0.8333	60[58]	320
Mechanical weed control	0.03[59–61]	0.2778	40[11]	240
Chemical weed control	0	1.6667	450[62]	650
Irrigation, fertilization	0	1.6667	750[13]	950
**Parameter [unit]**	W_{Tool} [m]			
Preparation	0.2			
Sowing	0.6			
Mechanical weed control	0.7			
Chemical weed control	0.7			
Irrigation, fertilization	0.7			

Supplementary Table 18: Crop-specific parameters for strawberries. Parameter values apply to the organic (O), non-organic (NO), or both (B) production systems.

**Parameter [unit]**	$s_{Tool, preparation}$ [m]	$s_{Tool, sowing}$ [# of rows]	$s_{Tool, mechanical weed control}$ [# of rows]	$s_{Tool, chemical weed control}$ [m]
**Value**	0.2	0.75	1	0.7
**Production system (O/NO/B)**	B	B	B	NO
**Parameter [unit]**	$s_{Tool, irrigation and fertilization}$ [m]	V_{Water} [m^3 ha^{-1}][63]	$c_{Pesticide, specific, organic}$ [€ ha^{-1}]	$c_{Pesticide, specific, non-organic}$ [€ ha^{-1}][32]
**Value**	0.7	5000	0	3753
**Production system (O/NO/B)**	B	B	O	NO
**Parameter [unit]**	$c_{Seeds, specific}$ [€ ha^{-1}][32]	$c_{Fertilizer, specific, organic}$ [€ ha^{-1}][64]	$c_{Fertilizer, specific, non-organic}$ [€ ha^{-1}][32]	q_{Seeds} [# of seedlings ha^{-1}][32]
**Value**	7776	10530	1830	45205
**Production system (O/NO/B)**	B	O	NO	B
**Parameter [unit]**	$m_{Pesticide, organic}$ [kg ha^{-1}]	$m_{Pesticide, non-organic}$ [kg ha^{-1}][65,66]	m_{Water} [kg ha^{-1}][63]	$m_{Fertilizer, N}$ [kg ha^{-1}][65,67]
**Value**	0	364	5000000	13.2
**Production system (O/NO/B)**	O	NO	B	B
**Parameter [unit]**	$m_{Fertilizer, P2O5}$ [kg ha^{-1}][65,67]	$m_{Fertilizer, K2O}$ [kg ha^{-1}][65,67]		
**Value**	5.2	18.2		
**Production system (O/NO/B)**	B	B		

Supplementary Table 19: Crop-specific parameters for onions. Parameter values apply to the organic (O), non-organic (NO), or both (B) production systems.

Parameter [unit]	$S_{Tool, preparation}$ [m]	$S_{Tool, sowing}$ [# of rows]	$S_{Tool, mechanical weed control}$ [# of rows]	$S_{Tool, chemical weed control}$ [m]
**Value**	0.2	2	1	0.7
**Production system (O/NO/B)**	B	B	O	NO
**Parameter [unit]**	$S_{Tool, irrigation and fertilization}$ [m]	V_{Water} [m^3 ha^{-1}][68]	$c_{Pesticide, specific, organic}$ [€ ha^{-1}]	$c_{Pesticide, specific, non-organic}$ [€ ha^{-1}][69]
**Value**	0.7	4500	0	628
**Production system (O/NO/B)**	B	B	O	NO
**Parameter [unit]**	$c_{Seeds, specific}$ [€ ha^{-1}][69]	$c_{Fertilizer, specific, organic}$ [€ ha^{-1}][70]	$c_{Fertilizer, specific, non-organic}$ [€ ha^{-1}][69]	q_{Seeds} [kg ha^{-1}][69,71–73]
**Value**	1009	1102	528	1.12
**Production system (O/NO/B)**	B	O	NO	B
**Parameter [unit]**	$m_{Pesticide, organic}$ [kg ha^{-1}]	$m_{Pesticide, non-organic}$ [kg ha^{-1}][74,75]	m_{Water} [kg ha^{-1}][68]	$m_{Fertilizer, N}$ [kg ha^{-1}][76,77]
**Value**	0	27	4500000	117.8
**Production system (O/NO/B)**	O	NO	B	B
**Parameter [unit]**	$m_{Fertilizer, P2O5}$ [kg ha^{-1}][76,77]	$m_{Fertilizer, K2O}$ [kg ha^{-1}][76,77]		
**Value**	95.1	61.4		
**Production system (O/NO/B)**	B	B		

Supplementary Table 20: Crop-specific parameters for lettuce. Parameter values apply to the organic (O), non-organic (NO), or both (B) production systems.

Parameter [unit]	$S_{Tool, preparation}$ [m]	$S_{Tool, sowing}$ [# of rows]	$S_{Tool, mechanical weed control}$ [# of rows]	$S_{Tool, chemical weed control}$ [m]
**Value**	0.2	2	1	0.7
**Production system (O/NO/B)**	B	B	O	NO
**Parameter [unit]**	$S_{Tool, irrigation and fertilization}$ [m]	V_{Water} [m^3 ha^{-1}][78]	$c_{Pesticide, specific, organic}$ [€ ha^{-1}]	$c_{Pesticide, specific, non-organic}$ [€ ha^{-1}][79]
**Value**	0.7	4500	0	1976
**Production system (O/NO/B)**	B	B	O	NO
**Parameter [unit]**	$c_{Seeds, specific}$ [€ ha^{-1}][79]	$c_{Fertilizer, specific, organic}$ [€ ha^{-1}][80]	$c_{Fertilizer, specific, non-organic}$ [€ ha^{-1}][79]	q_{Seeds} [kg ha^{-1}][79,81–83]
**Value**	669	2082	763	0.44
**Production system (O/NO/B)**	B	O	NO	B
**Parameter [unit]**	$m_{Pesticide, organic}$ [kg ha^{-1}]	$m_{Pesticide, non-organic}$ [kg ha^{-1}][84,85]	m_{Water} [kg ha^{-1}][78]	$m_{Fertilizer, N}$ [kg ha^{-1}][86,87]
**Value**	0	8.7	4500000	5.3
**Production system (O/NO/B)**	O	NO	B	B
**Parameter [unit]**	$m_{Fertilizer, P2O5}$ [kg ha^{-1}][86,87]	$m_{Fertilizer, K2O}$ [kg ha^{-1}][86,87]		
**Value**	4.7	2.0		
**Production system (O/NO/B)**	B	B		

Supplementary Table 21: Crop-specific parameters for peanuts. Parameter values apply to the organic (O), non-organic (NO), or both (B) production systems.

**Parameter [unit]**	$s_{Tool, preparation}$ [m]	$s_{Tool, sowing}$ [# of rows]	$s_{Tool, mechanical weed control}$ [# of rows]	$s_{Tool, chemical weed control}$ [m]
**Value**	0.2	1.2	1	0.7
**Production system (O/NO/B)**	B	B	O	NO
**Parameter [unit]**	$s_{Tool, irrigation and fertilization}$ [m]	V_{Water} [m^3 ha^{-1}][88]	$c_{Pesticide, specific, organic}$ [€ ha^{-1}]	$c_{Pesticide, specific, non-organic}$ [€ ha^{-1}][89]
**Value**	0.7	4750	0	295
**Production system (O/NO/B)**	B	B	O	NO
**Parameter [unit]**	$c_{Seeds, specific}$ [€ ha^{-1}][89]	$c_{Fertilizer, specific, organic}$ [€ ha^{-1}][90]	$c_{Fertilizer, specific, non-organic}$ [€ ha^{-1}][89]	q_{Seeds} [kg ha^{-1}][89]
**Value**	216	0	174	112.09
**Production system (O/NO/B)**	B	O	NO	B
**Parameter [unit]**	$m_{Pesticide, organic}$ [kg ha^{-1}]	$m_{Pesticide, non-organic}$ [kg ha^{-1}][91,92]	m_{Water} [kg ha^{-1}][88]	$m_{Fertilizer, N, Organic}$ [kg ha^{-1}][90]
**Value**	0	5.6	4750000	0
**Production system (O/NO/B)**	O	NO	B	O
**Parameter [unit]**	$m_{Fertilizer, P2O5, Organic}$ [kg ha^{-1}][90]	$m_{Fertilizer, K2O, Organic}$ [kg ha^{-1}][90]	$m_{Fertilizer, N, Non-organic}$ [kg ha^{-1}][91,93]	$m_{Fertilizer, P2O5, Non-organic}$ [kg ha^{-1}][91,93]
**Value**	0	0	20.0	18.5
**Production system (O/NO/B)**	O	O	NO	NO
**Parameter [unit]**	$m_{Fertilizer, K2O, Non-organic}$ [kg ha^{-1}][91,93]			
**Value**	33.0			
**Production system (O/NO/B)**	NO			

Supplementary Table 22: LCI for production of 1 kg Li-NMC 811 battery cell. By matching the amount and corresponding unit of the input to the ecoinvent 3.11 process, life cycle inventory assessment results presented in this study can be recreated.

	Description	Unit	Amount	Ecoinvent Process
**Input**	Li-NMC 811 battery cell	kg	1	Market for battery cell, Li-ion, NMC811/Global (GLO)
**Output**	1 kg of Li-NMC 811 battery cell			

Supplementary Table 23: LCI for production of 1 kg electric motor. By matching the amount and corresponding unit of the input to the ecoinvent 3.11 process, life cycle inventory assessment results presented in this study can be recreated.

	Description	Unit	Amount	Ecoinvent Process
**Input**	Electric motor production	kg	1	Electric motor production, for electric scooter/GLO
**Output**	1 kg of electric motor			

Supplementary Table 24: LCI for production of one wheel. By matching the amount and corresponding unit of the input to the ecoinvent 3.11 process, life cycle inventory assessment results presented in this study can be recreated.

	Description	Unit	Amount	Ecoinvent Process
**Input**	Rubber production	kg	2.5	Market for synthetic rubber/GLO
	Steel, low-alloyed	kg	1	Market for steel, low-alloyed/GLO
	Steel refinement	kg	1	Metal working, average for steel product manufacturing/Rest of Europe (RER)
**Output**	1 wheel (3.5 kg total)			

Supplementary Table 25: LCI for production of 1 kg sensors. By matching the amount and corresponding unit of the input to the ecoinvent 3.11 process, life cycle inventory assessment results presented in this study can be recreated.

	Description	Unit	Amount	Ecoinvent Process
**Input**	Electronics, for control units	kg	1	Market for electronics, control units/GLO
**Output**	1 kg of sensors			

Supplementary Table 26: LCI for production of one chassis, one ballast, and all tools for all tasks. By matching the amount and corresponding unit of the input to the ecoinvent 3.11 process, life cycle inventory assessment results presented in this study can be recreated.

	Description	Unit	Amount	Ecoinvent Process
**Input**	Rubber production	kg	10	Market for synthetic rubber/GLO
	Steel, low-alloyed	kg	625.74	Market for steel, low-alloyed/GLO
	Steel refinement	kg	625.74	Metal working, average for steel product manufacturing/RER
	Aluminum, wrought alloy	kg	53.77	Market for aluminum, wrought alloy/GLO
	Aluminum refinement	kg	53.77	Metal working, average for aluminum product manufacturing/RER
	LLDPE granulate	kg	40	Market for polyethylene, linear low density, granulate/GLO
	Blow molding	kg	40	Blow molding/RER
**Output**	1x robot chassis and tools for all tasks			

Supplementary Table 27: LCI for production of 1 kWh electricity. By matching the amount and corresponding unit of the input to the ecoinvent 3.11 process, life cycle inventory assessment results presented in this study can be recreated.

	Description	Unit	Amount	Ecoinvent Process
**Input**	Electricity, low voltage	kWh	1	Market for electricity, low voltage/DE
**Output**	1 kWh of electricity, low voltage			

Supplementary Table 28: LCI for production of one strawberry seedling. By matching the amount and corresponding unit of the input to the ecoinvent 3.11 process, life cycle inventory assessment results presented in this study can be recreated.

	Description	Unit	Amount	Ecoinvent Process
**Input**	Strawberry seedling, for planting	1 Plant	1	Market for strawberry seedling, for planting/GLO
**Output**	1 strawberry seedling			

Supplementary Table 29: LCI for production of 1 kg onion or lettuce seeds. Carrot seeds are used as proxy. By matching the amount and corresponding unit of the input to the ecoinvent 3.11 process, life cycle inventory assessment results presented in this study can be recreated.

	Description	Unit	Amount	Ecoinvent Process
**Input**	Carrot seeds, for sowing	kg	1	Market for carrot seed, for sowing/GLO
**Output**	1 kg of onion or lettuce seeds			

Supplementary Table 30: LCI for production of 1kg peanut seeds. By matching the amount and corresponding unit of the input to the ecoinvent 3.11 process, life cycle inventory assessment results presented in this study can be recreated.

	Description	Unit	Amount	Ecoinvent Process
**Input**	Peanut seed, for sowing	kg	1	Market for peanut seed, for sowing/GLO
**Output**	1 kg of peanut seed			

Supplementary Table 31: LCI for production of 1 kg pesticide. By matching the amount and corresponding unit of the input to the ecoinvent 3.11 process, life cycle inventory assessment results presented in this study can be recreated.

	Description	Unit	Amount	Ecoinvent Process
**Input**	Pesticide, unspecified	kg	1	Market for pesticide, unspecified/GLO
**Output**	1 kg of pesticide			

Supplementary Table 32: LCI for production of 1 kg water. By matching the amount and corresponding unit of the input to the ecoinvent 3.11 process, life cycle inventory assessment results presented in this study can be recreated.

	Description	Unit	Amount	Ecoinvent Process
**Input**	Tap water	kg	1	Market for tap water/Europe without Switzerland
**Output**	1 kg of water			

Supplementary Table 33: LCI for production of 1 kg organic fertilizer N, P_2O_5, and K_2O. By matching the amount and corresponding unit of the input to the ecoinvent 3.11 process, life cycle inventory assessment results presented in this study can be recreated.

	Description	Unit	Amount	Ecoinvent Process
**Input**	Organic nitrogen fertilizer, as N	kg	1	Market for organic nitrogen fertilizer, as N/DE
	Organic phosphorous fertilizer, as P_2O_5	kg	1	Market for organic phosphorous fertilizer, as P_2O_5/DE
	Organic potassium fertilizer, as K_2O	kg	1	Market for organic potassium fertilizer, as K_2O/DE
**Output**	1 kg of organic fertilizer N, 1 kg of organic fertilizer P_2O_5, and 1 kg of organic fertilizer K_2O			

Supplementary Table 34: **LCI for production of 1 kg inorganic fertilizer N, P_2O_5, and K_2O.** By matching the amount and corresponding unit of the input to the ecoinvent 3.11 process, life cycle inventory assessment results presented in this study can be recreated.

	Description	Unit	Amount	Ecoinvent Process
**Input**	Inorganic nitrogen fertilizer, as N	kg	1	Market for inorganic nitrogen fertilizer, as N/DE
	Inorganic phosphorous fertilizer, as P_2O_5	kg	1	Market for inorganic phosphorous fertilizer, as P_2O_5/DE
	Inorganic potassium fertilizer, as K_2O	kg	1	Market for inorganic potassium fertilizer, as K_2O/DE
**Output**	1 kg of inorganic fertilizer N, 1 kg of inorganic fertilizer P_2O_5, and 1 kg of inorganic fertilizer K_2O			

Supplementary Table 35: LCI for end-of-life treatment of one robot, one ballast, and all tool materials. By matching the amount and corresponding unit of the input to the ecoinvent 3.11 process, life cycle inventory assessment results presented in this study can be recreated.

	Description	Unit	Amount	Ecoinvent Process
**Input**	Treatment of used Li-ion battery, hydrometallurgical treatment	kg	40	Treatment of used Li-ion battery, hydrometallurgical treatment/GLO
	Treatment of waste aluminum, municipal incineration	kg	56.24	Treatment of waste aluminum, municipal incineration/Europe without Switzerland
	Treatment of LLDPE	kg	40	Treatment of waste plastic, mixed, recycling/RER
	Treatment of waste rubber, unspecified, municipal incineration	kg	20	Treatment of waste rubber, unspecified, municipal incineration/Europe without Switzerland
	Treatment of waste steel, municipal incineration	kg	632.22	Treatment of waste steel, municipal incineration/Europe without Switzerland
**Output**	Emissions to the environment for treatment of robot and tool materials at end-of-life			

Supplementary References

1. Ruppert, J. *et al.* Analyzing material and production costs for lithium-ion and sodium-ion batteries using process-based cost modeling - CellEst 3.0. *J. Power Sources Adv.* **36,** 100190 (2025).
2. Stepperonline. Bürstenloser Gleichstrommotor mit Kühlerlüfter - 80BLS160F-310-02. Available at https://www.omc-stepperonline.com/de/310v-6000u-min-1-75nm-1100w-4-5a-quadratisch-80-x-80-x-159mm-buerstenloser-gleichstrommotor-mit-kuehlerluefter-80bls160f-310-02 (2026).
3. Deli Tire. Tyre 13 x 5.00-6, 4PR, TL, Deli S-247 - AS for lawn tractor, ride-on lawn mower. Available at https://www.amazon.de/Reifen-13x5-00-6-ST-45-Rasentraktor-Aufsitzrasenm%C3%A4her/dp/B00KNZI7CA?source=ps-sl-shoppingads-lpcontext&ref_=fplfs&psc=1&smid=AEV2V27JI6GWW (2025).
4. Mobile GPS Online. RTK-1010 High-Performance Dual-Band GNSS RTK Module. Available at https://mobilegpsonline.ca/products/rtk-1010-high-performance-dual-band-gnss-rtk-module?variant=40223677841496 (2025).
5. ETOH24. SICK 2D-LiDAR-Sensor TIM100-3010200. Available at https://www.etoh24.de/sick-2d-lidar-sensor-tim100-3010200.html (2025).
6. Stereolabs. ZED 2 - AI Stereo Camera (2025).
7. Farnell. MTI-7-0I-DK XSENS, Development Kit. Available at https://de.farnell.com/en-DE/xsens/mti-7-0i-dk/dev-kit-3-achs-beschl-messer-gyro/dp/3793985 (2025).
8. Lenovo. Lenovo ThinkStation P360 Tiny 30FA004WGE. Available at https://www.amazon.de/dp/B0BRNP7T8Y?m=A3JWKAKR8XB7XF&tag=idealode-am-pk-21&ascsubtag=2025-08-29_59fa4766bde37a3a1b97c7a4a49394218db497964e609700802cbede8ca677e3&th=1&psc=1 (2025).
9. Everythingattachments. Compact Tractor Angle Iron Disc Harrow. Available at https://www.everythingattachments.com/Compact-Tractor-Angle-Iron-Disc-Harrow-p/eta-xdcmp-dh.htm (2026).
10. Terradonis. JPH bar mounted small grain seeeder. Available at https://www.terradonis.com/an-terradonis-jph-small-grain-seeder.html (2025).

11. TOPREGAL. GK6 cultivator for small tractor, 6 tines, TecMaschin. Available at https://www.topregal.com/en/forklift-tractor-attachments/gk6-cultivator-for-small-tractor-6-tines-tecmaschin.html (2025).
12. Machinery Sales EU. CROP SPRAYER 300l tank 6m boom. Available at https://machinerysaleseu.co.uk/product/crop-sprayer-300l-boom-6m/ (2025).
13. Online Tank Store. 500 Litre Water & Drinking Water Highway Bowser. Available at https://www.onlinetankstore.co.uk/product/500-litre-water-drinking-water-highway-bowser/ (2025).
14. Erdbeerprofi. Jahreskalender für Erdbeerpflanzen. Available at https://erdbeerprofi.de/erdbeerprofi-blog/jahreskalender-erdbeerpflanzen (2025).
15. FiBL. Biologischer Erdbeeranbau. Available at https://www.fibl.org/fileadmin/documents/shop/1005-erdbeeren.pdf (2001).
16. Almanac. How to Grow Onions: Plant, Grow & Harvest Big Bulbs. Available at https://www.almanac.com/plant/onions (2025).
17. Haifa Group. Crop Guide: Growing Onions. Available at https://www.haifa-group.com/crop-guide-growing-onions (2025).
18. Oligo, J. B. & Valderama, J. S. Establishing the Planting Calendar for Onions (Allium cepa L) Using Localized Data on Temperature and Rainfall. *Nonlinear Dynamics and Applications,* 895–906 (2022).
19. toom. Salat anpflanzen und ganzjährig ernten. Available at https://toom.de/selbermachen/garten-freizeit/pflanzen/gemuese-anbauen/salat-pflanzen/ (2025).
20. Permapotes. Lettuce: sowing, planting and harvest calendar. Available at https://www.permapotes.com/en/plants/view/lettuce-salad/calendar (2025).
21. Agrocrops. Peanut crop calendar. Available at https://www.agrocrops.com/front/our_quality/1680671117_Crop-calendar.pdf (2025).
22. Almanac. How to Grow Peanuts: Planting, Growing, and Harvesting Guide. Available at https://www.almanac.com/plant/peanuts (2025).
23. eurostat. Crop production in EU standard humidity, 2022.
24. US Department of Agriculture. Peanut Explorer — United States., 2024.

25. Knapp, S. & van der Heijden, M. G. A. A global meta-analysis of yield stability in organic and conservation agriculture. *Nat Commun* **9,** 3632 (2018).

26. NASA. Weight and Mass. Available at https://www.grc.nasa.gov/WWW/K-12/Enginesim/weight_and_mass.htm (2025).

27. Schwehn, J., Ernst, V. & Böttinger, S. A practice-optimized method for measuring the rolling resistance of tractor tires. *Landtechnik* **76,** 142–154 (2021).

28. Kampker, A. & Heimes, H. H. *Elektromobilität. Grundlagen einer Fortschrittstechnologie* (SPRINGER VIEWEG, [S.l.], 2022).

29. Picatoste, A., Schulz-Mönninghoff, M., Niero, M., Justel, D. & Mendoza, J. M. F. Comparing the circularity and life cycle environmental performance of batteries for electric vehicles. *Resour., Conserv. Recycl.* **210,** 107833 (2024).

30. eurostat. Electricity price statistics, 2025.

31. Berbel, J. *et al.* Analysis of irrigation water tariffs and taxes in Europe. *Water Policy* **21,** 806–825 (2019).

32. Bolda, M., Tourte, L., Murdock, J. & Goodrich, B. Sample costs to produce and harvest strawberries (2024).

33. Giannakis, E., Bruggeman, A., Djuma, H., Kozyra, J. & Hammer, J. Water pricing and irrigation across Europe: opportunities and constraints for adopting irrigation scheduling decision support systems. *Water Supply* **16,** 245–252 (2016).

34. Witte, T. de. Wirtschaftlichkeit der Feldbewässerung. Available at https://www.praxis-agrar.de/pflanze/bewaesserung/wirtschaftlichkeit-der-feldbewaesserung (2025).

35. Bloomberg NEF. Lithium-Ion Battery Pack Prices See Largest Drop Since 2017, Falling to $115 per Kilowatt-Hour. *BloombergNEF* (2024).

36. IEA. The battery industry has entered a new phase. Available at https://www.iea.org/commentaries/the-battery-industry-has-entered-a-new-phase (2025).

37. Knehr, K., Kubal, J. & Ahmed, S. Estimated Cost of EV Batteries 2019-2024, 2024.

38. Stepperonline. DSY Serie 1000W CANopen DC Servomotor. Available at https://www.omc-stepperonline.com/de/dsy-serie-1000w-canopen-dc-

servomotor-24-70vdc-3-20nm-3000rpm-17-bit-inkrementalgeber-ip65-dsy-c1000l2a2-m17s (2026).

39. Anaheim Automation. Brushless DC Motor, 1 kW, BLF80D-100-48V-3000. Available at https://anaheimautomation.com/blf80d-100-48v-3000.html (2026).

40. eBay.de. Gepanzerter Reifen Gummi Rad 13x5.00-6 CARLISLE Rasentraktor. Available at https://www.ebay.de/itm/264999097376?_ul=DE&rb_itemId=264999097376&rb_pgeo=DE&toolid=10044&var=0&ff=11 (2025).

41. Farnell Österreich. TIM561-2050101S80 SICK, 2D-LiDAR-Sensor, 10m, Time-of-Flight. Available at https://at.farnell.com/sick/tim561-2050101s80/2d-lidar-sensor-10m-pnp-9-bis/dp/4406305 (2025).

42. Mybotshop.de. Movella MTi-7-5A GNSS/INS. Available at https://www.mybotshop.de/Movella-MTi-7-5A-GNSS-INS-Development-Kit (2025).

43. finanzen.net. Aluminiumpreis in Euro und Dollar. Available at https://www.finanzen.net/rohstoffe/aluminiumpreis (2025).

44. MEPS International. Europe Stainless Steel Prices - 3 Years Historical Data. Available at https://mepsinternational.com/gb/en/products/europe-stainless-steel-prices? (2025).

45. Gutsch, M. & Leker, J. Costs, carbon footprint, and environmental impacts of lithium-ion batteries – From cathode active material synthesis to cell manufacturing and recycling. *Appl. Energy* **353,** 122132 (2024).

46. Deng, L., Johnson, S. & Gencer, E. Environmental-Techno-Economic analysis of decarbonization strategies for the Indian aluminum industry. *Energy Conversion and Management* **274,** 116455 (2022).

47. Medarac, H., Moya Rivera, J. A. & SOMERS, J. Production costs from iron and steel industry in the EU and third countries. *1018-5593* (2020).

48. Gieße, A. Autoreifen richtig entsorgen: So klappt es mit der Altreifenentsorgung. *ADAC* (2025).

49. Hundertmark, T., Prieto, M., Ryba, A., Simons, T. J. & Wallach, J. Accelerating plastic recovery in the United States. *McKinsey & Company* (2019).

50. Schlehuber, S., Gutsch, M., Kronemeyer, N., Frieden, F. & von Delft, S. Assessment of environmental impacts and costs for hydrogen-powered cargo shipping in Europe. *Commun Eng* **4,** 50 (2025).

51. Tractor Supply Company. Ogracwheel Flat Free Turf Tread Tire 13x5.00-6 with ¾ and 5/8 Bearings-3 in. Available at https://www.tractorsupply.com/tsc/product/ogracwheel-flat-free-turf-tread-tire-13x500-6-with-3-4-and-5-8-bearings-3-in-center-hub (2025).

52. Lampridi, M. G. *et al.* A Case-Based Economic Assessment of Robotics Employment in Precision Arable Farming. *Agronomy* **9,** 175 (2019).

53. Maritan, E., Lowenberg-DeBoer, J., Behrendt, K. & Franklin, K. Economically optimal farmer supervision of crop robots. *Smart Agricultural Technology* **3,** 100110 (2023).

54. American Society of Agricultural and Biological Engineers. ASAE D497.7 Standard: Agricultural Machinery Management Data, 2011.

55. Håkansson, I., Stenberg, M. & Rydberg, T. Long-term experiments with different depths of mouldboard ploughing in Sweden. *Soil and Tillage Research* **46,** 209–223 (1998).

56. Gronle, A. *et al.* Effect of ploughing depth and mechanical soil loading on soil physical properties, weed infestation, yield performance and grain quality in sole and intercrops of pea and oat in organic farming. *Soil and Tillage Research* **148,** 59–73 (2015).

57. wupodo.de. Zweischar Beetpflug 20 cm. Available at https://www.wupodo.de/zweischar-beetpflug-20-cm-leichter-kleintraktorpflug-2-schar-135044 (2025).

58. Terradonis. Precision Seeders - JDT. Available at https://www.terradonis.com/ouverturepdf.php?file=catalog-seeders-en-1490093768.pdf (2025).

59. APV America. Tined Weeder AS 300 M1 - Crop Care. Available at https://www.apv-america.com/products/organic-weeding/tined-weeder-as/as-300-m1 (2025).

60. Curran, W. S. Weed Management in Organic Cropping Systems. Available at https://extension.psu.edu/weed-management-in-organic-cropping-systems (2025).
61. Hatzenbichler Agro Technik. Finger weeder. Available at https://www.hatzenbichler.com/en/finger-weeder (2025).
62. Progreen. Fertcast tractor sprayer: 6m boom, 300L tank. Available at https://www.progreen.co.uk/fertcast-tractor-sprayer-6m-boom-300l-tank/.
63. Ali Sarıdaş, M., Kapur, B., Çeliktopuz, E., Şahiner, Y. & Kargı, S. P. Land productivity, irrigation water use efficiency and fruit quality under various plastic mulch colors and irrigation regimes of strawberry in the eastern Mediterranean region of Turkey. *Agricultural Water Management* **245,** 106568 (2021).
64. Bolda, M., Tourte, L., Murdock, J. & Goodrich, B. Sample Costs to Produce and Harvest Organic Strawberries (2024).
65. US Department of Agriculture. Strawberries: Acres Planted. USDA/NASS QuickStats Ad-hoc Query Tool. Available at https://quickstats.nass.usda.gov/results/10752315-E2B9-351F-854F-462C856A3DBB (2023).
66. US Department of Agriculture. Strawberries: Chemical Fungicide, Herbicide, Insecticide and Other. USDA/NASS QuickStats Ad-hoc Query Tool. Available at https://quickstats.nass.usda.gov/results/2C675BA7-F841-3D4E-9A50-33427613BB58 (2023).
67. US Department of Agriculture. Strawberries: Fertilizers. USDA/NASS QuickStats Ad-hoc Query Tool. Available at https://quickstats.nass.usda.gov/results/3BEEBB67-63E7-35A1-9A49-A78E01434E3D (2023).
68. FAO. Onion - Land and Water. Available at https://www.fao.org/land-water/databases-and-software/crop-information/onion/en/ (2025).
69. Cornforth & C, G. Estimated Costs and Returns per Acre: Onion, Hybrid Yellow Varieties, Furrow Irrigated, Rio Grande Valley (2024).

70. Brumfield, R. G. Costs of Production for (Organic) Yellow Onions, Per Acre – Farm Management. Available at https://farmmgmt.rutgers.edu/table-74-costs-of-production-for-yellow-onions-per-acre/ (1996).

71. Osborne Quality Seeds. Seed Count Chart. Available at https://www.osborneseed.com/pages/seed-count-chart (2025).

72. Rupp Seeds. Onions. Available at https://www.ruppseeds.com/vegetables/products/onions (2025).

73. Urban Farmer. Scout, (F1) Onion Seeds. Available at https://www.ufseeds.com/product/scout-f1-onion-seeds/ONSCUT.html (2025).

74. US Department of Agriculture. Onions: Acres Planted. USDA/NASS QuickStats Ad-hoc Query Tool. Available at https://quickstats.nass.usda.gov/results/C2925F37-44A0-3551-905B-30D18B86AB7B (2024).

75. US Department of Agriculture. Onions: Chemical Fungicide, Herbicide, Insecticide and Other. USDA/NASS QuickStats Ad-hoc Query Tool. Available at https://quickstats.nass.usda.gov/results/FBAC3D16-D88C-3415-9652-891E1E61CD54 (2024).

76. US Department of Agriculture. Onions: Acres Planted. USDA/NASS QuickStats Ad-hoc Query Tool. Available at https://quickstats.nass.usda.gov/results/344657D5-4953-32DA-B671-6D6C2578AAA6 (2022).

77. US Department of Agriculture. Onions: Fertilizers. USDA/NASS QuickStats Ad-hoc Query Tool. Available at https://quickstats.nass.usda.gov/results/D0DC344C-D7CC-3F69-BB34-8481F6F61124 (2022).

78. Cahn, M. D., Johnson, L. F. & Benzen, S. D. Evapotranspiration Based Irrigation Trials Examine Water Requirement, Nitrogen Use, and Yield of Romaine Lettuce in the Salinas Valley. *Horticulturae* **8,** 857 (2022).

79. Tourte, L., Smith, R., Murdock, J. & Goodrich, B. Sample Costs to Produce and Harvest File Wrapped Iceberg Lettuce, Central Coast Region — Monterey, Santa Cruz, and San Benito Counties (2023).

80. Tourte, L., Smith, R. F., Klonsky, K. M. & Moura, R. L. de. Sample costs to produce organic leaf lettuce (2009).

81. Rupp Seeds. Lettuce. Available at https://www.ruppseeds.com/vegetables/products/lettuce (2025).

82. Urban Farmer. Iceberg, Lettuce Seeds. Available at https://www.ufseeds.com/product/iceberg-lettuce-seeds---1%2F4-pound/LEIC-1_4-pound.html (2025).

83. Mountain Valley Seed Company. Leaf Lettuce Garden Seeds. Available at https://www.amazon.com/Leaf-Lettuce-Garden-Seeds-Microgreens/dp/B06XQ79CR8?th=1 (2025).

84. US Department of Agriculture. Lettuce: Acres Planted. USDA/NASS QuickStats Ad-hoc Query Tool. Available at https://quickstats.nass.usda.gov/results/E62BE8D8-30E3-34EB-ADD9-711D5659470F (2024).

85. US Department of Agriculture. Lettuce: Chemical Fungicide, Herbicide, Insecticide and Other. USDA/NASS QuickStats Ad-hoc Query Tool. Available at https://quickstats.nass.usda.gov/results/16BFE4B4-CC68-3EF3-97AF-B16877017F00 (2024).

86. US Department of Agriculture. Lettuce: Acres Planted. USDA/NASS QuickStats Ad-hoc Query Tool. Available at https://quickstats.nass.usda.gov/results/40267277-DD8C-322D-B486-FDBB37AC686D (2022).

87. US Department of Agriculture. Lettuce: Fertilizers. USDA/NASS QuickStats Ad-hoc Query Tool. Available at https://quickstats.nass.usda.gov/results/AA6CE2C9-7DA6-3555-9265-E304DB976C81 (2022).

88. University of Georgia. Peanut Irrigation Considerations. Available at https://site.extension.uga.edu/plowpoints/2020/06/peanut-irrigation-considerations/? (2025).

89. Cornforth & C, G. Estimated Costs and Returns per Acre Peanuts (2025).

90. Cornforth & C, G. Estimated Costs and Returns per Acre, Organic Peanuts (2022).

91. US Department of Agriculture. Peanuts: Acres Planted. USDA/NASS QuickStats Ad-hoc Query Tool. Available at https://quickstats.nass.usda.gov/results/470F426C-2738-3382-8670-CF48138AA9C4 (2023).

92. US Department of Agriculture. Peanuts: Chemical Fungicide, Herbicide, Insecticide and Other. USDA/NASS QuickStats Ad-hoc Query Tool. Available at https://quickstats.nass.usda.gov/results/CB89C30D-7601-3CDD-970E-8CB71C26E3C7 (2023).

93. US Department of Agriculture. Peanuts: Fertilizers. USDA/NASS QuickStats Ad-hoc Query Tool. Available at https://quickstats.nass.usda.gov/results/DA4E4B6E-1ED7-36E2-ABC6-D586D0832731 (2023).
