# Supplementary material for: Techno-economic and environmental assessment of next-generation battery-powered farming robots
Source: Nat Commun. 2026 Nov 2;17:11336. doi: 10.1038/s41467-026-78281-7 (PMC13728115; doi:10.1038/s41467-026-78281-7)
Supplement: Supplementary file 2 [file 41467_2026_78281_MOESM2_ESM.pdf]

nature portfolio

Peer Review File

Techno-economic and environmental assessment of next-generation battery-powered farming robots

Corresponding Author: Dr Simon Schlehuber

This file contains all reviewer reports in order by version, followed by all author rebuttals in order by version.

Version 0:

Reviewer comments:

Reviewer #1

(Remarks to the Author)
The topic of assessment of next-generation battery-powered farming robots is interesting. But it is not clear which regions or farming contexts the model actually represents. Since this is a model-based analysis, the authors should clarify the underlying assumptions (e.g. farm size, labour and energy costs) and explain how the model is validated. If the model is mainly exploratory or prospective, this should be stated more explicitly.

1 Need for a more balanced perspective
The abstract and introduction focus mainly on the advantages of autonomous battery-powered robots. Given the scope suggested by the title, the paper would benefit from a more critical discussion of potential drawbacks and challenges, such as reliability, maintenance, charging infrastructure, investment risk, regulatory issues, and the skills required to operate such systems.

2 Choice of crops
The selection of strawberries, onions, lettuce, and peanuts is not sufficiently explained. These crops represent rather specific production systems, while major field crops are excluded. The authors should justify this choice and discuss how it affects the general applicability of the results.

3 Exclusion of harvesting
Harvesting is excluded from the model, but the rationale is not clear. Since harvesting is often a major cost and labour component, the authors should explain why it was omitted and briefly discuss how including harvesting might change the results.

4 Methodological choices (LCA and TCO)
The use of LCA and TCO is reasonable, but the manuscript should explain more clearly why these methods were chosen and what their limitations are in this context. In particular, some broader techno-economic aspects (e.g. organisational change, learning effects, risk) are not captured.

5 Representativeness of the robot model
The robot is described with very specific dimensions (0.85 m × 0.70 m × 0.70 m), which raises questions about how general this model is. It would help to clarify whether this represents a typical class of next-generation robots or a specific prototype, and how transferable the results are across different countries and farm sizes. Are they any other existing models in the world?

6 Theoretical versus real-world basis
It is not entirely clear whether the robot model is based on an existing product or a prototpe. The authors should clarify whether the input data are empirical or simulated, where the data come from, and in which contexts the results can realistically be applied.

7 Missing digital and software-related costs
The authors mention several limitations, but important cost components related to AI, software, data management, and digital infrastructure are not included. Given the focus on next-generation robots, it would be useful to explain why these

costs were excluded and how their inclusion might affect the conclusions.

8 Environmental scope and missing biodiversity assessment
While the paper is presented as an environmental assessment, the environmental analysis appears to be limited to energy use and emissions via LCA. Potential impacts on biodiversity (e.g. field operations, disturbance, cropping patterns, or pesticide use enabled by robotic systems) are not addressed. The authors should either justify why biodiversity impacts are outside the scope of the study or briefly discuss how battery-powered farming robots could affect biodiversity, positively or negatively.

Reviewer #2

(Remarks to the Author)
General assessment
This article examines the highly relevant and rapidly developing topic of the techno-economic and environmental potential of autonomous, battery-powered agricultural robots (ABAR). This ambitious and methodologically comprehensive study covers a wide range of crops and production systems and will undoubtedly contribute to discussions about the future of sustainable, automated, low-impact agriculture. The article is well structured and coherent, and the integrated TCO and LCA analysis is presented clearly and systematically.
The authors are to be commended for their attempt to combine economic and environmental aspects within a single modelling framework that covers the entire crop cycle, from soil preparation to fertilisation and irrigation. Such an integrated approach is essential for making a reasonable assessment of the potential of robotics in agriculture.
However, despite the paper's strengths, the methodology and discussion highlight several key assumptions that I believe are overly optimistic and could significantly impact economic and environmental outcomes. Below, I present the most important comments that I believe would enhance the paper's credibility, balance and scientific value.
Major comments:
1. Literature review and novelty positioning of the work. The authors argue that, to date, the economic and environmental performance of autonomous, battery-powered agricultural robots has not been assessed together. While this work's integrated TCO and LCA analysis covering multiple field operations on a single platform is a valuable contribution, several studies examining individual aspects of autonomous and electric agricultural systems have been published in recent years. Some works provide detailed life cycle analyses of autonomous and electric agricultural platforms, while others examine the economic, techno-economic, and operational aspects of these systems. Therefore, the literature review should be expanded somewhat to more clearly define that the main novelty of this article lies in the integrated modelling framework and analysis of a wide range of field operations rather than in economic or environmental assessments themselves.
e.g.
Saidani, M., Pan, E., Kim, H., Greenlee, A., Wattonville, J., Yannou, B., ... & Cluzel, F. (2020). Assessing the environmental and economic sustainability of autonomous systems: A case study in the agricultural industry. Procedia CIRP, 90, 209-214.
Vahdanjoo, M., Gislum, R., & Sørensen, C. A. G. (2023). Operational, economic, and environmental assessment of an agricultural robot in seeding and weeding operations. AgriEngineering, 5(1), 299-324.
Pradel, M., de Fays, M., Seguineau, C. (2022). Comparative Life Cycle Assessment of intra-row and inter-row weeding practices using autonomous robot systems in French vineyards. Sci. Total Environ. 838, 156441.
Lagnelöv, O., Larsson, G., Larsolle, A., & Hansson, P. A. (2021). Life cycle assessment of autonomous electric field tractors in Swedish agriculture. Sustainability, 13(20), 11285.
Krupanek, J., de Santos, P. G., Emmi, L., Wollweber, M., Sandmann, H., Scholle, K., ... & Andreasen, C. (2024). Environmental performance of an autonomous laser weeding robot—A case study. The International Journal of Life Cycle Assessment, 29(6), 1021-1052.
2. Harvest elimination. The authors argue that the harvest stage differs significantly between crops and therefore eliminate it entirely. However, harvesting is one of the most energy-, economically- and technically-intensive stages of agriculture, particularly when it comes to the cultivation of strawberries, lettuce and onions. Excluding this stage systematically favours the ABAR concept and can significantly distort TCO and LCA comparisons with conventional technologies. It is recommended to consider several alternative harvesting scenarios (e.g. manual, semi-mechanized, robotic) or to provide a sensitivity analysis showing how the results would change if realistic harvest options were included.
3. Sensitivity analysis. The sensitivity analysis shows that the results, both economic and environmental, are most sensitive to variable agro-inputs (seeds, fertilizers, pesticides, water) rather than the technical parameters of the robotic platform itself. This suggests that the primary benefit predicted by the model stems from the assumptions regarding precise agronomic management rather than from the robotics directly. However, this circumstance is not emphasised enough in the discussion, with the results being interpreted as if the main benefit were related to the ABAR technology itself. It is recommended that the contribution of the robotic platform and the agronomic management assumptions are more clearly separated. Additionally, the ±50% variation intervals for all parameters (except for "Hours of operation were only varied by ±25%") are poorly justified and do not always reflect real engineering and agronomic constraints. It would be more appropriate to apply uncertainty intervals based on empirical data for different parameters.
4. Robot lifetime. The authors assume a robot lifetime of 10,000 operating hours. However, given that autonomous agricultural robots are still in the early stages of technological maturity, this seems an overly optimistic assumption, particularly for small, lightweight platforms that are operated intensively in field conditions. Some recent studies (e.g. Pradel et al., 2022) use more conservative assumptions (approximately 5,000 hours) due to limited long-term operational experience. Additionally, the authors refer to Krupanek et al. (2024) work on an autonomous laser weeding robot. However, the mechanical loads and operating conditions of this robot differ fundamentally from those of the multifunctional platform modelled in this study, which performs traction-requiring operations (e.g. plowing). Therefore, it is recommended that more conservative scenarios are considered for the robot's service life, or that the choice of 10,000 hours is additionally justified

with empirical data.
5. Robot mechanical capacity and selected tools. The mass (200 kg), dimensions (0.7 m width) and chassis configuration of the simulated robot raise questions about its ability to perform heavy traction operations, particularly plowing. The two-body plough selected is usually used with tractors that are much heavier and more powerful. It is unclear whether such a lightweight platform could generate sufficient traction force without significant slippage or loss of stability. It is recommended that these assumptions are substantiated further.
6. Water and agrochemical savings assumptions. The authors assume a 50% reduction in pesticide and fertilizer use and a 95% reduction in irrigation water. While a partial reduction in inputs due to precision control is reasonable, such a large reduction in water usage is based solely on an interview with a robotics expert and is not supported by published experimental data, which often indicate 20–50% water savings. This assumption could significantly bias the results of both LCA and TCO. It is recommended to provide a broader literature review or consider more conservative scenarios.
7. Elimination of labor costs. The authors assume that, due to full autonomy, personnel costs can be eliminated. However, in real field conditions, even advanced autonomous systems require constant operator monitoring, periodic intervention, logistics and maintenance. Therefore, assuming 0 € labor costs seems overly optimistic, especially with 18 h daily working time. It is therefore recommended that at least partial personnel costs are considered.
8. Discussion. In the discussion, the authors rightly emphasise that the presented results reflect potential opportunities rather than forecasts for real farms. However, it would be useful to supplement this argument with references to recent empirical studies analysing the differences between theoretical expectations and practical results when applying precision and robotic farming technologies. This would further strengthen the critical discussion presented.
Some other comments:
Some of the economic input data, particularly the costs of organic systems (e.g. Brumfield, 1996; Tourte et al., 2009), are based on rather old sources. In light of the substantial changes in agricultural cost structures over the past 15–30 years, it would be beneficial to verify their relevance or supplement the analysis with more recent data.
Figure 3 shows the TCO and GWP results together on one graph, with different y-axes and patterns. While this allows a lot of information to be included, it can make the results difficult to interpret and compare across crops and systems. It is recommended to consider presenting TCO and LCA results in separate graphs.
Figure 2. A wheeled chassis is modeled, but the illustration shows a tracked prototype, which raises questions in general and regarding the modeling of traction, stability, and contact with the ground.
I think the article is over 5000 words, but maybe I'm not measuring accurately...
Overall, the article is a strong and scientifically valuable piece of work with the potential to make a significant contribution to the field of autonomous agricultural technologies. However, the conclusions are currently based on a set of optimistic assumptions that could lead to an overly favourable assessment of the ABAR concept. Some of these assumptions should be substantiated further, while others should be assessed through sensitivity analysis.
Implementing the above recommendations would strengthen the work both methodologically and interpretively.
A structured version of this review is also provided as a supplementary attachment.

Reviewer #3

(Remarks to the Author)
The combining of LCA and TCO in the field of Ag Automation is certainly a valuable piece of work given the current trajectories of these technologies. By concentrating on specialty crops and AgBots rather than tractors the work complements that of previous economic modelling publications, critically by James Lowenberg-DeBoer and his group.
The results at top level corroborate the findings of those exciting studies which have autonomous cost of production circa £140-120/T in wheat (£0.14/kg), which is encouraging.
The insight that variable components has greatest effect on LCA is very useful when considering the move to battery powered agricultural practice.

However there are a few areas of concern to address:
Firstly, the statement "Because the robot operates fully autonomously, personnel costs are excluded" this is certainly contrary to my extensive experience working in this area and differs from the other economics work mentioned above. Although worker interaction with a field task can be dramatically reduced it is not to 0, I strongly suggest some level of human interaction is considered in this work.
Secondly, the paper shows how important robot parameters (eg size, speed etc) are, I question the some of these points use in the calculations. Critically the base forward speed listed for ploughing and seeding seems optimistic for the zauberzeug Field Friend when their own videos are considered. Of the two robots referenced zauberzeug and FarmDroid I would see the later as a better example to base work on due to its commercial footprint and extensive field time which may better guide baseline values, if commercial farm users are considered. It should also be noted most of this type of "Ag Bots" are establishing crops in land that has been pre tilled by conventional tractors and are not doing the primary tillage themselves (currently).
Finally the idea that irrigation can be applied via a 500L tank is impossibly optimistic, even with the 5% application rate you use (seen in Eq 12) 250,000L/ha needs to be applied in the case of strawberries equating to 500 robot trips/ha/year or 2500 across 5ha you say is achievable. I also question the idea that 5% irrigation is achievable without having a yield effect and am not sure the opinion of a robotics company is good enough reference for this decision.
I would agree 50% pesticide and fertilizer reduction is possible

Version 1:

Reviewer comments:

Reviewer #1

(Remarks to the Author)
The authors have addressed my main comments and the manuscript has improved substantially. The revised version is clearer about its exploratory, scenario-based character and now better acknowledges key assumptions and limitations. I only encourage the authors to ensure that the conclusions clearly distinguish the effects of the robotic platform from those of the assumed reductions in inputs and labour, which appear to drive much of the reported benefit. In addition, comparisons with conventional systems should remain cautiously framed as comparisons with literature-based reference ranges rather than direct predictions for specific farming contexts.

Reviewer #2

(Remarks to the Author)
The revised version of the manuscript is significantly improved compared to the first version. The authors have responded constructively and consistently to most of the reviewers' comments and have strengthened both the methodological soundness and the balance in the interpretation of the results. I particularly appreciate that several key model assumptions have been revised, including the robot's service life, the extent of water-use reduction, the treatment of labor costs, and the modeling of tractor-requiring operations.
The revised manuscript now makes it much clearer that the presented results should be interpreted as exploratory system-level scenarios rather than direct forecasts for actual farms. The discussion of the model's limitations, technological uncertainty, and practical application constraints has also been significantly strengthened. In my opinion, the manuscript has become considerably more balanced, transparent, and scientifically mature.
The authors also more clearly distinguish the impact of the robotic platform itself from the agronomic assumptions related to more precise input management. This significantly improves the overall interpretation of the results.
Below are only a few remaining comments that could further strengthen the manuscript.
1. Crop selection and comparability of scenarios
In the revised version, the authors explain more clearly why highly mechanized commodity crops were not included and why the selected crops are considered potentially suitable for compact ABAR systems. However, the analyzed crops still represent fairly heterogeneous production systems with substantially different agronomic, operational, and especially harvesting characteristics. Although this broadens the exploratory scope of the model, it also increases dependence on crop-specific assumptions and reduces direct comparability across scenarios. It would therefore be useful to briefly acknowledge this trade-off between modeling generality and the realism of specific production systems in the Discussion section.
2. Software and digital infrastructure costs
I appreciate that the authors now clearly acknowledge that the costs associated with external software, cloud computing, connectivity, and data-management infrastructure were not included in the analysis. However, the statement that including these components would likely not substantially alter the main conclusions remains somewhat qualitative, as these costs were not directly modeled. I believe this interpretation could be presented somewhat more cautiously.

Reviewer #3

(Remarks to the Author)
The authors have put considerable effort into responding and amending the areas of concern raised by myself and the fellow reviewers. While the specifics of the amendments may be argued further I believe the paper now presents in publishable form and provides useful insight, combining of LCA and TCO in the field of Ag Automation.

Version 2:

Reviewer comments:

Reviewer #1

(Remarks to the Author)
Thank you for addressing my remaining comments. The revisions have clarified the interpretation of the results and appropriately qualified the comparisons with conventional production. I have no further comments and consider the manuscript suitable for publication.

Reviewer #2

(Remarks to the Author)
Thank you for the revised version of the manuscript. The authors have appropriately addressed my remaining comments. The "Discussion" section now clearly acknowledges the trade-off between a broader selection of crops and the reduced realism and direct comparability of individual crop scenarios. Furthermore, the limitations related to external AI and software costs are presented more cautiously, with proper acknowledgment that their potential impact cannot be fully assessed without direct modeling.

Overall, I believe the manuscript has been significantly improved through the review process. The scope, assumptions, and limitations of the modeling system are now clearly outlined, and the interpretation of the results is appropriately balanced. I

have no further comments and believe that the manuscript, in its current form, is suitable for publication.

Open Access This Peer Review File is licensed under a Creative Commons Attribution 4.0 International License, which permits use, sharing, adaptation, distribution and reproduction in any medium or format, as long as you give appropriate credit to the original author(s) and the source, provide a link to the Creative Commons license, and indicate if changes were made.
In cases where reviewers are anonymous, credit should be given to 'Anonymous Referee' and the source.
The images or other third party material in this Peer Review File are included in the article's Creative Commons license, unless indicated otherwise in a credit line to the material. If material is not included in the article's Creative Commons license and your intended use is not permitted by statutory regulation or exceeds the permitted use, you will need to obtain permission directly from the copyright holder.
To view a copy of this license, visit https://creativecommons.org/licenses/by/4.0/

Dear Reviewers,

Thank you very much for the time, care, and expertise you devoted to reviewing our manuscript. We are grateful for the constructive and thoughtful feedback, which has been very helpful in strengthening the revised paper.

Below, we provide a point-by-point response to the comments and indicate how the manuscript has been revised accordingly.

Reviewer #1

Comment 1.1

The topic of assessment of next-generation battery-powered farming robots is interesting. But it is not clear which regions or farming contexts the model actually represents. Since this is a model-based analysis, the authors should clarify the underlying assumptions (e.g. farm size, labour and energy costs) and explain how the model is validated. If the model is mainly exploratory or prospective, this should be stated more explicitly.

Response 1.1

We thank the reviewer for raising this important overarching point. Several of the requested clarifications are addressed in more detail in our responses to Comments 1.3, 1.6, and 1.7. For ease of reference, we summarize the main revisions here.

i) To make the intended regional and farming-context scope more transparent, we now state more explicitly that the modeled crop-system cases draw on publicly available input and price data intended to reflect conditions common to the southern and western regions of Europe and the United States, while acknowledging that local heterogeneity cannot be fully captured within a single model (Lines 164-167):

Geographic representativeness is approximated by focusing on conditions common to the southern and western regions of Europe and the United States, while recognizing that local climate and management heterogeneity cannot be fully captured within a single model.

ii) We now state more explicitly that the ABAR is treated as a simplified, parameterized representation rather than a one-to-one model of any specific product (Lines 79-81):

The ABAR model in this study reflects recent industrial autonomous farming robots[1,2], but is intended as a simplified, parameterized representation rather than a one-to-one model of any specific product (Figure 2).

iii) We also clarify more explicitly that, unless stated otherwise, model inputs are compiled from literature and database sources and applied within the system of equations described in Methods, with all values documented in the Supplementary Information (Lines 99-103):

Unless stated otherwise, model inputs are compiled from literature and database sources and applied within the system of equations described in Methods. Other parameters reflect the modeling choices required for the scenarios, with all values documented in Supplementary Note 5.

iv) With respect to farm size or operating scale, the model does not impose a fixed farm-size assumption. Rather, the effective scale of operation is derived through the maximum annual coverable area based on crop calendars, task frequencies, operating hours, working width, and task-specific speeds (see section "Maximum coverable area and annual crop yield").

v) We further clarify that the reported costs and impacts are generated by applying the equations to the compiled parameter set rather than from on-farm measurements of a deployed robot (Lines 129-131):

All results are generated by applying these equations to the compiled parameter set (see Supplementary Note 5) rather than from on-farm measurements of a deployed robot.

Taken together, these revisions are intended to make clearer that the study should be interpreted as an exploratory, scenario-based assessment framework rather than a farm-specific predictive model. As already stated in the manuscript, "The quantitative results should therefore be viewed as an exploration of plausible system-level outcomes rather than as forecasts of actual farm-level performance." (Lines 417-419).

Comment 1.2

Need for a more balanced perspective:
The abstract and introduction focus mainly on the advantages of autonomous battery-powered robots. Given the scope suggested by the title, the paper would benefit from a more critical discussion of potential drawbacks and challenges, such as reliability, maintenance, charging infrastructure, investment risk, regulatory issues, and the skills required to operate such systems.

Response 1.2

We thank the reviewer for this helpful suggestion. We agree that the Introduction should more clearly acknowledge practical challenges alongside potential benefits. We therefore added a concise statement in the Introduction noting that adoption depends on reliability and maintenance requirements, charging logistics and infrastructure, upfront investment risk, and regulatory and skills-related constraints (Lines 43-46):

Yet, it remains unclear under which conditions these systems deliver meaningful economic and environmental gains. Their deployment depends not only on technical feasibility, but also on reliability, maintenance, charging logistics, infrastructure, regulation, safety, and operator capabilities[3,4].

We also revised the Abstract to avoid overly affirmative framing and to note more clearly that benefits arise primarily from reducing input use than the platform itself (Lines 12-25):

Decarbonizing agriculture without compromising productivity is a major challenge under rising input costs and labor scarcity. Autonomous battery-powered agricultural robots (ABARs) could help by reducing dependence on conventional machinery and enabling more precise application of inputs, but their system-level economic and environmental performance remains unclear. Here, we integrate total cost of ownership and life-cycle assessment to evaluate a small ABAR for cradle-to-farm-gate crop production of strawberries, onions, lettuce, and peanuts under organic and non-organic management. Across modeled scenarios, benchmarked costs range from 76% below to 17% above conventional production, while greenhouse-gas emissions fall within or below reported conventional ranges. Variable inputs dominate both outcomes, contributing 72–90% of costs and 72–95% of global-warming impacts, with seeds, fertilizers and irrigation water as main drivers. These results show that the value of ABARs depends less on the platform itself than on autonomous operation substantially reducing input use and labor demand.

Comment 1.3

Choice of crops
The selection of strawberries, onions, lettuce, and peanuts is not sufficiently explained. These crops represent rather specific production systems, while major field crops are excluded. The authors should justify this choice and discuss how it affects the general applicability of the results.

Response 1.3

We thank the reviewer for raising this point. We have clarified the rationale for selecting strawberries, onions, lettuce, and peanuts and explained how this choice affects the general applicability of the results. The crop set was chosen to represent production

contexts in which weed control, irrigation, and fertilizer application are important drivers of costs and emissions, rather than highly mechanized major field crops whose production is optimized around high-throughput equipment and much larger annual work capacities. We now clarify in the Discussion that the findings are specific to the modeled task profiles, yields, and input intensities and should therefore not be interpreted as crop-universal estimates. Instead, transferability is expected to be strongest for systems with similar operational characteristics, particularly those in which repeated weed control and input management strongly influence costs and emissions. Highly mechanized commodity field-crop systems are not assessed in this single-ABAR analysis because, under the modeled assumptions, cost-competitive operation in these systems appears less likely (Lines 352-365).

By spanning strawberries, onions, lettuce, and peanuts in both organic and non-organic systems, the analysis shows that ABAR viability depends strongly on crop-specific task profiles and input requirements and may therefore extend beyond a single niche application within the crop-system contexts considered here. While these findings are specific to the modeled task profiles, yields, and input intensities rather than crop-universal estimates, transferability is expected to be strongest for systems with similar operational characteristics. This applies to systems where repeated weed control and input management strongly influence costs and emissions. Highly mechanized commodity field-crop systems are not assessed in this single-ABAR analysis.

Comment 1.4

Exclusion of harvesting
Harvesting is excluded from the model, but the rationale is not clear. Since harvesting is often a major cost and labour component, the authors should explain why it was omitted and briefly discuss how including harvesting might change the results.

Response 1.4

We appreciate this comment and agree that the rationale for excluding harvesting, as well as the implications of that choice, needed to be explained more clearly. Harvesting was excluded from the ABAR platform model because harvesting technologies, labor requirements, and mechanization levels differ strongly by crop and, for several of the crops considered, harvesting remains predominantly manual. A generalized harvesting module would therefore require strong crop-specific assumptions that are both highly uncertain and only weakly aligned with current ABAR capabilities and practice.

At the same time, we appreciate the reviewer's concern that excluding harvesting could affect comparability with conventional systems. Harvesting was already incorporated in the benchmarking step: for TCO, we complemented the ABAR results with crop-specific harvesting cost estimates from the literature to obtain full cradle-to-farm-gate costs (Figure 5), and for GWP we considered the potential influence of harvesting through a literature-based sensitivity assumption. We agree, however, that this

treatment was not explained clearly enough in the original manuscript. We have therefore revised the text to make it more explicit and easier to follow. Specifically, we now:

(i) clarify the system boundary and the rationale for excluding harvesting from the ABAR platform model in the Introduction (Lines 66–71):

The model includes simplified tool and module proxies for preparation, sowing, mechanical and chemical weed control, irrigation, and fertilization, which together define the cradle-to-farm-gate system boundary summarized in **Error! Reference source not found.**. Harvesting is excluded from the ABAR platform model because harvesting technologies, labor requirements, and mechanization levels are strongly crop-specific and, for several of the crops considered, remain partly manual[5–8].

(ii) revise the benchmarking section title and text to make explicit throughout that harvesting is included at the cradle-to-farm-gate comparison level (Lines 245–312):

Benchmarking ABAR-based production against conventional farming including harvesting

[…] Because most conventional cradle-to-farm-gate estimates include harvesting, and because harvesting can represent a substantial share of total production costs and emissions, harvesting is explicitly considered in this benchmarking step to enable a consistent comparison.

[…] The comparison first focuses on TCO. Because harvesting is excluded from the ABAR platform model, it is assumed to be carried out conventionally for each crop.

[…] From a life cycle perspective, comparisons with published values are more difficult than for costs because reported GWP estimates span a wide range and often do not distinguish between organic and non-organic production. Moreover, most conventional cradle-to-farm-gate LCAs include harvesting in the system boundary but rarely report harvesting burdens as a separate contribution. **Error! Reference source not found.** therefore reports the ABAR GWP per kilogram of crop for the modelled field operations and complements these values with a literature-based harvesting sensitivity. Specifically, an upper-bound sensitivity case is included in which harvesting is assumed to account for 30% of total cradle-to-farm-gate GWP[9], enabling a more consistent comparison with conventional cradle-to-farm-gate ranges.

and (iii) now report the harvesting-emissions sensitivity more transparently by adding an explicit upper-bound case based on the assumption that harvesting accounts for 30% of total cradle-to-farm-gate GWP[9]. Accordingly, Table 2 now reports both ABAR GWP excluding harvesting and an estimated total ABAR GWP including harvesting, calculated by treating the modeled non-harvest values as 70% of the total and dividing

by 0.7. This enables a more transparent comparison with conventional cradle-to-farm-gate ranges (Lines 831-836).

Table 1: Comparison of the autonomous battery-powered agricultural robot's (ABAR's) global warming potential (GWP) with conventional reference ranges. Due to limited data for organic crops, the reference ranges do not differentiate between organic and non-organic production. ABAR values are reported for the modelled field operations (excluding harvesting) and as estimated total values assuming that harvesting contributes 30% of cradle-to-farm-gate GWP[9].

Type of crop	ABAR GWP (excl. harvesting) [kg CO_2 eq·kg^{-1} crop]	ABAR GWP (estimated total; assuming harvesting = 30% of total cradle-to-farm-gate GWP)[9] [kg CO_2 eq·kg^{-1} crop]	Conventional GWP (cradle-to-farm-gate range) [kg CO_2 eq·kg^{-1} crop]
Strawberry	Organic: 0.260<br>Non-Organic: 0.354	Organic: 0.371<br>Non-Organic: 0.505	0.11 to 5.48[9]
Onion	Organic: 0.028<br>Non-Organic: 0.036	Organic: 0.039<br>Non-Organic: 0.051	0.14 to 0.324[10–12]
Lettuce	Organic: 0.045<br>Non-Organic: 0.037	Organic: 0.064<br>Non-Organic: 0.053	0.08 to 0.58[10,13–15]
Peanut	Organic: 0.294<br>Non-Organic: 0.269	Organic: 0.420<br>Non-Organic: 0.384	0.311 to 0.547[10,16]

<u>Comment 1.5</u>

Methodological choices (LCA and TCO)
The use of LCA and TCO is reasonable, but the manuscript should explain more clearly why these methods were chosen and what their limitations are in this context. In particular, some broader techno-economic aspects (e.g. organisational change, learning effects, risk) are not captured.

<u>Response 1.5</u>

We thank the reviewer for this helpful suggestion. The revised manuscript now clarifies the rationale for combining LCA and TCO and explicitly delineates the main method limitations. Specifically, the TCO framework quantifies levelized costs per kilogram of crop, while the LCA is expressed consistently as a levelized impact per kilogram of crop. This shared crop-output basis enables direct comparison across crops and production systems and links physical assets, operating inputs, and output within the same cradle-to-farm-gate assessment logic (see Lines 71-76 and Lines 104-150).

We also added brief scope and limitation statements at the end of the TCO and LCA method descriptions, specifying that the analysis does not quantify organizational change, learning effects, or risk and uncertainty beyond the stated assumptions (Lines 123-128 and Lines 146-149).

At the same time, the TCO analysis is limited to direct, modelled cost components and does not capture broader techno-economic dynamics such as organizational change on farms, learning effects and cost reductions over time, or risk and uncertainty beyond

the stated assumptions. These factors may influence implementation and realized costs, but they fall outside the scope of the quantitative TCO model.

[…]

As in the TCO analysis, the LCA quantifies environmental impacts attributable to the defined life cycle flows within the system boundary but does not capture changes over time associated with learning, behavioral adaptation, or organizational change as deployment scales.

Finally, the limitations section now explicitly notes these broader techno-economic and adoption-related factors as outside the quantitative scope of the present model (Lines 408-411).

In addition, adoption-related factors, such as access to capital, infrastructure, and farmer acceptance, and broader dynamics such as organizational change, learning effects, and risk are not considered.

Comment 1.6

Representativeness of the robot model
The robot is described with very specific dimensions (0.85 m × 0.70 m × 0.70 m), which raises questions about how general this model is. It would help to clarify whether this represents a typical class of next-generation robots or a specific prototype, and how transferable the results are across different countries and farm sizes. Are there any other existing models in the world?

Response 1.6

We thank the reviewer for this important point. We have clarified the basis and generality of the robot model in the manuscript. In the robot description, we now state explicitly that the platform dimensions are anchored in an existing compact ABAR platform to ground the geometry[1], while component sizing and operational parameterization were developed for this study based on the assumptions described in Methods rather than by reproducing a single commercial design. We also added that the model is parameterized so that alternative ABAR configurations could be evaluated by substituting key platform and operating parameters (e.g., working width, component masses, battery capacity, and task speeds) (Lines 79-91):

The ABAR model in this study reflects recent industrial autonomous farming robots[1,2], but is intended as a simplified, parameterized representation rather than a one-to-one model of any specific product (Figure 2). Its dimensions (0.85 m × 0.70 m × 0.70 m; L × W × H) are based on an existing compact ABAR platform[1] to anchor the geometry, while the included components, their sizing, and the operational parameterization were defined for this study based on the assumptions described in Methods. The robot runs

on wheels […] The model is structured so that alternative ABAR designs can be evaluated by substituting platform parameters such as working width, component masses, battery capacity, and task speeds.

In the Discussion, we also clarify transferability by noting that the quantitative results reflect a single compact ABAR parameterization and the resulting maximum annual coverable area, and that extending the analysis to other robot sizes or settings would require re-parameterization (e.g., working width, operating speed, and task calendars) (Lines 361-365).

Furthermore, the quantitative results reflect a single compact ABAR parameterization and the resulting maximum annual coverable area; applying the framework to other robot sizes or settings would require re-parameterization, including working width, operating speed, and task calendars.

This complements the existing limitations statement noting that yield and input data are drawn from selected regions and do not capture the full diversity of global conditions (Lines 396-399):

The analysis relies on average yield and input data for selected regions and therefore does not capture the full diversity of soils, climates, management practices, and price structures in global agriculture.

Accordingly, we now note that applying the framework in a region- or country-specific way would require a consistently parameterized case study using locally coherent agronomic and economic assumptions; this is highlighted as a direction for future research (see Outlook, Lines 424-434):

Future research could expand this assessment through region-specific case studies […]

We also appreciate the reviewer's broader point that multiple autonomous field-robot platforms are currently under development. A comprehensive survey of available platforms is outside the scope of this manuscript; instead, our contribution here is a transparent, parameterized assessment framework anchored in one credible compact geometry that can be adapted to assess alternative ABAR designs.

Comment 1.7

Theoretical versus real-world basis

It is not entirely clear whether the robot model is based on an existing product or a

prototype. The authors should clarify whether the input data are empirical or simulated, where the data come from, and in which contexts the results can realistically be applied.

Response 1.7

We appreciate this request for clarification. To make the theoretical versus real-world basis of the robot and the data used more transparent, we now specify that the ABAR reflects recent industrial autonomous farming robots but is treated as a simplified, parameterized representation rather than a one-to-one model of any specific product (Figure 2; Lines 79-81):

The ABAR model in this study reflects recent industrial autonomous farming robots[1,2], but is intended as a simplified, parameterized representation rather than a one-to-one model of any specific product (Figure 2).

We also clarify the origin of the input data: unless stated otherwise, model inputs are compiled from literature and database sources and applied within the system of equations described in Methods, with the remaining parameters reflecting scenario-specific modeling choices and all values documented in Supplementary Note 5 (Lines 99-103):

Unless stated otherwise, model inputs are compiled from literature and database sources and applied within the system of equations described in Methods. Other parameters reflect the modeling choices required for the scenarios, with all values documented in Supplementary Note 5.

In addition, we now state explicitly that the reported costs and impacts are generated by applying the equations to this compiled parameter set rather than from on-farm measurements of a deployed robot (Lines 129-131):

All results are generated by applying these equations to the compiled parameter set (see Supplementary Note 5) rather than from on-farm measurements of a deployed robot.

As now noted in the Discussion (also see Responses 1.3 and 1.6), these findings are specific to the modeled task profiles, yields, and input intensities and are therefore most transferable to systems with similar operational characteristics, particularly where repeated weed control and input management strongly influence costs and emissions. We also clarify that the quantitative results reflect a single compact ABAR parameterization and the resulting maximum annual coverable area. Extending the analysis to other robot sizes or settings would require re-parameterization (Lines 352-365, Combination of Responses 1.3 and 1.6):

By spanning strawberries, onions, lettuce, and peanuts in both organic and non-organic systems, the analysis shows that ABAR viability depends strongly on crop-specific task profiles and input requirements and may therefore extend beyond a single niche application within the crop-system contexts considered here. While these findings are specific to the modeled task profiles, yields, and input intensities rather than crop-universal estimates, transferability is expected to be strongest for systems with similar operational characteristics. This applies to systems where repeated weed control and input management strongly influence costs and emissions. Highly mechanized commodity field-crop systems are not assessed in this single-ABAR analysis. Furthermore, the quantitative results reflect a single compact ABAR parameterization and the resulting maximum annual coverable area; applying the framework to other robot sizes or settings would require re-parameterization, including working width, operating speed, and task calendars.

<u>Comment 1.8</u>

Missing digital and software-related costs
The authors mention several limitations, but important cost components related to AI, software, data management, and digital infrastructure are not included. Given the focus on next-generation robots, it would be useful to explain why these costs were excluded and how their inclusion might affect the conclusions.

<u>Response 1.8</u>

We thank the reviewer for highlighting this point. To address it more explicitly, we now clarify why costs associated with external AI and software infrastructure, including cloud computing, data management, connectivity, and software licensing, were excluded from the analysis and how their inclusion could affect the results. Specifically, we explain that these costs were not parameterized because they are highly deployment-specific and difficult to represent consistently across scenarios. We also clarify that including such costs would increase ABAR-related costs, but that the main conclusions are not expected to change substantially because variable crop-production inputs account for a high share of total costs in the modeled scenarios (Lines 413-417).

These costs were omitted because they are highly deployment-specific and difficult to parameterize consistently across scenarios. Their inclusion would increase ABAR-related costs, but given the dominant contribution of variable crop-production inputs in the modeled scenarios, the main conclusions are unlikely to change substantially.

<u>Comment 1.9</u>

Environmental scope and missing biodiversity assessment
While the paper is presented as an environmental assessment, the environmental analysis appears to be limited to energy use and emissions via LCA. Potential impacts

on biodiversity (e.g. field operations, disturbance, cropping patterns, or pesticide use enabled by robotic systems) are not addressed. The authors should either justify why biodiversity impacts are outside the scope of the study or briefly discuss how battery-powered farming robots could affect biodiversity, positively or negatively.

Response 1.9

We appreciate the reviewer's concern regarding environmental scope. Our study applies a standard multi-category LCA framework with background inventory data representative of global supply chains. In the main text we emphasize 100-year GWP, while additional impact categories are reported in Supplementary Note 2 (Lines 143-146):

The analysis focuses on the 100-year global warming potential (GWP), as it is among the most widely discussed impact categories and highly relevant for policy[17–19]; results for additional impact categories are reported in Supplementary Note 2.

We agree that biodiversity is an important consideration. At the same time, biodiversity outcomes and disturbance-related effects are highly location- and management-specific (e.g., local habitats, species composition, timing and intensity of field operations). Given the cradle-to-farm-gate scope and the use of generic background inventories, quantifying biodiversity endpoints would add substantial uncertainty and could therefore be misleading. For transparency, we report several biodiversity-relevant pressure indicators within the LCA (e.g., land use, eutrophication, and freshwater ecotoxicity) in Supplementary Note 2, while treating explicit biodiversity assessment as outside the scope of the present study.

Reviewer #2

General assessment

This article examines the highly relevant and rapidly developing topic of the techno-economic and environmental potential of autonomous, battery-powered agricultural robots (ABAR). This ambitious and methodologically comprehensive study covers a wide range of crops and production systems and will undoubtedly contribute to discussions about the future of sustainable, automated, low-impact agriculture. The article is well structured and coherent, and the integrated TCO and LCA analysis is presented clearly and systematically.
The authors are to be commended for their attempt to combine economic and environmental aspects within a single modelling framework that covers the entire crop cycle, from soil preparation to fertilisation and irrigation. Such an integrated approach is essential for making a reasonable assessment of the potential of robotics in agriculture.
However, despite the paper's strengths, the methodology and discussion highlight

several key assumptions that I believe are overly optimistic and could significantly impact economic and environmental outcomes. Below, I present the most important comments that I believe would enhance the paper's credibility, balance and scientific value.

Major comments:

Comment 2.1

Literature review and novelty positioning of the work.

The authors argue that, to date, the economic and environmental performance of autonomous, battery-powered agricultural robots has not been assessed together. While this work's integrated TCO and LCA analysis covering multiple field operations on a single platform is a valuable contribution, several studies examining individual aspects of autonomous and electric agricultural systems have been published in recent years. Some works provide detailed life cycle analyses of autonomous and electric agricultural platforms, while others examine the economic, techno-economic, and operational aspects of these systems. Therefore, the literature review should be expanded somewhat to more clearly define that the main novelty of this article lies in the integrated modelling framework and analysis of a wide range of field operations rather than in economic or environmental assessments themselves.

e.g.

Saidani, M., Pan, E., Kim, H., Greenlee, A., Wattonville, J., Yannou, B., ... & Cluzel, F. (2020). Assessing the environmental and economic sustainability of autonomous systems: A case study in the agricultural industry. Procedia CIRP, 90, 209-214.

Vahdanjoo, M., Gislum, R., & Sørensen, C. A. G. (2023). Operational, economic, and environmental assessment of an agricultural robot in seeding and weeding operations. AgriEngineering, 5(1), 299-324.

Pradel, M., de Fays, M., Seguineau, C. (2022). Comparative Life Cycle Assessment of intra-row and inter-row weeding practices using autonomous robot systems in French vineyards. Sci. Total Environ. 838, 156441.

Lagnelöv, O., Larsson, G., Larsolle, A., & Hansson, P. A. (2021). Life cycle assessment of autonomous electric field tractors in Swedish agriculture. Sustainability, 13(20), 11285.

Krupanek, J., de Santos, P. G., Emmi, L., Wollweber, M., Sandmann, H., Scholle, K., ... & Andreasen, C. (2024). Environmental performance of an autonomous laser weeding robot—A case study. The International Journal of Life Cycle Assessment, 29(6), 1021-1052.

Response 2.1:

We thank the reviewer for this helpful observation. We agree that our earlier wording on novelty could be interpreted too strongly and did not sufficiently recognize the existing literature on autonomous and electric agricultural systems. We have therefore revised the Introduction to position the novelty of the article more clearly in the

integrated TCO-LCA framework spanning multiple field operations (Lines 57-62), and added the following text:

Evidence to date remains fragmented. Existing studies have assessed specific robots, regions, or individual operations, or have focused on either environmental or economic performance in isolation[4,20–23]. As a result, ABARs remain insufficiently characterized at the crop-production-system level when environmental and economic outcomes are evaluated together within a single, consistent framework spanning multiple field operations.

Comment 2.2

Harvest elimination.

The authors argue that the harvest stage differs significantly between crops and therefore eliminate it entirely. However, harvesting is one of the most energy-, economically- and technically-intensive stages of agriculture, particularly when it comes to the cultivation of strawberries, lettuce and onions. Excluding this stage systematically favours the ABAR concept and can significantly distort TCO and LCA comparisons with conventional technologies. It is recommended to consider several alternative harvesting scenarios (e.g. manual, semi-mechanized, robotic) or to provide a sensitivity analysis showing how the results would change if realistic harvest options were included.

Response 2.2

We thank the reviewer for raising this related point. Because it overlaps closely with Reviewer #1's comment on harvesting, we refer to Response 1.4 for the full discussion. Briefly, harvesting remains excluded from the ABAR platform model because a generalized set of harvesting scenarios, whether manual, semi-mechanized, or robotic, would require crop-specific assumptions that are highly uncertain and not comparable across the modeled systems. To avoid biasing the comparison, however, harvesting is explicitly re-introduced in the benchmarking step using crop-specific literature values for harvesting costs and a harvesting-emissions sensitivity, thereby enabling a more comparable cradle-to-farm-gate assessment.

Comment 2.3

Sensitivity analysis.

The sensitivity analysis shows that the results, both economic and environmental, are most sensitive to variable agro-inputs (seeds, fertilizers, pesticides, water) rather than the technical parameters of the robotic platform itself. This suggests that the primary benefit predicted by the model stems from the assumptions regarding precise agronomic management rather than from the robotics directly. However, this circumstance is not emphasised enough in the discussion, with the results being

interpreted as if the main benefit were related to the ABAR technology itself. It is recommended that the contribution of the robotic platform and the agronomic management assumptions are more clearly separated. Additionally, the ±50% variation intervals for all parameters (except for "Hours of operation were only varied by ±25%") are poorly justified and do not always reflect real engineering and agronomic constraints. It would be more appropriate to apply uncertainty intervals based on empirical data for different parameters.

Response 2.3

We thank the reviewer for this insightful comment. We agree that the original discussion did not distinguish clearly enough between benefits associated with the robotic platform itself and benefits associated with more precise input management, especially in light of the sensitivity analysis. To address this, the Discussion was revised to make this distinction more explicit and to align the interpretation more closely with the sensitivity analysis. The revised text now states that ABAR-based systems appear particularly favorable where precision management lowers variable-input demand (Lines 349-352):

Under the modeled conditions, ABAR-based production can be cost competitive with conventional crop production while also reducing greenhouse-gas emissions per kilogram of crop, particularly where precision management lowers demand for variable inputs.

We therefore also revised the discussion of cost and impact decomposition to state more clearly that variable inputs contribute more strongly to the results than the technical parameters of the robotic platform itself. The revised text also clarifies that the sensitivity analysis supports this interpretation. Accordingly, the discussion now states that the main modeled benefits arise primarily from more precise management and the resulting reduction in seed, fertilizer, pesticide, and water use (Lines 376-386):

The decomposition into fixed, variable, maintenance, and recycling components, together with the sensitivity analysis, shows that the results are driven more strongly by variable inputs than by the technical parameters of the robotic platform itself. This suggests that, in the modeled scenarios, the main benefits arise less from the platform alone than from more precise management and the associated reductions in seed, fertilizer, pesticide, and water use. At the same time, part of the benefit also reflects the replacement of diesel-powered machinery with an electrified platform. Still, crops with very high input requirements remain both costly and emission-intensive even under the modeled ABAR configuration. This pattern further indicates that not all crops are equally suitable for ABAR-based systems and that careful crop and system selection will be important in practice.

The concluding discussion was also adjusted to avoid overstating the contribution of the platform alone and now emphasizes that favorable outcomes are linked to crop-system contexts with lower variable-input demand and favorable task profiles (Lines 430-434):

Overall, the findings indicate that ABAR-based production systems may be able to reduce production costs and cradle-to-farm-gate emissions relative to conventional farming practices across the crop-system contexts modeled in this study, particularly where autonomous operation is paired with lower variable-input demand and favorable task profiles.

Regarding the second part of the comment, we agree that the earlier use of largely uniform variation intervals did not sufficiently reflect the differing engineering, operational, and agronomic plausibility of the parameter groups. To address this, the sensitivity analysis was revised to retain the grouped scenario structure while replacing the largely uniform intervals with category-specific variation ranges. Narrower bounds are now applied to parameters more strongly constrained by engineering design or operating feasibility, whereas broader bounds are retained for variable input parameters, which are more context dependent across crops, regions, and management conditions (Lines 218-223, Table 1).

For each scenario, and for both TCO and LCA, the relevant parameter groups (see Table 2 for an overview) were varied around their baseline values while all other parameters were held constant. The applied variation ranges differed by scenario, with narrower bounds for parameters more tightly constrained by engineering design or operating feasibility and broader bounds for variable input parameters.

Table 2: Overview of the parameters varied simultaneously in each sensitivity analysis scenario for total cost of ownership (TCO) and life cycle assessment (LCA). Category-specific variation ranges were applied to reflect differing engineering, operational, and agronomic plausibility across parameter groups. See Methods for a description of all parameters.

Name of scenario	Parameters varied	Variation compared to base value
Working hours scenario	**TCO & LCA**: h	±25%
Robot lifespan scenario	**TCO & LCA**: $lifespan_{Robot}$	±25%
Task frequency scenario	**TCO & LCA:** $x_{Weed\ control,\ crop}$, $x_{Watering,\ crop}$	±25%
Robot and tool size scenario	**TCO & LCA:** W_{Robot}, L_{Robot}, H_{Robot}, W_{Tool}, S_{Tool}	±15%
Speed scenario	**TCO & LCA:** $v_{Preparation}$, v_{Sowing}, $v_{Mechanical\ weed\ control}$, $v_{Chemical\ weed\ control}$, $v_{Watering}$	±15%
Variable components scenario	**TCO:** $c_{Electricity,\ specific}$, $c_{Seeds,\ specific,\ crop}$, $c_{Pesticide,\ specific,\ crop}$, $c_{Water,\ specific}$, $c_{Fertilizer,\ specific,\ crop}$ **LCA:** Total electricity consumption per $year_{crop}$, $q_{Seeds,\ crop}$, $m_{Pesticide,\ crop}$, $m_{Water,\ crop}$, $m_{Fertilizer\ (N,\ P2O5,\ K2O),\ crop}$	±50%
Fixed components scenario	**TCO:** $c_{Battery,\ specific}$, $c_{Electric\ motor,\ specific}$, $c_{Wheels,\ specific}$, $c_{Sensors}$, $c_{Aluminum,\ specific}$, $c_{Steel,\ specific}$, c_{Tools} **LCA:** $capacity_{Battery}$, $m_{Electric\ motor}$, m_{Rubber}, $m_{Aluminum}$, m_{Steel}, m_{LLDPE}	±30%

Recycling components scenario	**TCO:** $c_{Battery\ recycling,\ specific}$, $c_{Wheel\ recycling,\ specific}$, $c_{Aluminum\ recycling,\ specific}$, $c_{Steel\ recycling,\ specific}$, $c_{LLDPE\ Recyling,\ specific}$ **LCA:** $m_{Battery,\ end\text{-}of\text{-}life}$, $m_{Aluminum,\ end\text{-}of\text{-}life}$, $m_{Steel,\ end\text{-}of\text{-}life}$, $m_{Rubber,\ end\text{-}of\text{-}life}$, $m_{LLDPE,\ end\text{-}of\text{-}life}$	±30%

Figure 4 and the corresponding discussion in the sensitivity analysis section were updated accordingly to reflect the revised variation ranges (Lines 228-244).

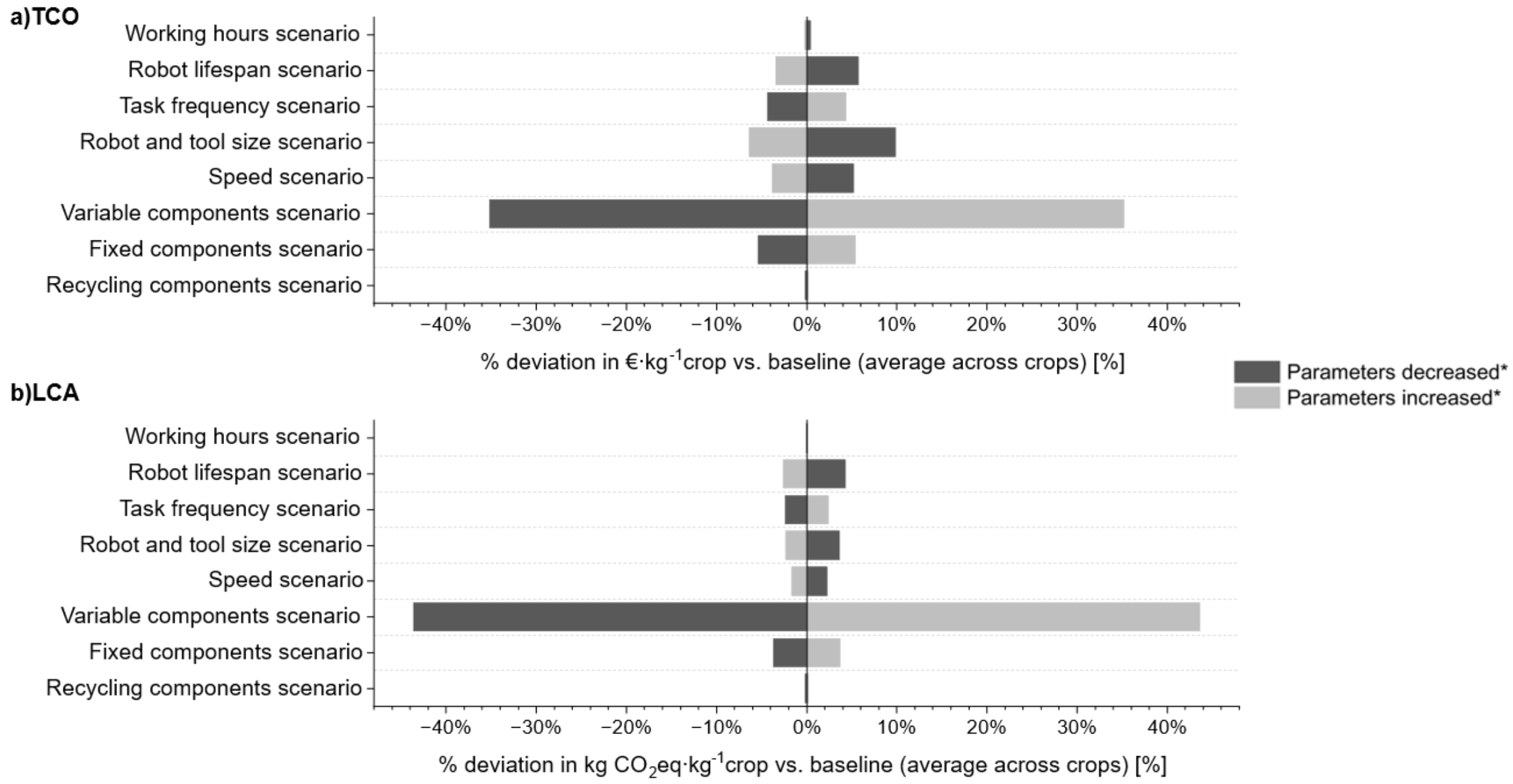

Figure 1: Sensitivity analysis results for a) total cost of ownership (TCO) and b) life cycle assessment (LCA). Each scenario represents a group of parameters varied simultaneously while all other parameters are held constant. *Category-specific variation ranges were applied to the parameters to reflect differing engineering, operational, and agronomic plausibility across parameter groups (see Table 2)

Because the applied variation ranges differ across scenarios, the resulting deviations reflect both model responsiveness and the size of the variation range. Under this setup, the variable components scenario, varied by ±50%, produces the largest deviations from the baseline for both TCO (±35%) and GWP (±44%). This is consistent with the dominant contribution of variable inputs to both outcomes. It further indicates that changes in the prices, quantities, or application rates of seeds, fertilizers, pesticides, water, and electricity strongly affect overall TCO and GWP. The robot and tool size scenario, despite being varied by only ±15%, also shows a clear influence (TCO: +10%/-6%; GWP: +4%/-2%), underlining the importance of the platform's physical configuration. The speed scenario, likewise varied by ±15%, produces noticeable deviations (TCO: +5%/-4%; GWP: +2%/-2%), while the robot lifespan scenario, varied by ±25%, also affects both outcomes (TCO: +6%/-3%; GWP: +4%/-3%). Task frequency, varied by ±25%, and fixed components, varied by ±30%, lead to more moderate deviations, whereas changes in working hours (±25%) and recycling components (±30%) have only a minor effect on the results. Overall, TCO and GWP

respond in broadly similar directions across scenarios, indicating that the main economic drivers are also the main drivers of environmental impacts.

Comment 2.4

Robot lifetime.

The authors assume a robot lifetime of 10,000 operating hours. However, given that autonomous agricultural robots are still in the early stages of technological maturity, this seems an overly optimistic assumption, particularly for small, lightweight platforms that are operated intensively in field conditions. Some recent studies (e.g. Pradel et al., 2022) use more conservative assumptions (approximately 5,000 hours) due to limited long-term operational experience. Additionally, the authors refer to Krupanek et al. (2024) work on an autonomous laser weeding robot. However, the mechanical loads and operating conditions of this robot differ fundamentally from those of the multifunctional platform modelled in this study, which performs traction-requiring operations (e.g. plowing). Therefore, it is recommended that more conservative scenarios are considered for the robot's service life, or that the choice of 10,000 hours is additionally justified with empirical data.

Response 2.4

We thank the reviewer for this important comment. We agree that a more conservative treatment of robot lifetime is appropriate here given the limited long-term operational evidence currently available for ABARs and the reviewer's point that durability expectations from other robotic applications are not directly transferable to a multifunctional platform performing traction-requiring field operations. The original 10,000-hour assumption was selected based on recent literature and industry-oriented durability indications that suggest higher service-life values[2,20,24], whereas the lower values reported in earlier studies provide a more conservative basis for the present analysis. To address the reviewer's concern directly, the base-case robot lifetime was therefore reduced to 5,000 operating hours, in line with lower reported lifetime assumptions[21,23] (Line 474).

Following prior literature[21,23], the lifespan of the robot is set to 5,000 hours.

In addition, a dedicated lifespan category was added to the sensitivity analysis, varying robot lifespan by +/-25% around the base case (see Table 1 and Figure 4, Response 2.3). This range captures both conservative and moderately higher service-life assumptions discussed in the literature, thereby making the effect of lifetime uncertainty on overall cost and emission results more transparent while avoiding an optimistic base-case durability assumption for an early-stage field robot.

Comment 2.5

Robot mechanical capacity and selected tools.

The mass (200 kg), dimensions (0.7 m width) and chassis configuration of the simulated robot raise questions about its ability to perform heavy traction operations, particularly plowing. The two-body plough selected is usually used with tractors that are much heavier and more powerful. It is unclear whether such a lightweight platform could generate sufficient traction force without significant slippage or loss of stability. It is recommended that these assumptions are substantiated further.

Response 2.5

We thank the reviewer for this important point. After revisiting the traction-dependent operations, we found that the original moldboard-plow-based parameterization was not mechanically consistent with the compact 200 kg ABAR platform. In particular, a wheel-load-based traction sanity check (see equation below) indicated that the originally assumed combination of tool type, working width, and working depth implied a tractive demand that was too high for the modeled platform under the stated soil conditions. We therefore revised the configuration rather than retaining it.

In response, we substantially revised the traction-relevant parts of the model. First, the original moldboard-plow proxy was replaced by a lower-draft disk-harrow-based primary tillage proxy (see Supplementary Note 1). Second, the dimensions and operating parameters of this soil-preparation step were reduced to a narrower and shallower configuration that is more consistent with the compact platform scale. Specifically, the revised soil-preparation step is now modeled with a working width of 20 cm (instead of 70 cm), a working depth of 15 cm (instead of 20 cm), and a reduced operating speed of 1 km h^{-1} (instead of 1.5 km h^{-1}). In addition, we resized the seeding unit from 70 cm to 60 cm and reduced sowing speed from 4 km h^{-1} to 3 km h^{-1} because sowing represented the next most traction-relevant operation in the model.

We also revised the propulsion-related components accordingly. Because the reduced tillage and sowing loads lowered the peak power requirement, the electric motor and battery were resized to match the updated operating profile. To avoid lowering platform mass to a level that would again create traction limitations, the chassis was made more robust, bringing the base robot mass back to 200 kg. In addition, we added a steel ballast component (an additional 60 kg of weight) for soil preparation and sowing to increase robot traction and maintain sufficient wheel load.

To substantiate the revised configuration, we applied a wheel-load-based traction consistency check using the relation $H = \mu W$, where *H* is available tractive force, *μ* is the equivalent coefficient of friction, and *W* is wheel load[25]. Under the revised parameterization and across all tasks, the maximum required force is approximately **1050 N.** Because our modelling approach does not allow calculation of wheel-by-wheel load transfer or axle-specific weight distribution, wheel load was approximated as the total robot weight supported by the ground, i.e. $W \approx mg = 260 \cdot 9.81 = \mathbf{2551\ N}$. For

medium soil (μ = 0.43)[25], this gives an available traction of approximately **1097 N**. This revised setup is therefore mechanically more consistent with the modeled compact ABAR than the original moldboard-plow case. Although this is an optimistic simplification, it is the most transparent first-order estimate possible within the current model structure. Based on these changes, we have adjusted the relevant equations and updated parameterization in the Supplementary Information and Methods sections.

At the same time, we do not wish to overstate the degree of mechanical validation achieved here. Slip behavior and field stability depend on factors such as wheel-soil interaction, dynamic load transfer, and field conditions, which are not resolved in the current model. We therefore now clarify this explicitly as a limitation rather than implying full mechanical validation (Lines 404-408).

Consistent with this level of abstraction, traction-dependent operations are represented through draft-force and rolling-resistance calculations, but slip behavior and field stability are not empirically validated. These effects depend on wheel-soil interaction, dynamic load transfer, and field conditions, which fall outside the scope of the present modeling framework.

These revisions increase modeled costs and GWP per kilogram because of the updated tool, chassis, and ballast assumptions, but they do not materially alter the study's main comparative conclusions. The more pronounced effect is on maximum annual coverable area: due to the reduced tool width and lower operating speed, soil preparation becomes the time-limiting task across scenarios (with strawberries representing the only case in which irrigation contributes jointly to the bottleneck), resulting in lower maximum annual coverable areas and therefore lower total crop yields. To reflect this change, we have adjusted the section on "Maximum coverable area and annual crop yield" (Lines 325-338) as well as Figure 7.

The resulting coverable areas differ across crops. Strawberries exhibit the lowest annual coverable area, whereas lettuce achieves the highest. These differences arise primarily from the timing and combination of the most time-intensive tasks within the crop calendar. For strawberries, the bottleneck month combines the two most time-consuming operations, soil preparation and irrigation, which reduces the area the ABAR can service. For onions and peanuts, by contrast, the minimum coverable area is determined by the month in which soil preparation alone is the limiting operation, resulting in identical maximum annual coverable areas for both crops. No divergence is observed between organic and non-organic systems because the maximum annual coverable area is determined by the time-limiting operations of soil preparation or irrigation, which occur with the same annual frequency for both systems. Lettuce reaches the highest coverable area because, under the assumed crop calendar, its two growing cycles per year still allow a larger total annual service area than the other modeled crops.

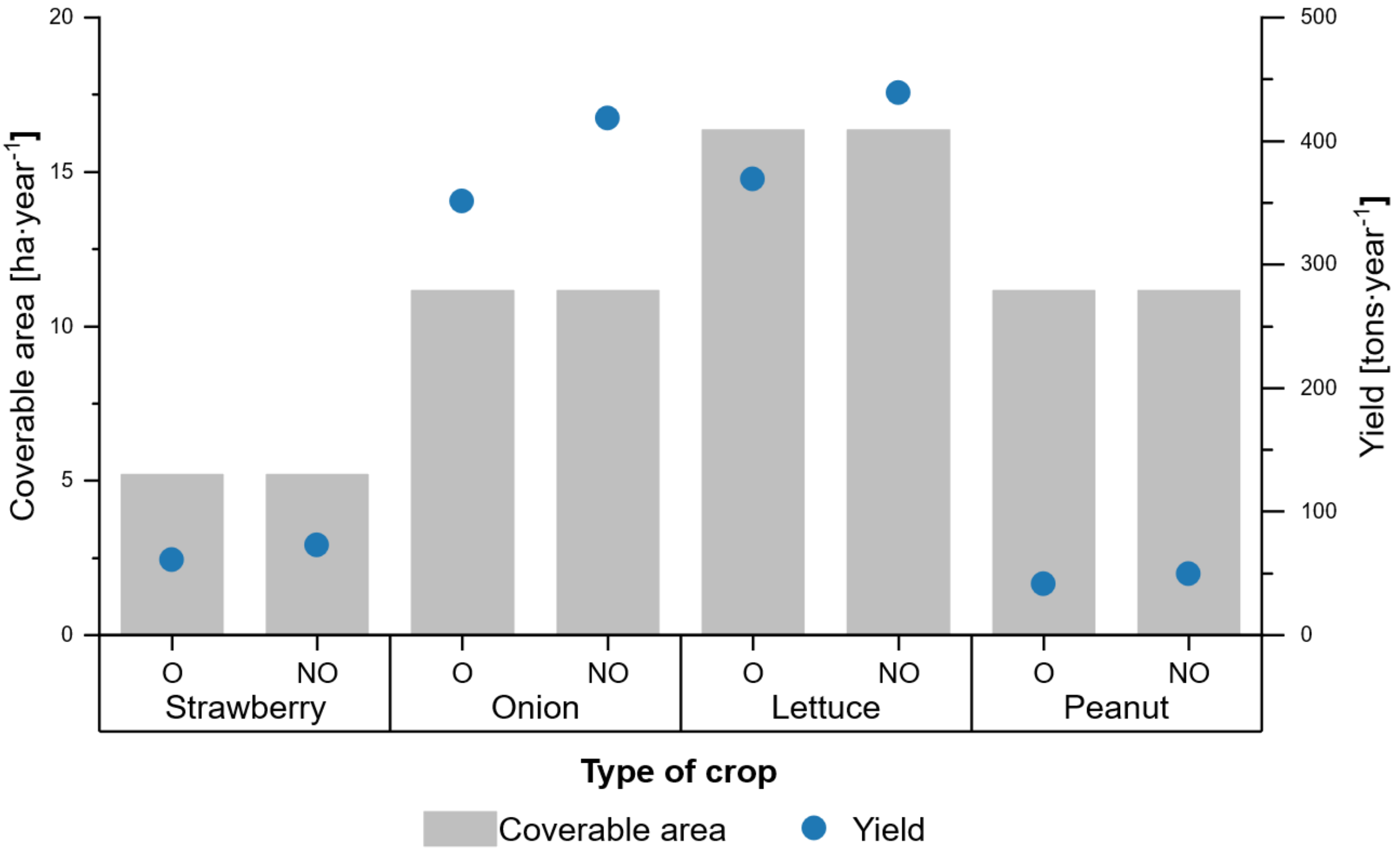

Figure 2: Overview of the robot's coverable area and crop yield per year. Gray bars represent the maximum coverable area per year while blue dots show the overall yield per crop type and production system (O: Organic, NO: Non-organic).

This revision also makes clearer that inclusion of heavy soil-engaging operations in the system boundary can strongly constrain annual throughput for a compact single-ABAR configuration. Future models of small ABARs may therefore choose to exclude soil preparation from the system boundary. We nevertheless retain this step in the present model because it makes this limitation visible (Lines 426-430).

Future models of small ABARs may also choose to exclude soil preparation from the system boundary, as heavy soil-engaging operations can strongly constrain annual throughput in compact single-ABAR configurations. This step was nevertheless deliberately retained in the present study so that the associated limitation would remain visible.

Comment 2.6

Water and agrochemical savings assumptions.

The authors assume a 50% reduction in pesticide and fertilizer use and a 95% reduction in irrigation water. While a partial reduction in inputs due to precision control is reasonable, such a large reduction in water usage is based solely on an interview with a robotics expert and is not supported by published experimental data, which often indicate 20–50% water savings. This assumption could significantly bias the results of

both LCA and TCO. It is recommended to provide a broader literature review or consider more conservative scenarios.

Response 2.6

We appreciate this comment and agree that the original 95% water-saving assumption was too optimistic relative to the published evidence. While substantial reductions in water use may be achievable under highly targeted robotic irrigation strategies, the literature more commonly reports lower savings[26,27]. We have therefore revised the base-case water-reduction assumption from 95% to 50%, aligning it with a more cautious interpretation of the literature and with the reduction assumed for other variable inputs. This revision reduces the risk that the TCO and LCA results are biased by an overly favorable irrigation assumption while retaining the underlying premise that precision robotic application can lower input demand. Lower and higher water-use assumptions continue to be examined in the sensitivity analysis. The revised assumption is now reflected throughout the model (Lines 489-493 and Lines 572-573):

Irrigation water costs (Eq. (1)) are computed in a similar way, with the additional parameter $V_{\text{Water, crop}}/\text{ha}$ representing the amount of water needed per hectare. As for fertilizer and pesticide use, water use is assumed to be 50% lower than under conventional practice, placing this assumption within the range of savings reported in the literature[26,27].

$$c_{\text{Water,crop}} = 0.5 \cdot A_{\text{Crop}} \cdot \frac{V_{\text{Water,crop}}}{\text{ha}} \cdot c_{\text{Water,specific}} \quad (1)$$

$GWP_{\text{Water, crop}}$ (Eq. (2)) is the product of crop-specific water mass $m_{\text{Water, crop}}$, A_{Crop}, and the unit impact of water, multiplied by 0.5 to reflect the ABAR's reduced water use.

$$GWP_{\text{Water,crop}} = 0.5 \cdot m_{\text{Water,crop}} \cdot A_{\text{Crop}} \cdot unit\_GWP_{\text{Water}} \quad (2)$$

Comment 2.7

Elimination of labor costs.

The authors assume that, due to full autonomy, personnel costs can be eliminated. However, in real field conditions, even advanced autonomous systems require constant operator monitoring, periodic intervention, logistics and maintenance. Therefore, assuming 0 € labor costs seems overly optimistic, especially with 18 h daily working time. It is therefore recommended that at least partial personnel costs are considered.

Response 2.7

We thank the reviewer for raising this issue. We agree that assuming zero labor cost is not appropriate for current field conditions. Although the ABAR is modeled as autonomous during field operations, its use still requires setup, supervision, occasional intervention, and logistical support. We have therefore revised the manuscript and the model to include labor as part of OPEX rather than excluding personnel costs altogether (Lines 110-114):

OPEX includes electricity, seeds, pesticides, water, fertilizer, and labor. Although the robot is modeled as autonomous during field operations, labor costs are not assumed to be zero because operation still requires setup, supervision, occasional intervention, and logistical support[23,28].

To make the treatment of labor in relation to the environmental assessment clearer, we now state explicitly that labor is included in the TCO but excluded from the LCA, where it is not modeled as a direct emissions-generating flow (Lines 137-140):

These fixed, variable, and end-of-life components follow the same general structure as the CAPEX, OPEX, and recycling terms used in the cost analysis, with the exception of labor. Labor is excluded from the LCA because it is not typically treated as a direct emissions-generating flow[29].

We also clarified in the Results section that the assumed daily operating time of 18 hours does not imply one-to-one labor time, because labor is modeled separately through a supervision factor rather than being equated directly with robot operating time (Lines 168-171):

Key operating parameters include a daily operating time of 18 hours, a task-specific working width ranging from 0.2 m to 0.7 m, and the task-specific field speeds defined in Methods. Labor is treated separately in the TCO model through a supervision factor rather than being assumed to scale one-to-one with robot operating time.

Figure 3 was revised accordingly to include labor as part of OPEX, and the Results section was updated to reflect the resulting changes in modeled costs:

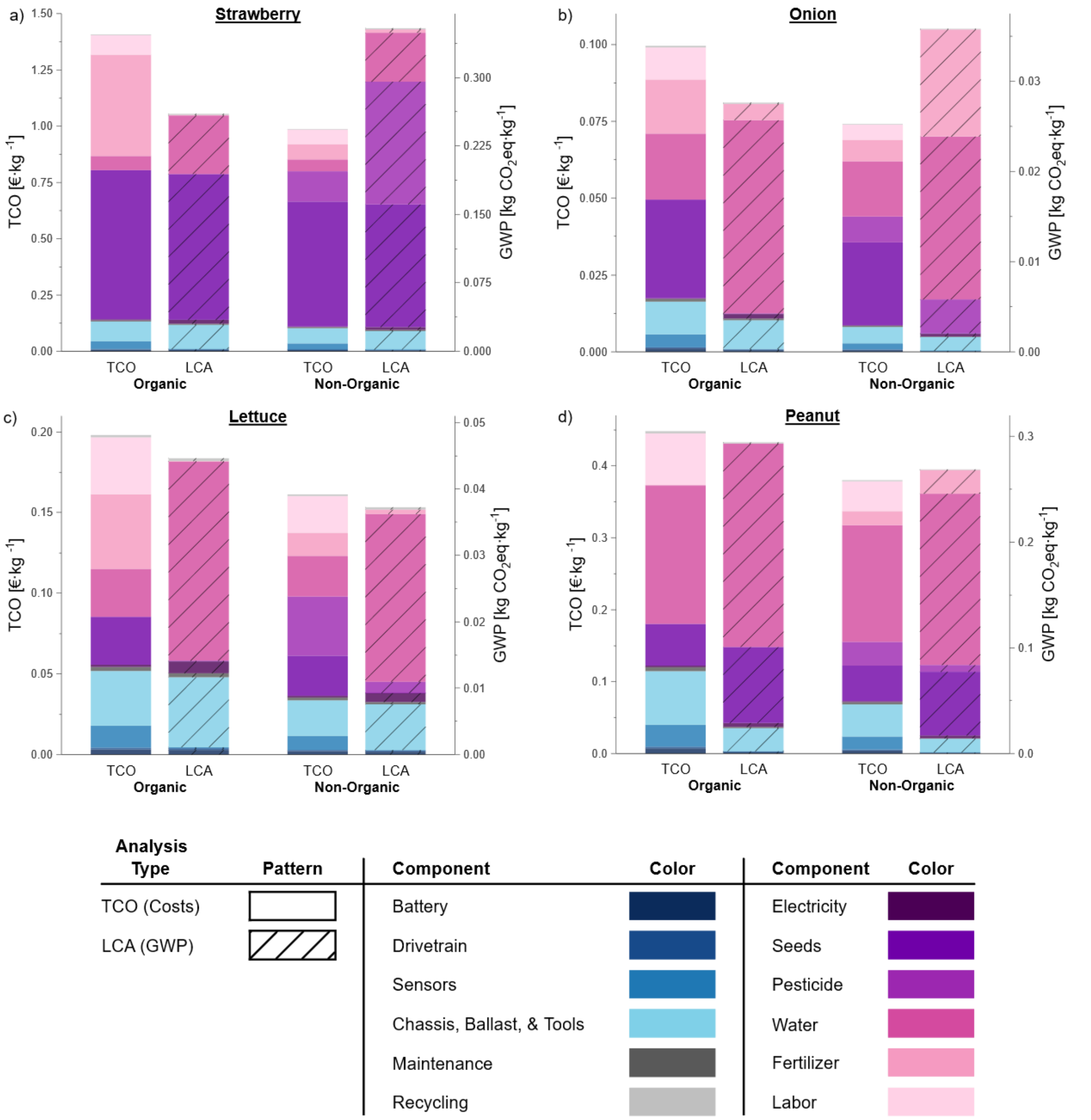

Figure 3: Results of the life cycle assessment (LCA) and total cost of ownership (TCO) analysis for each crop and production system using the modelled autonomous battery-powered agricultural robot. Modelled tasks include preparation, sowing, weed control, irrigation and fertilization. Impacts and costs of fixed components are shown in blue tones, variable components in purple, and maintenance and recycling in gray. Panels show a) strawberries, b) onions, c) lettuce, and d) peanuts.

We further revised the Methods section to include labor explicitly in the OPEX formulation (Lines 483-484):

OPEX comprises the crop-specific costs of electricity, seeds, pesticides, water, fertilizers and labor (Eq. (3)).

$$OPEX_{\mathrm{Crop}} = c_{\mathrm{Electricity,crop}} + c_{\mathrm{Seeds,crop}} + c_{\mathrm{Pesticide,crop}} + c_{\mathrm{Water,crop}} + c_{\mathrm{Fertilizer,crop}} + c_{\mathrm{Labor,crop}} \quad (3)$$

Finally, we added a dedicated labor-cost equation and defined the underlying assumptions used in the model (Lines 494-499):

Labor costs (Eq. (4)) are calculated from the robot's crop-specific annual working time $t_{\text{Total, crop}}$, the hourly labor cost $c_{\text{Labor, specific}}$, and a supervision factor. Although the ABAR is modeled as autonomous during field operations, labor is not assumed to be zero because operation still requires setup, supervision, occasional intervention, and logistical support[23,28]. In the base case, the supervision factor is set to 15% of crop-specific annual robot working time[30].

$$c_{\text{Labor}} = t_{\text{Total,crop}} \cdot c_{\text{Labor,specific}} \cdot supervision\ factor \quad (4)$$

For transparency, we also document the numerical assumptions in the SI, where the hourly labor cost is set to 15 EUR/h[31] and the supervision factor to 0.15[30].

Comment 2.8

Discussion.

In the discussion, the authors rightly emphasise that the presented results reflect potential opportunities rather than forecasts for real farms. However, it would be useful to supplement this argument with references to recent empirical studies analysing the differences between theoretical expectations and practical results when applying precision and robotic farming technologies. This would further strengthen the critical discussion presented.

Response 2.8

We thank the reviewer for this suggestion. We revised the Discussion by adding a sentence that links the existing cautionary interpretation to recent empirical and review-based evidence. The added text clarifies that realized economic and environmental benefits are heterogeneous and context dependent, and that practical uptake of field robots depends on factors such as investment cost, reliability, and farm-specific fit (Lines 419-423). This addition was intended to strengthen the statement that the results reflect potential opportunities rather than forecasts of actual farm-level outcomes.

This caution is consistent with recent empirical and review-based evidence showing that the realized economic and environmental benefits of precision agriculture are heterogeneous and strongly context dependent, and that practical uptake of field robots remains shaped by factors such as investment cost, reliability, and farm-specific fit[32–35].

Some other comments:

Comment 2.9

Some of the economic input data, particularly the costs of organic systems (e.g. Brumfield, 1996; Tourte et al., 2009), are based on rather old sources. In light of the substantial changes in agricultural cost structures over the past 15–30 years, it would be beneficial to verify their relevance or supplement the analysis with more recent data.

Response 2.9

We appreciate this comment and re-checked the underlying data sources during revision, including a specific search for more recent references. For organic onions and lettuce, however, we were unable to identify newer sources with sufficiently detailed and comparable data to support a consistent replacement in the benchmarking analysis. We therefore retained the original references and continue to note in the manuscript that the underlying reference values for organic onions and lettuce were adjusted for inflation because no recent data were available (Lines 261-263). We believe this is the most transparent approach, as replacing these sources with less detailed or less comparable data would reduce consistency across the benchmark comparison.

Comment 2.10

Figure 3 shows the TCO and GWP results together on one graph, with different y-axes and patterns. While this allows a lot of information to be included, it can make the results difficult to interpret and compare across crops and systems. It is recommended to consider presenting TCO and LCA results in separate graphs.

Response 2.10

We thank the reviewer for this suggestion. After considering it carefully, we chose to retain the combined presentation in Figure 3 because the figure is intended to support direct side-by-side comparison of cost and environmental impact decomposition for the same crop and production-system combinations. This makes it easier to relate the contribution of individual components across TCO and GWP within each scenario, whereas splitting the results into separate figures would make these comparisons less direct and require substantially more figure space.

Comment 2.11

Figure 2. A wheeled chassis is modeled, but the illustration shows a tracked prototype, which raises questions in general and regarding the modeling of traction, stability, and contact with the ground.

Response 2.11

We thank the reviewer for pointing out this potential source of confusion. As discussed in more detail in Response 3.2, we have clarified the intended relationship between the modeled platform and the robot examples shown in the manuscript. To avoid implying a closer correspondence between the modeled ABAR and the tracked prototype than intended, the image of the tracked robot was removed, and Figure 2 now shows only the modeled platform representation. The related traction assumptions and operating parameters were revised separately and are discussed in Response 2.5.

Comment 2.12

I think the article is over 5000 words, but maybe I'm not measuring accurately...

Response 2.12

We thank the reviewer for noting this. The revised manuscript remains within the Nature Communications word limit. Under the journal's format, the 5,000-word limit applies to the combined Introduction, Results, and Discussion sections[36], which together are approximately 4,600 words in the revised version.

Final remarks by the reviewer

Overall, the article is a strong and scientifically valuable piece of work with the potential to make a significant contribution to the field of autonomous agricultural technologies. However, the conclusions are currently based on a set of optimistic assumptions that could lead to an overly favourable assessment of the ABAR concept. Some of these assumptions should be substantiated further, while others should be assessed through sensitivity analysis.

Implementing the above recommendations would strengthen the work both methodologically and interpretively.

A structured version of this review is also provided as a supplementary attachment.

Reviewer #3

The combining of LCA and TCO in the field of Ag Automation is certainly a valuable piece of work given the current trajectories of these technologies. By concentrating on specialty crops and AgBots rather than tractors the work complements that of previous economic modelling publications, critically by James Lowenberg-DeBoer and his group.

The results at top level corroborate the findings of those exciting studies which have autonomous cost of production circa £140-120/T in wheat (£0.14/kg), which is encouraging.

The insight that variable components has greatest effect on LCA is very useful when considering the move to battery powered agricultural practice.

However there are a few areas of concern to address:

Comment 3.1

Firstly, the statement "Because the robot operates fully autonomously, personnel costs are excluded" this is certainly contrary to my extensive experience working in this area and differs from the other economics work mentioned above. Although worker interaction with a field task can be dramatically reduced it is not to 0, I strongly suggest some level of human interaction is considered in this work.

Response 3.1

We thank the reviewer for raising this important point. Because it closely mirrors Reviewer #2's concern about labor treatment, we refer to Response 2.7 for the full revision details. Briefly, labor is now included in OPEX through a supervision-based formulation, reflecting that even autonomous operation still requires setup, monitoring, occasional intervention, and logistical support.

Comment 3.2

Secondly, the paper shows how important robot parameters (eg size, speed etc) are, I question some of these points used in the calculations. Critically the base forward speed listed for ploughing and seeding seems optimistic for the zauberzeug Field Friend when their own videos are considered. Of the two robots referenced zauberzeug and FarmDroid I would see the later as a better example to base work on due to its commercial footprint and extensive field time which may better guide baseline values, if commercial farm users are considered. It should also be noted most of this type of "Ag Bots" are establishing crops in land that has been pre tilled by conventional tractors and are not doing the primary tillage themselves (currently).

Response 3.2

We thank the reviewer for these helpful points regarding both the operating-speed assumptions and the interpretation of the robot examples shown in the manuscript. We agree that the original values used for soil preparation and sowing were too optimistic for a compact platform of this type. In response, and consistent with the traction-related revision described in Response 2.5, these parameters were re-evaluated and reduced from 1.5 to 1 km h^{-1} for soil preparation and from 4 to 3 km h^{-1} for sowing. These changes are now reflected in the revised parameterization and contribute to a more mechanically consistent representation of the modeled platform under traction-requiring field conditions.

We also appreciate the reviewer's observation that current commercial systems such as the FarmDroid are more commonly used in pre-tilled fields and do not typically perform primary tillage themselves. We acknowledge this point. In the present study, however, soil preparation was retained within the system boundary because the aim was to represent cradle-to-farm-gate production as completely as possible. The revised, more conservative parameterization now shows that heavy soil-engaging tasks can strongly constrain annual throughput (and therefore total obtainable crop yield) in a compact single-ABAR configuration. This also suggests that future studies of small ABARs may choose to exclude soil preparation from the system boundary when focusing on more commercially established use cases (see Lines 426-430):

Future models of small ABARs may also choose to exclude soil preparation from the system boundary, as heavy soil-engaging operations can strongly constrain annual throughput in compact single-ABAR configurations. This step was nevertheless deliberately retained in the present study so that the associated limitation would remain visible.

Finally, this comment helped us recognize that the original presentation may have suggested a closer link to specific commercial robots than intended. The modeled ABAR is not a one-to-one representation of either the Zauberzeug Field Friend or FarmDroid, but a simplified parameterized platform informed by recent autonomous farming robots (Lines 79-84, also see Response 1.6):

The ABAR model in this study reflects recent industrial autonomous farming robots[1,2], but is intended as a simplified, parameterized representation rather than a one-to-one model of any specific product (Figure 2). Its dimensions (0.85 m × 0.70 m × 0.70 m; L × W × H) are based on an existing compact ABAR platform[1] to anchor the geometry, while the included components, their sizing, and the operational parameterization were defined for this study based on the assumptions described in Methods.

To avoid creating the impression that the calculations are directly based on the Field Friend, the image of that robot was removed from Figure 2, which now shows only the modeled platform representation.

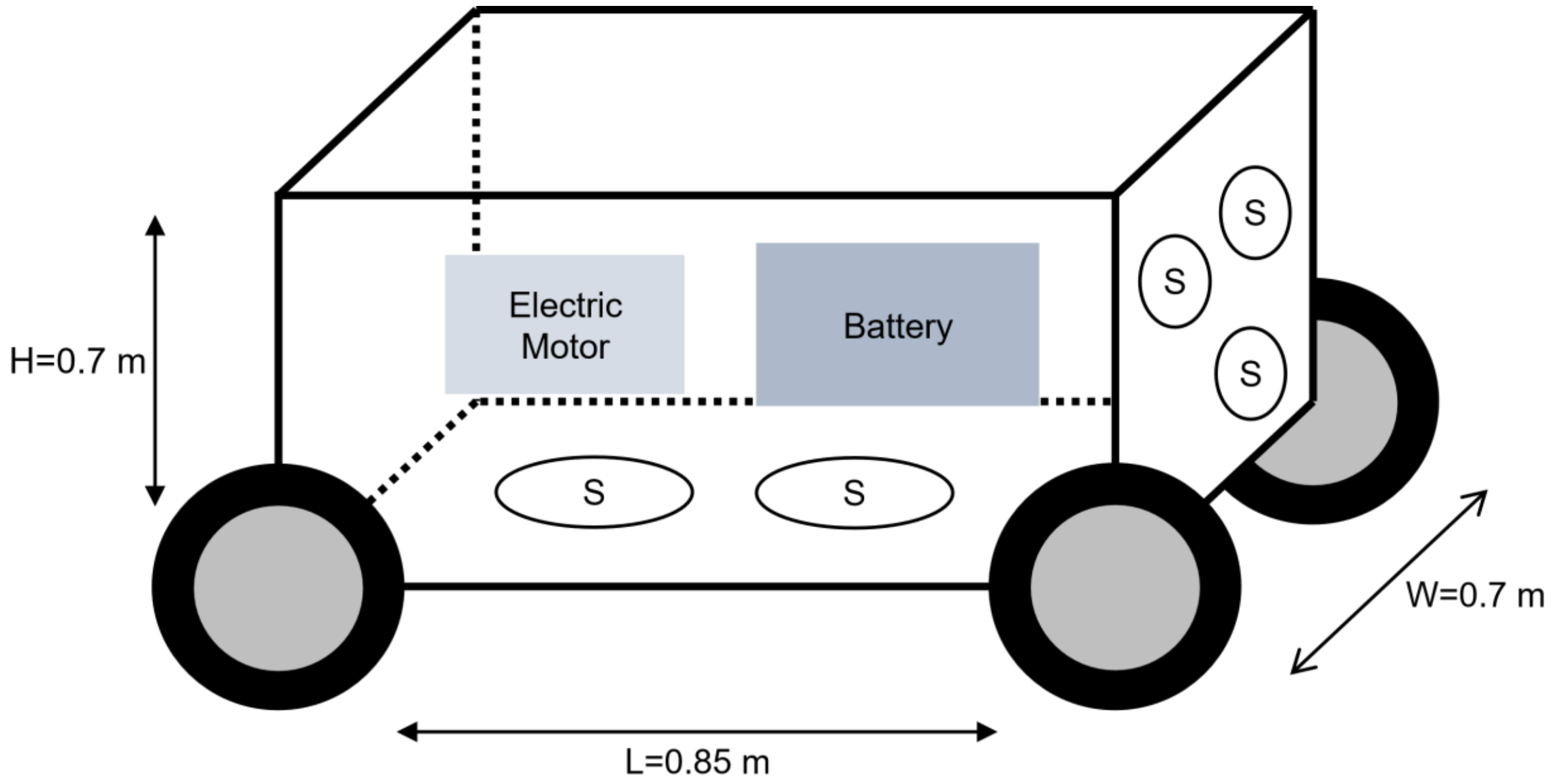

Figure 4: Schematic drawing of the modelled autonomous battery-powered farming robot (S=sensor).

Comment 3.3

Finally the idea that irrigation can be applied via a 500L tank is impossibly optimistic, even with the 5% application rate you use (seen in Eq 12) 250,000L/ha needs to be applied in the case of strawberries equating to 500 robot trips/ha/year or 2500 across 5ha you say is achievable. I also question the idea that 5% irrigation is achievable without having a yield effect and am not sure the opinion of a robotics company is good enough reference for this decision.

I would agree 50% pesticide and fertilizer reduction is possible

Response 3.3

We thank the reviewer for this important observation. As noted in Response 2.6, we agree that the original 95% irrigation-reduction assumption was too optimistic, and we have therefore revised the base-case water reduction from 95% to 50%, while continuing to explore variation around this value in the sensitivity analysis. We also agree that, even under the original assumption, a 500 L onboard tank would not be sufficient to deliver the modeled irrigation volumes, and that this issue becomes even more pronounced under the revised assumption. In the present model, however, the tank is intended as a simplified proxy for intermediary storage within the irrigation module for TCO and LCA purposes, rather than as a detailed engineering representation of fully self-contained irrigation logistics. Continuous replenishment from external water supply while the robot is operating in the field is therefore implicitly assumed. Under this interpretation, the modeled ABAR represents precision water application rather than bulk water transport. The manuscript now clarifies this point

more explicitly, states that external water-supply infrastructure and refill logistics are not resolved in detail within the present higher-level modeling framework, and revises the corresponding tool descriptions in the main text and Supplementary Information to emphasize their proxy character rather than specific storage volumes:

Lines 401-404: Likewise, liquid-application tasks such as irrigation and chemical weed control are represented through simplified storage and application proxies, and the model does not resolve external supply infrastructure or refill logistics in detail.

Lines 66-68: The model includes simplified tool and module proxies for preparation, sowing, mechanical and chemical weed control, irrigation, and fertilization, which together define the cradle-to-farm-gate system boundary summarized in **Error! Reference source not found.**.

Lines 92-99: To estimate task-level emissions and costs more accurately, the required tools are represented by simplified proxies. Where appropriate, these are scaled to the robot's geometry and informed by existing technologies. They include a proxy for soil preparation, mountable seeding units, a cultivator for mechanical weed control, a boom sprayer for chemical weed control, and a trailer-mounted water bowser for irrigation. Fertilization is assumed to occur concurrently with irrigation and therefore does not require an additional tool. Further details on the robot, ballast and proxy tools are provided in Supplementary Note 1 of the supporting materials.

Supplementary Note 1: All listed components and tools serve as simplified proxies for the modeled robot configuration and, where appropriate, were scaled down accordingly.

Table S1: Overview of the modelled proxy components, ballast, and tools, including data sources.

**Component/Tool for robot task**	**Specific name and source**
Battery	Lithium Nickel-Manganese-Cobalt-Oxide (NMC 811) battery, 600 $Wh \cdot L^{-1}$, 250 $Wh \cdot kg^{-1}$ [37]
Electric motor	1 kW brushless DC motor[38]
Wheels	13x5.00-6 Lug tires[39]
Sensors	i) RTK-1010 High-Performance Dual-Band GNSS RTK Module[40]<br>ii) SICK 2D-LiDAR-Sensor[41]<br>iii) Zed 2i RGB-D camera[42]<br>iv) MTI-7 GNSS/INS inertial measurement unit[43]<br>v) Microcontroller/CPU[44]
Preparation	Scaled-down disk harrow[45] and steel ballast
Sowing	Bar mounted grain seeder[46] and steel ballast
Mechanical weed control	C-tine cultivator[47]
Chemical weed control	Tank and mounted boom sprayer[48]
Irrigation and fertilization	Trailer-mounted water bowser[49]

Final remarks from the author team

We are sincerely grateful for the time, care, and expertise the reviewers devoted to this manuscript. Their comments were thoughtful, thorough, and technically insightful, and we believe they have substantially improved the quality, clarity, and robustness of the revised manuscript. We greatly appreciate the reviewers' constructive engagement and the opportunity to strengthen the paper through this process.

References

1. Zauberzeug GmbH. Field Friend. Available at https://zauberzeug.com/products/field-friend (2025).
2. FarmDroid. FarmDroid FD20 - Autonomous Farming Robot for Precision Agriculture. Available at https://farmdroid.com/products/farmdroid-fd20/ (2025).
3. Fairuz, S. A., Chen, G. & Maraseni, T. Driving change in agriculture (2020–2025): A global review of LCA findings and adoption factors for agricultural electric vehicles. *Renewable Sustainable Energy Rev.* **226,** 116295; 10.1016/j.rser.2025.116295 (2026).
4. Lagnelöv, O., Larsson, G., Larsolle, A. & Hansson, P.-A. Life Cycle Assessment of Autonomous Electric Field Tractors in Swedish Agriculture. *Sustainability* **13,** 11285; 10.3390/su132011285 (2021).
5. Guo, J. *et al.* Technology progress in mechanical harvest of fresh market strawberries. *Computers and Electronics in Agriculture* **226,** 109468; 10.1016/j.compag.2024.109468 (2024).
6. Kumawat, L. & Raheman, H. Mechanization in Onion Harvesting and its Performance: A Review and a Conceptual Design of Onion Harvester from Indian Perspective. *J. Inst. Eng. India Ser. A* **103,** 295–304; 10.1007/s40030-021-00611-3 (2022).
7. Liu, R., Dong, M., Xiao, Q., Lv, C. & Zhang, Z. Design, Analysis, and Experiment of an Automatic Lettuce-Harvesting Mechanism. *Chin. J. Mech. Eng.* **38,** 175; 10.1186/s10033-025-01288-6 (2025).
8. Moreira, Bruno Rafael de Almeida *et al.* Advancements in peanut mechanization: Implications for sustainable agriculture. *Agricultural Systems* **215,** 103868; 10.1016/j.agsy.2024.103868 (2024).
9. Clark, S. & Mousavi-Avval, S. H. Global Warming Potential of Organic Strawberry Production under Unheated High Tunnels in Kentucky, USA. *Sustainability* **14,** 1778; 10.3390/su14031778 (2022).
10. ecoinvent. ecoQuery - ecoinvent 3.11, cutoff. Available at https://ecoquery.ecoinvent.org/3.11/cutoff? (2025).
11. Esmaeilzadeh, S., Asgharipour, M. R. & Khoshnevisan, B. Water footprint and life cycle assessment of edible onion production - A case study in Iran. *Scientia Horticulturae* **261,** 108925; 10.1016/j.scienta.2019.108925 (2020).
12. Abdelkader, M., Zargar, M., Murtazova, K. M.-S. & Nakhaev, M. R. Life Cycle Assessment of the Cultivation Processes for the Main Vegetable Crops in Southern Egypt. *Agronomy* **12,** 1527; 10.3390/agronomy12071527 (2022).
13. Hall, G. *et al.* Potential environmental and population health impacts of local urban food systems under climate change: a life cycle analysis case study of lettuce and chicken. *Agric & Food Secur* **3**; 10.1186/2048-7010-3-6 (2014).

14. Gargaro, M., Hastings, A., Murphy, R. J. & Harris, Z. M. A Comparative LCA of Field Grown Lettuce Versus Vertically Farmed Lettuce. *Food and Energy Security* **14**; 10.1002/fes3.70117 (2025).

15. Plawecki, R., Pirog, R., Montri, A. & Hamm, M. W. Comparative carbon footprint assessment of winter lettuce production in two climatic zones for Midwestern market. *Renew. Agric. Food Syst.* **29,** 310–318; 10.1017/S1742170513000161 (2014).

16. Nikkhah, A., Khojastehpour, M., Emadi, B., Taheri-Rad, A. & Khorramdel, S. Environmental impacts of peanut production system using life cycle assessment methodology. *J. Clean. Prod.* **92,** 84–90; 10.1016/j.jclepro.2014.12.048 (2015).

17. European Commission. The European Green Deal, 06.03.2024.

18. Lander, L. *et al.* Cost and carbon footprint reduction of electric vehicle lithium-ion batteries through efficient thermal management. *Appl. Energy* **289,** 116737; 10.1016/j.apenergy.2021.116737 (2021).

19. Deutz, S. & Bardow, A. Life-cycle assessment of an industrial direct air capture process based on temperature–vacuum swing adsorption. *Nat Energy* **6,** 203–213; 10.1038/s41560-020-00771-9 (2021).

20. Krupanek, J. *et al.* Environmental performance of an autonomous laser weeding robot—a case study. *Int J Life Cycle Assess* **29,** 1021–1052; 10.1007/s11367-024-02295-w (2024).

21. Pradel, M., Fays, M. de & Seguineau, C. Comparative Life Cycle Assessment of intra-row and inter-row weeding practices using autonomous robot systems in French vineyards. *The Science of the total environment* **838,** 156441; 10.1016/j.scitotenv.2022.156441 (2022).

22. Saidani, M. *et al.* Assessing the environmental and economic sustainability of autonomous systems: A case study in the agricultural industry. *Procedia CIRP* **90,** 209–214; 10.1016/j.procir.2020.02.123 (2020).

23. Vahdanjoo, M., Gislum, R. & Sørensen, C. A. G. Operational, Economic, and Environmental Assessment of an Agricultural Robot in Seeding and Weeding Operations. *AgriEngineering* **5,** 299–324; 10.3390/agriengineering5010020 (2023).

24. Shang, L., Pahmeyer, C., Heckelei, T., Rasch, S. & Storm, H. How much can farmers pay for weeding robots? A Monte Carlo simulation study. *Precision Agric* **24,** 1–26; 10.1007/s11119-023-10015-x (2023).

25. Queiroz, D. M. & Schueller, J. K. Traction. In *Introduction to Biosystems Engineering,* edited by N. M. Holden, M. L. Wolfe, J. A. Ogejo & E. J. Cummins (American Society of Agricultural and Biological Engineers (ASABE) in association with Virginia Tech Publishing, Virginia Tech Libraries2021), pp. 181–194.

26. Papadopoulos, G. *et al.* Economic and environmental benefits of digital agricultural technologies in crop production: A review. *Smart Agricultural Technology* **8,** 100441; 10.1016/j.atech.2024.100441 (2024).

27. Oguztürk, G. E. AI-driven irrigation systems for sustainable water management: A systematic review and meta-analytical insights. *Smart Agricultural Technology* **11,** 100982; 10.1016/j.atech.2025.100982 (2025).

28. Sørensen, C. G. & Nielsen, V. Operational Analyses and Model Comparison of Machinery Systems for Reduced Tillage. *Biosystems Engineering* **92,** 143–155; 10.1016/j.biosystemseng.2005.06.014 (2005).

29. United Nations Environment Programme. Guidelines for social life cycle assessment of products, 2009.

30. Maritan, E., Lowenberg-DeBoer, J., Behrendt, K. & Franklin, K. Economically optimal farmer supervision of crop robots. *Smart Agricultural Technology* **3,** 100110; 10.1016/j.atech.2022.100110 (2023).

31. Lampridi, M. G. *et al.* A Case-Based Economic Assessment of Robotics Employment in Precision Arable Farming. *Agronomy* **9,** 175; 10.3390/agronomy9040175 (2019).

32. Bahmutsky, S., Grassauer, F., Arulnathan, V. & Pelletier, N. A review of life cycle impacts and costs of precision agriculture for cultivation of field crops. *Sustainable Production and Consumption* **52,** 347–362; 10.1016/j.spc.2024.11.010 (2024).

33. Kroupová, Z. Ž. *et al.* Drivers and barriers to precision agriculture technology and digitalisation adoption: Meta-analysis of decision choice models. *Precision Agric* **26,** 17; 10.1007/s11119-024-10213-1 (2025).

34. Ruder, S.-L., Faxon, H. O., Orzel, E. C., Devkota, R. & Bronson, K. Reviewing the evidence on precision agriculture and environmental sustainability. *npj Sustain. Agric.* **4,** 9; 10.1038/s44264-026-00128-x (2026).

35. Tamirat, T. W., Pedersen, S. M., Ørum, J. E. & Holm, S. H. Multi-stakeholder perspectives on field crop robots: lessons from four case areas in Europe. *Smart Agricultural Technology* **4,** 100143; 10.1016/j.atech.2022.100143 (2023).

36. Nature Communications. Article in Nature Communications. Available at https://www.nature.com/ncomms/submit/article (2026).

37. Ruppert, J. *et al.* Analyzing material and production costs for lithium-ion and sodium-ion batteries using process-based cost modeling - CellEst 3.0. *J. Power Sources Adv.* **36,** 100190; 10.1016/j.powera.2025.100190 (2025).

38. Stepperonline. Bürstenloser Gleichstrommotor mit Kühlerlüfter - 80BLS160F-310-02. Available at https://www.omc-stepperonline.com/de/310v-6000u-min-1-75nm-1100w-4-5a-quadratisch-80-x-80-x-159mm-buerstenloser-gleichstrommotor-mit-kuehlerluefter-80bls160f-310-02 (2026).

39. Deli Tire. Tyre 13 x 5.00-6, 4PR, TL, Deli S-247 - AS for lawn tractor, ride-on lawn mower. Available at https://www.amazon.de/Reifen-13x5-00-6-ST-45-Rasentraktor-Aufsitzrasenm%C3%A4her/dp/B00KNZI7CA?source=ps-sl-shoppingads-lpcontext&ref_=fplfs&psc=1&smid=AEV2V27JI6GWW (2025).
40. Mobile GPS Online. RTK-1010 High-Performance Dual-Band GNSS RTK Module. Available at https://mobilegpsonline.ca/products/rtk-1010-high-performance-dual-band-gnss-rtk-module?variant=40223677841496 (2025).
41. ETOH24. SICK 2D-LiDAR-Sensor TIM100-3010200. Available at https://www.etoh24.de/sick-2d-lidar-sensor-tim100-3010200.html (2025).
42. Stereolabs. ZED 2 - AI Stereo Camera (2025).
43. Farnell. MTI-7-0I-DK XSENS, Development Kit. Available at https://de.farnell.com/en-DE/xsens/mti-7-0i-dk/dev-kit-3-achs-beschl-messer-gyro/dp/3793985 (2025).
44. Lenovo. Lenovo ThinkStation P360 Tiny 30FA004WGE. Available at https://www.amazon.de/dp/B0BRNP7T8Y?m=A3JWKAKR8XB7XF&tag=idealode-am-pk-21&ascsubtag=2025-08-29_59fa4766bde37a3a1b97c7a4a49394218db497964e609700802cbede8ca677e3&th=1&psc=1 (2025).
45. Everythingattachments. Compact Tractor Angle Iron Disc Harrow. Available at https://www.everythingattachments.com/Compact-Tractor-Angle-Iron-Disc-Harrow-p/eta-xdcmp-dh.htm (2026).
46. Terradonis. JPH bar mounted small grain seeeder. Available at https://www.terradonis.com/an-terradonis-jph-small-grain-seeder.html (2025).
47. TOPREGAL. GK6 cultivator for small tractor, 6 tines, TecMaschin. Available at https://www.topregal.com/en/forklift-tractor-attachments/gk6-cultivator-for-small-tractor-6-tines-tecmaschin.html (2025).
48. Machinery Sales EU. CROP SPRAYER 300l tank 6m boom. Available at https://machinerysaleseu.co.uk/product/crop-sprayer-300l-boom-6m/ (2025).
49. Online Tank Store. 500 Litre Water & Drinking Water Highway Bowser. Available at https://www.onlinetankstore.co.uk/product/500-litre-water-drinking-water-highway-bowser/ (2025).

Dear Reviewers,

Thank you for your final comments. We are pleased to hear that you feel the revised manuscript has substantially improved. Below, we provide a point-by-point response and indicate the corresponding revisions to the manuscript.

Reviewer #1

Comment 1.1

The authors have addressed my main comments and the manuscript has improved substantially. The revised version is clearer about its exploratory, scenario-based character and now better acknowledges key assumptions and limitations. I only encourage the authors to ensure that the conclusions clearly distinguish the effects of the robotic platform from those of the assumed reductions in inputs and labour, which appear to drive much of the reported benefit. In addition, comparisons with conventional systems should remain cautiously framed as comparisons with literature-based reference ranges rather than direct predictions for specific farming contexts.

Response 1.1

Thanks! We agree that comparisons with conventional systems should be framed more cautiously as comparisons with literature-based reference ranges. Accordingly, we have revised several passages throughout the manuscript to distinguish more clearly between the effects of the robotic platform and those of reduced labor and input use, and to frame comparisons with conventional systems explicitly as literature-based reference ranges. We have also added a corresponding limitation to the discussion:

Lines 19-21: Across modeled scenarios, benchmarked costs range from 76% below to 17% above literature-based reference ranges, while greenhouse-gas emissions fall within or below reported ranges.

Lines 247-249: The following analysis therefore compares ABAR-based production with literature-based reference ranges for conventional systems using published data for both TCO and LCA.

Lines 347-351: Under the modeled conditions, ABAR-based production can be cost competitive compared to conventional crop production ranges reported in prior literature while also reducing greenhouse-gas emissions per kilogram of crop, particularly where precision management lowers demand for variable inputs.

Lines 379-382: This suggests that, in the modeled scenarios, the main benefits arise less from the platform alone than from more precise management, the potential to reduce labor inputs, and the associated reductions in seed, fertilizer, pesticide, and water use.

Lines 425-429: Another limitation concerns the comparisons with conventional systems, for which this study benchmarked against literature-based reference ranges rather than direct predictions for specific farming contexts. Future research is,

therefore, encouraged to benchmark against data from robots that generate precise, localized predictions in specific farming contexts.

Reviewer #2

General assessment

The revised version of the manuscript is significantly improved compared to the first version. The authors have responded constructively and consistently to most of the reviewers' comments and have strengthened both the methodological soundness and the balance in the interpretation of the results. I particularly appreciate that several key model assumptions have been revised, including the robot's service life, the extent of water-use reduction, the treatment of labor costs, and the modeling of tractor-requiring operations.

The revised manuscript now makes it much clearer that the presented results should be interpreted as exploratory system-level scenarios rather than direct forecasts for actual farms. The discussion of the model's limitations, technological uncertainty, and practical application constraints has also been significantly strengthened. In my opinion, the manuscript has become considerably more balanced, transparent, and scientifically mature.

The authors also more clearly distinguish the impact of the robotic platform itself from the agronomic assumptions related to more precise input management. This significantly improves the overall interpretation of the results.

Below are only a few remaining comments that could further strengthen the manuscript.

Comment 2.1

Crop selection and comparability of scenarios

In the revised version, the authors explain more clearly why highly mechanized commodity crops were not included and why the selected crops are considered potentially suitable for compact ABAR systems. However, the analyzed crops still represent fairly heterogeneous production systems with substantially different agronomic, operational, and especially harvesting characteristics. Although this broadens the exploratory scope of the model, it also increases dependence on crop-specific assumptions and reduces direct comparability across scenarios. It would therefore be useful to briefly acknowledge this trade-off between modeling generality and the realism of specific production systems in the Discussion section.

Response 2.1:

Thanks! Following your comment, in the revised Discussion, we now acknowledge more explicitly that the broad crop selection increases the generality of the analysis but reduces the realism and direct comparability of individual scenarios, as each crop requires distinct agronomic and operational assumptions (Line 354-357):

At the same time, this broad crop selection increases the generality of the analysis but reduces the realism and direct comparability of individual scenarios, as each crop requires distinct agronomic and operational assumptions.

Comment 2.2

Software and digital infrastructure costs

I appreciate that the authors now clearly acknowledge that the costs associated with external software, cloud computing, connectivity, and data-management infrastructure were not included in the analysis. However, the statement that including these components would likely not substantially alter the main conclusions remains somewhat qualitative, as these costs were not directly modeled. I believe this interpretation could be presented somewhat more cautiously.

Response 2.2

We agree that this should be presented more cautiously. We have therefore revised the paragraph to present this point more cautiously and now explicitly state that the influence of external AI and software costs cannot be fully assessed without direct modeling and requires further investigation (Lines 416-419):

A further limitation is that the analysis excludes costs associated with the external AI and software infrastructure needed to operate the robot, such as cloud computing, data management, connectivity, and software licensing. These costs were omitted because they are highly deployment-specific and difficult to parameterize consistently across scenarios. While the modeled results suggest that variable crop-production inputs are likely to remain an important cost driver, the potential influence of external AI and software costs cannot be fully assessed without direct modeling and warrants further investigation.

Reviewer #3

Comment 3.1:

The authors have put considerable effort into responding and amending the areas of concern raised by myself and the fellow reviewers. While the specifics of the amendments may be argued further I believe the paper now presents in publishable form and provides useful insight, combining of LCA and TCO in the field of Ag Automation.

Response 3.1:

Thank you for your positive assessment and your constructive feedback throughout the review process.
